# High-precision and low-depth quantum algorithm design for eigenstate problems

**DOI:** 10.1126/sciadv.aeb1622

**Published:** 2026-01-16

**Authors:** Jinzhao Sun, Pei Zeng, Tom Gur, M. S. Kim

**Affiliations:** ^1^ Blackett Laboratory, Imperial College London, London SW7 2AZ, UK.; ^2^ Computer Laboratory, University of Cambridge, Cambridge CB3 0FD, UK.; ^3^ School of Physical and Chemical Sciences, Queen Mary University of London, London E1 4NS, UK.; ^4^ Pritzker School of Molecular Engineering, The University of Chicago, Chicago, IL 60637, USA.

## Abstract

Estimating the eigenstate properties of quantum systems is a long-standing, challenging problem for both classical and quantum computing. Existing universal quantum algorithms typically rely on ideal and efficient query models (e.g., time evolution operator or block encoding of the Hamiltonian), which, however, become suboptimal for actual implementation at the quantum circuit level. Here, we present a full-stack design of quantum algorithms for estimating the eigenenergy and eigenstate properties, which can achieve high precision and good scaling with system size. The gate complexity per circuit for estimating generic Hamiltonians’ eigenstate properties is $\tilde{O}\left(\text{log}\varepsilon^{-1}\right)$, which has a logarithmic dependence on the inverse precision ε. For lattice Hamiltonians, the circuit depth of our design achieves near-optimal system-size scaling, even with local qubit connectivity. Our full-stack algorithm has low overhead in circuit compilation, which thus results in a small actual gate count (cnot and non-Clifford gates) for lattice and molecular problems compared to advanced eigenstate algorithms. The algorithm is implemented on IBM quantum devices using up to 2000 two-qubit gates and 20,000 single-qubit gates and achieves high-precision eigenenergy estimation for Heisenberg-type Hamiltonians, demonstrating its noise robustness.

## Teaser

A full-stack quantum algorithm design achieves near-optimal depth scaling for estimating many-body eigenstate properties.

## INTRODUCTION

Estimating the properties of the ground and excited states of quantum many-body systems is a long-standing problem of fundamental interest, which has applications in condensed matter physics, quantum chemistry, and material science (*1*–*3*). Despite its quantum hardness both in theoretical complexity (*4*) and empirical numerical evidence (*5*), finding eigenstates of many-body Hamiltonians remains a central goal, driving ongoing exploration of quantum algorithms, from quantum phase estimation (QPE) (*6*–*13*) to spectral filter algorithms (*14*–*31*), dissipation-based algorithms (*32*–*34*), and others (*35*–*39*). In particular, with rapid development of quantum hardware and error correction, there is increasing interest in designing quantum algorithms considering the feature of early fault-tolerant quantum computing (FTQC) devices (*21*, *40*), where minimizing controlled operations and circuit depth is essential. This constraint also applies to noisy intermediate-scale quantum (NISQ) devices. In this context, it is desirable to design quantum algorithms that satisfy the constraints of fewer qubits, low circuit depth, and restricted qubit connectivity, which are often interrelated when compiling nonlocal controlled gates into local ones (*41*).

Considering the above hardware constraints (*21*), spectral filter–based methods are good candidates for effectively finding the ground state (*12*, *18*–*26*), which can achieve good asymptotic query complexity under the assumptions of nonvanishing initial overlap and energy gap. These include algorithms for ground-state energy estimation (*21*) and property estimation (*22*, *24*), as well as subsequent developments (*12*, *15*, *18*–*20*). Nonetheless, these algorithms are typically based on perfect and efficient queries to either the real-time evolution operator *e^–iHt^*, which is the case for most early FTQC algorithms (*12*, *13*, *18*–*26*), or the block encoding of the Hamiltonian *H* (*28*, *42*). Their favorable scaling properties no longer exist when they are compiled into elementary gates in the quantum circuit. For example, implementing real-time evolution via Trotterization as a subroutine will eliminate the advantage of logarithmic precision scaling in eigenstate property estimation. On the other hand, realization via block-encoding will lose the good system-size scaling; moreover, it requires many ancillary qubits and nonlocal controlled gates, which violates the spirit of early FTQC. Moreover, the system-size dependence of the algorithms is rarely discussed in existing works, as it highly depends on the circuit-level implementation as well as the qubit connectivity of quantum devices. These algorithms thus become suboptimal at the quantum circuit level. An important question is, when considering the gate complexity and qubit connectivity in NISQ and early FTQC applications, how to design high-precision and low-depth algorithms.

In this work, we present a full-stack quantum algorithm for eigenstate property and eigenenergy estimation on the basis of randomized composite linear-combination-of-unitaries (LCU) formulae. The maximum gate complexity for each circuit at a single run is shown to be $O\left(\text{poly}\text{log}\left(\varepsilon^{-1}\right)\right)$, outperforming the QPE-based method and matching the result by quantum signal processing (QSP) (*28*, *42*). This precision scaling may not be achieved by coherent methods relying on a coherent implementation of real-time evolution, such as a variant of QSP (*28*) by quantum eigenvalue transformation of unitaries (QETU) (*43*). The second advantage of our method is its low circuit depth for various physical problems with conserved symmetries. For lattice Hamiltonians, our method achieves near-optimal system-size gate complexity per circuit even with nearest-neighbor (NN) qubit connectivity. Specifically, the depth complexity $d=O\left(n^{o\left(1\right)}\right)$ is nearly independent of *n*, besides the implicit dependence from the energy gap, which outperforms the other strategies. Here, the small-*o* notation *o*(1) indicates approaching to zero asymptotically. To accomplish this, we design new composite LCU circuits that maintain the system’s symmetries. Thanks to the integrated feature of the full-stack design, we are able to achieve near-optimal precision and system-size scaling, even when restricted to NN architecture. A comparison with representative advanced eigenstate algorithms is displayed in Table 1.

**Table 1.** Comparison of advanced methods for eigenstate property estimation on parameters of the systems (*L* and *n*) and target precision ε. The second column displays the maximum gate complexity in a single circuit instance for generic Hamiltonians with *L* terms. The third column displays the depth complexity for 1D Heisenberg Hamiltonians with qubit connectivity restricted to an NN architecture. The big-*O* and small-*o* notations are used. For example, $\tilde{O}$ denotes the complexity up to polylogarithmic factors as used in (*28*). Different methods have similar dependence on Δ, which is $\Delta^{-\left(1+o\left(1\right)\right)}$, and hence not included in this table. Tables S1 and Table S2 in section S2 present more detailed asymptotic scaling analyses for the eigenstate properties and energy estimation, respectively, which also include the dependence on other parameters λ and Δ. Note that our zeroth-order design with *k* = 0 is similar to (*17*), although our ancilla-free scheme preserves the advantage in circuit depth and the sampling procedure is simpler. When λ scales smaller than $O\left(\sqrt{L}\right)$, the zeroth-order case may be advantageous in Hamiltonian-parameter scaling.

Methods	Gate (generic model)	Depth (lattice)
This work	$O\left(L\text{log}\varepsilon^{-1}\right)$	$\tilde{O}\left(n^{o\left(1\right)}\varepsilon^{-o\left(1\right)}\right)$
(zeroth-order)	$O\left(\lambda^{2}{\text{log}}^{2}\varepsilon^{-1}\right)$	$O\left(n^{2}{\text{log}}^{2}\varepsilon^{-1}\right)$
QPE + Trotter	$O\left(L\varepsilon^{-\left(1+o\left(1\right)\right)}\right)$	$O\left(n^{1+o\left(1\right)}\varepsilon^{-\left(1+o\left(1\right)\right)}\right)$
QPE + QW (*52*, *53*)	$\tilde{O}\left(L\varepsilon^{-1}\right)$	$O\left(n^{2}\varepsilon^{-1}\right)$
QSP (*28*)	$O\left(L\text{log}\varepsilon^{-1}\right)$	$O\left(n^{2}\text{log}\varepsilon^{-1}\right)$
QETU (*43*)	$\tilde{O}\left(L\varepsilon^{-o\left(1\right)}\right)$	$O\left(n\varepsilon^{-o\left(1\right)}\right)$

An important feature of our full-stack algorithm is the low overhead in circuit compilation, resulting in a small actual gate count in each coherent circuit run. We present the resource requirements, including the cnot and non-Clifford gates, for representative physical models in condensed matter and chemistry. Prior resource estimates (*44*–*49*) are mostly based on QPE, which is further concatenated with either Trotterization (*13*, *50*, *51*) or qubitized quantum walks (QWs) (*52*–*54*). For lattice models and quantum chemistry problems, our method requires fewer gates compared to other advanced methods. In particular, the cnot gate cost for a 20-site Heisenberg model is on the order of 10^4^, while the T gate cost is about 10^6^. This makes our approach particularly suitable for NISQ and early FTQC applications. Our work also provides a useful toolbox for compiling state-of-the-art quantum algorithms into elementary gates. The algorithmic efficiency and inherent noise resilience of our approach enabled a high-precision implementation on IBM quantum devices with up to 2000 two-qubit gates and 20,000 single-qubit gates. We have tested several Heisenberg-type Hamiltonians with coupling strength *J* and achieved ground-state energy estimation errors of about 0.01*J*.

## RESULTS

### Randomized composite LCU formulae

Here, we introduce the framework of eigenenergy and eigenstate property estimation with the randomized composite LCU formulae. Let us start by formulating the problems and introducing the notations and assumptions used throughout this work. Consider an *n*-qubit gapped quantum system whose Hamiltonian *H* has a Pauli decomposition $H=\sum_{l=1}^{L}\alpha_{l}P_{l}≔\lambda \sum_{l=1}^{L}{\tilde{\alpha }}_{l}P_{l}$. Here, *P_l_* is a Pauli operator, $\lambda ≔\sum_{l}|\alpha_{l}|$, and $\tilde{\alpha }≔\alpha_{l}/\lambda$. The eigenstate $|u_{j}\rangle$ and the corresponding eigenenergy *E_j_* of the Hamiltonian satisfy $H|u_{j}\rangle =E_{j}|u_{j}\rangle$. The tasks concerned in this work are (i) to estimate the eigenenergy *E_j_* and (ii) to estimate an eigenstate property, characterized by an observable expectation on the target eigenstate $\langle u_{j}|O|u_{j}\rangle$. The assumptions as commonly used in QSP and other spectral filter methods are the following: (i) a good initial state $|\psi_{0}\rangle$ that has a nonvanishing overlap with the target eigenstate, $\eta ≔|\langle \psi_{0}{|u_{j}\rangle |}^{2}=\Omega \left(1/\text{poly}\left(n\right)\right)$; (ii) a nonvanishing energy gap $\Delta_{j}≔\text{min}\left(E_{j+1}-E_{j},E_{j}-E_{j-1}\right)$. The two eigenstate problems considered in this work are stated below.

Problem 1 **(Eigenstate property estimation)** *Suppose the observable has a Pauli decomposition as* $O=\sum_{l=1}^{L_{o}}o_{l}P_{l}$ *with Pauli operators P_l_ and positive coefficients o_l_ and* $∥O{∥}_{1}≔\sum_{l=1}^{L_{o}}|o_{l}|$. *Given an initial state* $|\psi_{0}\rangle$, *the aim is to find an estimator* $\hat{v}$*, such that it is close to* $\langle u_{j}|O|u_{j}\rangle$ *with probability at least* 1−ϑ*, i.e.,* $\text{Pr}\left(|\hat{v}-\langle u_{j}|O|u_{j}\rangle |\leq \varepsilon \right)\geq 1-\vartheta$.

Problem 2 **(Eigenenergy estimation)** *The aim is to find an eigenenergy estimator* ${\hat{E}}_{j}$*, such that it is* κ*-close to E_j_ with probability* 1−ϑ*, i.e.,* $\text{Pr}\left(|{\hat{E}}_{j}-E_{j}|\leq \kappa \right)>1-\vartheta$*.*

**Fig. 1.**
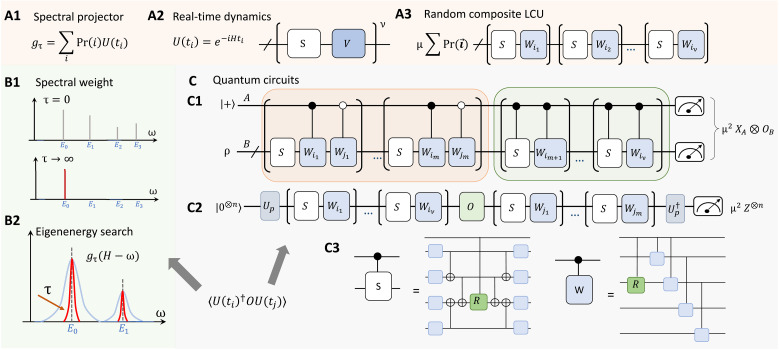
Workflow of the eigenstate property and eigenenergy estimation algorithm, from formula construction to circuit implementation. (**A**) The hierarchical structure of randomized composite LCU. (A1) The spectral filter is decomposed into an LCU with $U\left(t_{i}\right)=e^{-{\mathrm{iHt}}_{i}}$, described by Eq. 1. (A2) Each component *U*(*t_i_*) is divided into ν segments. Each segment with duration $t_{i}/\nu$ is realized by the Trotter formula *S* and the Trotter remainder *V* which is further decomposed into an LCU in Eq. 4, resulting in a composite LCU form. (A3) The overall explicit composite LCU form of *g*_τ_. The summands of the circuit are shown here; each circuit instance is sampled from the specified distribution in Eq. 5. Generally, $W_{i_{q}}$ is composed of one Pauli rotation operator $R=\text{exp}\left(-i\theta_{i_{q}}P_{i_{q}}\right)$ and a tensor product of Pauli operators. In the ancilla-free measurements in (C2), $W_{i_{q}}$ is a symmetry-conserved operator. The realization of controlled-*W* operation is shown in (C3). (**B**) Illustration of spectral filtering for effective eigenstate preparation and eigenenergy search. (B1) The spectral weight after applying the filter $g_{\tau }\left(H-E_{0}\right)$. *y* axis: the spectral weight $\langle u_{i}|g_{\tau }\left(H-E_{0}\right)|\psi_{0}\rangle$, with eigenstate $|u_{i}\rangle$ and eigenenergy *E_i_*. Increasing τ exponentially suppresses the spectral weight on excited states. (B2) The eigenenergy search by finding peaks of $D_{\tau }\left(\omega \right)=\langle \psi_{0}|g_{\tau }^{2}\left(H-\omega \right)|\psi_{0}\rangle$ (Eq. 11). The peaks become sharper with increasing τ (blue line → red line). (**C**) Quantum circuit implementation for measuring the sampled instance in $\langle U^{†}\left(t_{j}\right)OU\left(t_{i}\right)\rangle$, which is the key quantity in (B1) and (B2). (C1) Circuits for general Hamiltonians. Two extreme cases: the green box (*t_j_* = 0) and the orange box (*t_i_* = *t_j_*). Cases with *t_j_* < *t_i_* lie between and can be implemented with (C1). (C2) Ancilla-free measurement scheme for symmetry-conserved Hamiltonians. (C3) Compilation for controlled-*S* and controlled-*W* into cnot, single-qubit Pauli rotation (green), and Pauli gates (blue).

**Fig. 2.**
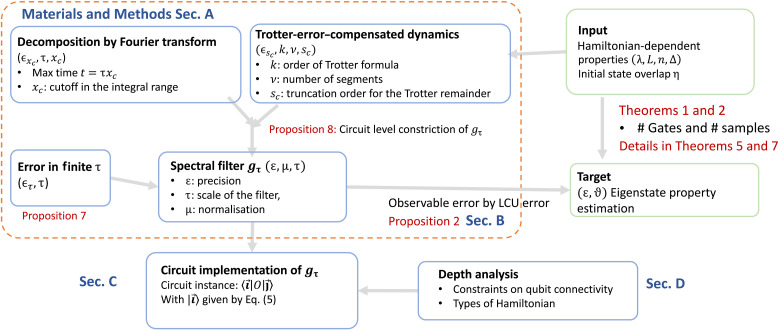
Illustration for the hierarchy of various components within the composite LCU. The figure illustrates the connections between different subsections in Materials and Methods. Sec. A, Composite LCU for the spectral filter; Sec. B, Observable estimation; Sec. C, Estimation by quantum circuits: General strategy and ancilla-free strategy; Sec. D, Analysis of circuit depth and gate complexity. This visualization aids in understanding how the target problem (Problem 1 and Problem 2) can be achieved, specifically, to achieve (ε,ϑ)-eigenstate property estimation. Arrows in the figure indicate direct connections between components. The key parameters relevant to the construction of LCU are highlighted.

The design of the eigenstate property and eigenenergy estimation algorithm is summarized in Algorithm 1 and illustrated in Fig. 1. To access the physical properties of eigenstates, a natural idea is to apply a spectral filter to the initial state that projects out the contributions from the other unwanted eigenstates, as illustrated in Fig. 1B1. While the spectral filter *g* is nonunitary by construction, we can effectively realize it using LCU techniques, either by coherent (*14*, *55*) or random-sampling approaches (*16*, *20*, *22*–*26*). The overall idea is that, at a higher level, the spectral filter is decomposed into a linear combination of unitaries $U\left(t_{i}\right)=e^{-{\mathrm{iHt}}_{i}}$, as shown in Fig. 1A1. We further realize each *U*(*t_i_*) by another random LCU formula. Specifically, the evolution is divided into ν segments, with each segment comprising both a Trotter formula term *S* and a Trotter remainder term *V*. Overall, it forms a composite LCU formula as illustrated in Fig. 1A3, which involves the summation and product of individual LCU components. The hierarchy of different LCU components is illustrated in Fig. 2, where the error propagation will be analyzed to prove the main theorems. The quantum circuit realization for randomized composite LCU is shown in Fig. 1C, including both the one-ancilla and ancilla-free schemes. The Hamiltonian-specific circuit compilation will be discussed in the “Analysis of circuit depth and gate complexity” section.

**Algorithm 1.**
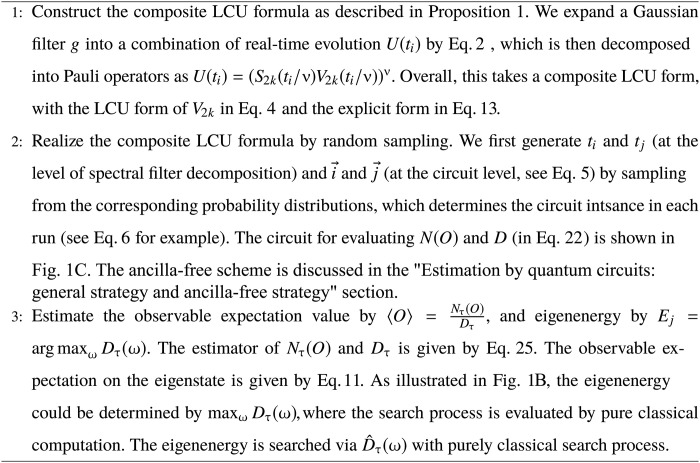
An overview of the random-sampling algorithm for Problem 1 and Problem 2.

The spectral filter *g* is a nonunitary operator defined on the *n*-qubit system, which is usually a function of the target Hamiltonian *H*. Choices for spectral filters include the imaginary-time evolution operator $g_{\tau }\left(H\right)=e^{-\tau H}$ or the Gaussian operator $g_{\tau }\left(H\right)=e^{-\tau^{2}H^{2}}$. For example, by applying $g_{\tau }\left(H\right)=e^{-\tau |H-E_{0}|}$ to an initial state $|\psi_{0}\rangle$, the unnormalized state becomes $|\psi \left(\tau \right)\rangle =c\left(\tau \right)\sum_{i}c_{i}e^{-|E_{i}-E_{0}|\tau }|u_{i}\rangle$ where *c*(τ) is the normalization factor. Provided the assumptions in Problem 1, i.e., a nonvanishing energy gap and a nonvanishing $c_{0}=\eta^{-1/2}$, the spectral weight of the unwanted excited eigenstates is exponentially suppressed with increasing τ. In the large τ limit, the state becomes the ground state ${\text{lim}}_{\tau \to \infty }|\psi \left(\tau \right)\rangle =c_{0}|u_{0}\rangle$. The procedure for obtaining the excited states is similar, as illustrated in Fig. 1B1. Note that the spectral filter method has been well established in the existing literature, in particular (*20*, *22*, *24*, *28*). In this work, we introduce a full-stack approach to eigenstate property and energy estimation by randomized composite LCU decomposition of a Gaussian operator into elementary gates. For simplicity, we refer to this full-stack randomized LCU approach as our method, abbreviated as RLCU.

Now, let us discuss the construction of RLCU. A (μ, ε)-randomized LCU formula, following the convention in the LCU formula (*56*, *57*), of a general operator *g* is defined to be

$$\tilde{g}=\mu_{g}\sum_{i}\text{Pr}\left(i\right)U_{i}$$ (1)

such that the spectral norm distance $∥g-\tilde{g}∥\leq \varepsilon$, as illustrated in Fig. 1A1. Here, μ > 0 is the normalization factor, Pr(*i*) is a probability distribution associated with an instance specified by *i*, and {*U_i_*}*_i_* is a group of unitaries. Equation 1 can be extended to a continuous form

$$\tilde{g}=\mu_{g}\int_{-x_{c}}^{+x_{c}}\mathrm{dxp}\left(x\right)U\left(x\right)$$ (2)

where we require that $p\left(x\right)\;\left(x\in \left[-x_{c},x_{c}\right]\right)$ is a well-defined probability distribution. A natural choice for *U_i_* is the real-time evolution $U_{i}=U\left(t_{i}\right)≔e^{-{\mathrm{iHt}}_{i}}$ with time length *t_i_* because quantum systems in nature evolve governed by the Hamiltonian. Then, one can decompose a nonunitary, Gaussian spectral filter on the basis of real-time evolution by setting the distribution $p\left(t\right)=\frac{1}{\sqrt{2\pi }}e^{-t^{2}/4}$. Although the Gaussian filter is represented in an integral form, it has a well-defined probability distribution and can be well-characterized by Eq. 1, which will be discussed in section S2.

While most early FTQC algorithms assume perfect implementation of real-time evolution, *U*(*t_i_*), in general, is not directly implementable at the quantum circuit level. An established way to implement *U*(*t_i_*) without involving other oracles is through Trotterization (*58*): When the Hamiltonian can be decomposed into Pauli operators, each *U*(*t_i_*) can be implemented using elementary quantum gates without additional qubit overhead. However, the issue with the Trotter method is that the remainder of a *k*th Trotter formula (i.e., Trotter error) is nonnegligible, which is polynomial in the order of *k* (*59*). As a result, any ground-state property estimation protocol based on Trotterization inevitably scales polynomially with the inverse of the target accuracy and loses the advantage of achieving high precision. Below, we show how to preserve the logarithmic dependence on the precision.

We consider dividing the time evolution operator *U*(*t_i_*) into ν segments, which can be written as $U\left(t_{i}\right)={\left(S\left(\delta t_{i}\right)V\left(\delta t_{i}\right)\right)}^{\nu }$ with $\delta t_{i}=t_{i}/\nu$. Here, *S*(δ*t_i_*) is a 2*k*th-order Trotter formula and *V*(δ*t_i_*) is the corresponding Trotter error within duration δ*t_i_*. Hereafter, the order 2*k* is omitted when there is no ambiguity. To preserve the high precision property, we choose to implement the Trotter remainder *V* as well, as opposed to implementing *S* only in conventional Trotter methods. By doing so, we can implement the spectral filter with high precision. Note that our algorithmic design supports the integration of advanced Hamiltonian simulation algorithms [e.g., (*60*, *61*)]. The benefits of using Trotter error compensation and comparison with other methods are discussed in section S1.

The spectral filter can now be formally rewritten as

$$\tilde{g}=\mu_{g}\sum_{i}\text{Pr}\left(i\right){\left(V\left(\delta t_{i}\right)S\left(\delta t_{i}\right)\right)}^{\nu }$$ (3)

which consists of other LCU formulae in it. Here, ν is chosen such that the approximation error is sufficiently small. We could see that Eq. 3 is a modified version of the original LCU formula given by Eq. 1, which involves hierarchical formulations, as illustrated in Fig. 1A. Specifically, it adopts a composite structure that integrates both the products of individual LCU components and the summations of LCU components. More concretely, suppose *g*_1_ takes a (μ_1_, ε_1_)-LCU formula of *g*, $g_{1}=\mu_{1}\sum_{i}\text{Pr}\left(i\right)U\left(t_{i}\right)$ in Eq. 1. Each summand *U*(*t_i_*) is divided into ν segments, with each segment *U*(δ*t_i_*) taking a (μ_2_, ε_2_)-LCU formula, specifically, $\tilde{U}\left(\delta t_{i}\right)=\mu_{2}\sum_{i_{\ell }}\text{Pr}\left(i_{\ell }\right)P_{i_{\ell }}$. To analyze the properties of the composite form of LCU formulae, we introduce the following proposition.

Proposition 1 **(Composite LCU)** *The formula* $g_{2}=\mu_{1}\sum_{i}\text{Pr}\left(i\right){\left(\tilde{U}\left(t_{i}/\nu \right)\right)}^{\nu }$ *is a* (μ, ε)*-LCU formula of g, with* $\mu ≔\mu_{1}\mu_{2}^{\nu }$*, and* $\varepsilon \leq \varepsilon_{1}+\mu_{1}{\mu \varepsilon }_{2}$*.*

Leveraging Proposition 1, we could calculate the error in the composite LCU form provided the error of each individual LCU approximation. This work will analyze the propagation of errors and the change of normalization factors within this composite LCU framework, outlined in Fig. 2. The proof is shown in section S2.

We shall briefly outline the implementation procedure as illustrated in Fig. 1 (A2 and A3). First, the Trotter remainder *V*(δ*t_i_*) is decomposed into easy-to-implement unitary operators

$$V=\mu \sum_{j}\text{Pr}\left(j\right)W_{j}$$ (4)

where Pr(*j*) is the probability associated with the unitary operator *W_j_*, μ is the associated normalization factor, and δ*t_i_* is omitted as this equation holds in general. The decomposition in Eq. 4 can be explicitly derived by *V* = *US*^†^ using the Taylor expansion. By doing so, we can compensate for the simulation error by sampling *W_j_* according to Pr(*j*) in Eq. 4. This effectively realizes a high-precision spectral filter, with the individual terms illustrated in Fig. 1A3. In general cases, the elementary operator *W_j_* in Eq. 4 can be chosen as Pauli operators or the exponentiation of Pauli operators, as commonly used in Trotter and LCU methods (*57*, *58*). The maximum gate count in the whole block of $W_{i_{q}}$ is saturated as ${\text{wt}}_{m}\left(H\right)+n$, where ${\text{wt}}_{m}H$ is the largest weight of the Hamiltonian terms (see Materials and Methods).

For systems with certain symmetry, the ancilla may not be needed. To enable ancilla-free measurement, we choose *W_j_* to be a symmetry-conserved operator, because choosing it as a Pauli operator will break the symmetry in the composite implementation. Specifically, to conserve the symmetry of either particle number or total spins, *W_j_* is chosen to be either the swap operator, Pauli-*Z* operator, or their exponentiation, detailed shortly after.

### Quantum circuit realization and depth analysis

The key quantity involved in the algorithm outlined in Fig. 1 is $\langle U^{†}\left(t_{j}\right)OU\left(t_{i}\right)\rangle$, where $U\left(t_{i}\right)=e^{-{\mathrm{iHt}}_{i}}$ and *O* = *I* for eigenenergy estimation with *O* being an Pauli operators for observable estimation. As introduced above, the unitary *U*(*t_i_*) is implemented by a composite LCU formula with an explicit form as

$$U\left(t_{i}\right)=\mu \sum_{\vec{i}}\text{Pr}\left(\vec{i}\right)\prod_{q=1}^{\nu }W_{i_{q}}S$$ (5)

where $\vec{i}=\left(i_{1},i_{2},\ldots ,i_{\nu }\right)$ and ν is the segment number. The observable expectation is thus given by

$$\langle U^{†}\left(t_{j}\right)\mathrm{OU}\left(t_{i}\right)\rangle =\mu^{2}\sum_{\vec{i},\vec{j}}\text{Pr}\left(\vec{i}\right)\text{Pr}\left(\vec{j}\right)\langle \vec{j}|O|\vec{i}\rangle$$ (6)

where we denote $|\vec{i}\rangle ≔\prod_{q=1}^{\nu }W_{i_{q}}S|\psi_{0}\rangle$.

For the general case, we can use the Hadamard test circuit to measure $\langle \vec{j}|O|\vec{i}\rangle$, with the circuit depicted in the green box in Fig. 1C. Note that there is no control over the shared Trotter term, which save the quantum resources. For Hamiltonians with certain symmetries, there is no need to use ancilla and controlled unitaries. Figure 1C2 shows the quantum circuit implementation for measuring the sampled instance involved in $\langle \vec{j}|O|\vec{i}\rangle$. In the ancilla-free measurement scheme, in Fig. 1C2, two types of initial states (prepared by *U_p_*) are involved. The unitary operator *U_p_* prepares either $|\psi_{0}\rangle =U_{p}|0^{⊗n}\rangle$ or $\frac{1}{\sqrt{2}}\left(|\psi_{\text{Ref}}\rangle +|\psi_{0}\rangle \right)=U_{p}|0^{⊗n}\rangle$, where $|\psi_{\text{Ref}}\rangle$ belongs to a different symmetry sector from $|\psi_{0}\rangle$ and is orthogonal to the initial state (see Materials and Methods for details).

To realize the ancilla-free measurement, it is necessary to pair the terms in *S* and *V* such that each resulting term preserves the symmetry instead of breaking it individually. The issue arises when the Trotter remainder *V* is expanded in the Pauli basis: Applying each individual Pauli term generally breaks the symmetry. To address this issue, we decompose the Hamiltonian into the basis of swap and tensor products of Pauli-*Z* operators, rather than Pauli bases. Let us give an example of the 1D Fermi-Hubbard model, which, after the Jordan-Wigner Transformation, takes the form of $H=J_{1}\sum_{i}\left(X_{i}X_{i+1}+Y_{i}Y_{i+1}\right)+J_{2}\sum_{i}Z_{i}Z_{i+1}+h_{z}\sum_{i}Z_{i}$. This can be reformulated as $H=4J_{1}\sum_{i}{\text{SWAP}}_{i,i+1}+\left(J_{2}-J_{1}\right)\sum_{i}Z_{i}Z_{i+1}+h_{z}\sum_{i}Z_{i}$ where the identity term in the Hamiltonian is trivial, and will always be removed. Each individual term in the Hamiltonian is unitary and commutes with the particle number operator. The only difference lies in the compensation terms, which involve the ${\text{SWAP}}_{i,i+1}$ operators, Pauli-*Z* operators, and their corresponding exponentiated forms. When restricted to a linear NN architecture, the circuit depth within a segment is d=O(1) and is shown to be advantageous over other methods.

For electronic problems, the Hamiltonian can also be reformulated with swap and Pauli-*Z* operators. The kinetic term under the Jordan-Wigner transformation, for example, ${\hat{T}}_{\mathrm{ij}}=h_{\mathrm{ij}}\left({\hat{a}}_{i}^{†}{\hat{a}}_{j}+{\hat{a}}_{j}^{†}{\hat{a}}_{i}\right)=\frac{h_{\mathrm{ij}}}{2}\left(X_{i}X_{j}{⊗}_{k=i+1}^{j-1}Z_{k}+Y_{i}Y_{j}{⊗}_{k=i+1}^{j-1}Z_{k}\right)$ can be reformulated as

$${\hat{T}}_{\mathrm{ij}}=h_{\mathrm{ij}}\left(2{\text{SWAP}}_{i,j}{⊗}_{k=i+1}^{j-1}Z_{k}-\frac{1}{2}{⊗}_{k=i}^{j}Z_{k}\right)$$

The potential term ${\hat{V}}_{\text{ijkl}}$ can be similarly reformulated such that each individual term commutes with the particle number operator. Although the Hamiltonian is reformulated in the basis of swap and Pauli-*Z* operators, the implementation of the Trotter formulae remains the same as that in the Pauli basis. The depth complexity for the electronic structure problem in Eq. 7 is shown to be d=O(n) (see Materials and Methods).

### Asymptotic gate and depth complexity

Our results with randomized composite LCU can nearly match and, in some cases, outperform the previous best methods for gate complexity with respect to Δ, ε, λ, and *n*, although the sample complexity becomes worse in η. The gate complexity for estimating generic Hamiltonian’s eigenstate properties is summarized as follows.

Theorem 1. **[Gate complexity for general Hamiltonians (informal)] *Observable estimation*** *(**Problem 1**): To achieve the error of observable’s expectation on the eigenstate* $|u_{j}\rangle$ *within* ε *with success probability* 1−ϑ*, the gate complexity in a single circuit is* $O\left({\left(\Delta^{-1}\text{ln}\left(\varepsilon^{-1}\right)\right)}^{1+\frac{1}{4k+1}}\right)$ *provided* $N_{s}=O\left(\varepsilon^{-2}∥O{∥}_{1}^{2}\text{ln}\left(1/\vartheta \right)\right)$ *samples.*

***Eigenenergy estimation*** *(**Problem 2**): To achieve the eigenenergy estimation error within* κ*, the total gate complexity is* $O\left(\kappa^{-\left(1+\frac{1}{4k+1}\right)}\text{ln}\left(1/\vartheta \right)\right)$ *with success probability* 1−ϑ*, approaching to the Heisenberg limit. Alternatively, by using the methods proposed in* (*20*) *and* Algorithm 1*, the maximum gate complexity in a single circuit can be reduced to* $O\left({\left(\Delta^{-1}\text{ln}\left(\kappa^{-1}\right)\right)}^{1+\frac{1}{4k+1}}\right)$ *at the cost of more samples.*

Here, the spectral filter in Eq. 3 is constructed using the *2k*th-order Trotter formula as a building block in realizing the real-time dynamics in Fig. 1. Note that the spectral filter can be constructed with *k* = 0 (i.e., without the Trotter term *S*) and its gate complexity is also covered by Theorem 1. Our zeroth-order design with *k* = 0 is similar to that of (*17*), although our sampling procedure is simpler and it shows advantages in depth when qubit connectivity is restricted.

As shown in Table 1, our method can achieve polylogarithmic dependence on inverse precision, outperforming the QPE-based method and matching the result by QSP. As a variant of QSP, QETU can achieve near-optimal ground state preparation by querying real-time evolution. However, these types of methods intrinsically hinge on a coherent implementation of *e*^–*iHt*^, which rules out any random sampling method. Therefore, it is not straightforward to achieve polylogarithmic dependence on inverse precision by these coherent methods. As η only appears in sample complexity instead of the gate or depth complexity which is more of a concern in NISQ or early FTQC applications, its dependence is not included in Table 1. A more detailed description of both the gate and sample complexity can be found in Theorem 3 in section S1. The actual cnot and non-Clifford gate counts considering circuit synthesis are shown in Proposition 3 in Materials and Methods.

Next, we discuss the depth complexity for various physical Hamiltonians. An advantage of our method over QSP-based methods is that the commutator relations can be used (when *k* ≥ 1), such that it could achieve a better system size dependence. For Heisenberg models, the gate complexity of our method is $O\left(n^{1+o\left(1\right)}\right)$. In contrast, the solution given by QSP (*28*) is $O\left(n^{2}\text{log}\varepsilon^{-1}\right)$. On the other hand, the implementation of time evolution by Trotter methods will undermine the optimal scaling with respect to λ, Δ, and ε. Because controlled operations are necessary in QETU (even for their control-free approach), it becomes suboptimal when qubit connectivity is restricted. To summarize, our method has the following features: (i) It has better scaling with respect to λ, Δ, and ε, although η dependence is worse than the other advanced methods. (ii) For the Heisenberg model, we shall see that our method based on the *2k*th-order Trotter formula has depth $O\left(n^{\frac{2}{4k+1}}\varepsilon^{-\frac{1}{4k+1}}\right)$, which has better system-size *n* and precision ε scaling over existing strategies. It may be worth noting that one may simultaneously achieve near optimal scaling in both the size and precision as $O\left(n^{\frac{2}{4k+1}}\text{log}\varepsilon^{-1}\right)$ if the higher-order commutators in the Trotter error remainder could be compensated. The result for depth complexity is summarized in Theorem 2. A comparison with other methods is shown in the third column of Table 1.

Theorem 2. **[Gate and depth complexity for lattice Hamiltonians (informal)]** *The gate complexity for estimating an n-qubit Heisenberg Hamiltonian’s eigenstate property is* $O\left(n^{1+\frac{2}{4k+1}}\right)$*, with circuit depth* $O\left(n^{\frac{2}{4k+1}}\right)$*.*

The proof idea for Theorem 1 and Theorem 2 is illustrated in Fig. 2. See the formal version of the theorems and the proof in section S3.

The result can be extended to the simulation of molecules. For electronic problems, the second-quantized electronic-structure Hamiltonian in the plane-wave dual basis has the form [see (*50*, *52*, *59*)]

$$H=\hat{T}+\hat{V}=\sum_{\mathrm{pq}}T_{\mathrm{pq}}{\hat{a}}_{p}^{†}a_{q}+\sum_{p}U_{p}{\hat{n}}_{p}+\sum_{p\neq q}V_{\mathrm{pq}}{\hat{n}}_{p}{\hat{n}}_{q}$$ (7)

where $\hat{T}$ and $\hat{V}$ represent the kinetic and potential terms of the fermionic Hamiltonian, respectively; $\hat{a}$ and ${\hat{a}}^{†}$ are fermionic creation and annihilation operators; and ${\hat{n}}_{p}$ is the number operator for the corresponding spin-orbital and the total number of terms $L=O\left(n^{2}\right)$. To estimate the eigenstate property of the Hamiltonian described by Eq. 7, the circuit depth scales as $O\left(n^{2+\frac{2}{4k+1}}\right)$. When restricted to NN architecture, the circuit depth by QETU scales as $\Omega \left(n^{3+\frac{1}{2k}}\right)$. More general second-quantized quantum chemistry problems with $L=O\left(n^{4}\right)$ terms will be discussed in Materials and Methods.

**Fig. 3.**
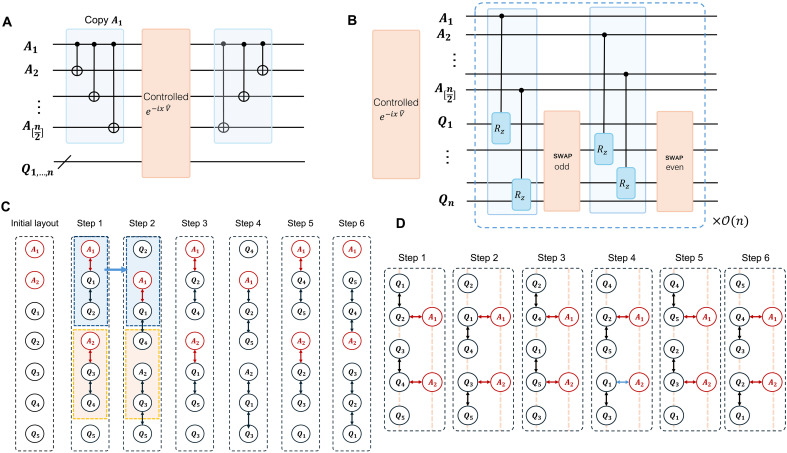
The implementation of the controlled exponentiation of potential terms Ctrl-$e^{-\mathrm{ix}\hat{V}}$ on an NN architecture (1D or 2D) using $\left[\frac{n}{2}\right]$ ancillas with depth O(*n*). (**A**) Copy the classical information on the qubit *A*_1_ to *A*_2_, …, *A*_[*n*/2]_ and then undo the copy operation. This circuit is equivalent to using *A*_1_ as the single-controlled qubit to control all the other physical qubits *Q*_1_ to *Q_n_*. *Q_i_*: *i*th physical qubit (encoding the *n*th spin-orbital). *A*_1_: *i*th ancilla. The copy operation allows all controlled rotations [blue-shaded box in (B)] to be implemented using NN gates. (**B**) The circuit block for controlled $e^{-\mathrm{ix}\hat{V}}$, using NN operations $e^{-{\mathrm{iZ}}_{i}Z_{i+1}}$ followed by [n/2] swap operations detailed in (C). The circuit block in (B) is repeated O(n) times. An example is shown in (C). cnot operations are omitted in the sub-figure. (**C**) 1D linear architecture. The ancillas $A_{i}$ (i=1,2,…,[n/2]) and physical qubits *Q_i_* (i=1,2,…,n) can be placed in the way shown in Step 1 in O(n) depth. The red arrow connecting *A_i_* and *Q_j_* is used to represent to perform the controlled-$R_{z}$ rotation, sandwiched by cnot operations on adjacent qubits *Q_j_* and *Q_k_* (connected and illustrated by the black arrow), which realizes $e^{-{\mathrm{iZ}}_{j}Z_{k}}$. The black arrow connecting *Q_j_* and *Q_k_* is used to represent performing the corresponding cnot operations in realizing $e^{-{\mathrm{iZ}}_{j}Z_{k}}$, then followed by a swap operation. The transformation from step 1 (the blue- and orange-shaded boxes) to step 2 can be realized by two swap gates (cyclic swap operation). The rest of the transformation is realized in the same way. (**D**) 2D planar architecture. The qubit connectivity is represented by the orange dashed line.

As a by-product, we show that the controlled *e*^–*i*θ*H*^ can be implemented with a linear-depth circuit d=O(n) comparable to the control-free simulation of electronic problems in (*62*, *63*). The result is formalized in Proposition 4 with a graphical proof in Fig. 3. It is directly applicable to a range of quantum algorithms for applications in quantum chemistry, materials, and lattice gauge theories, which require controlled unitaries as subroutines.

### Quantum resource estimation

Here, we compare the resource costs associated with different algorithms listed in Table 1, focusing on the maximum cnot gates and T gate counts in each circuit for various physical systems, including lattice models and quantum chemistry problems. The QPE method relies on Hamiltonian simulation, which can be realized by the Trotterization or qubitized QW, with the latter having a better ε scaling at a cost of a larger overhead. We find that the fourth-order (random) Trotter result performs the best among all the Trotter methods, which is consistent with the result in (*58*). The quantum circuit is synthesized to cnot gates, single-qubit Clifford gates, and non-Clifford gates (including single-qubit *Z*-axis rotation *R_z_* gates and T gates). The elementary operations in the block encoding of *H* used in QSP are the select and prepare operations (*64*). The cost for these two operations is shown in detail in section S5, which serves as the basis for analyzing the resource requirements of algorithms that query the block encoding of *H*.

We consider the Heisenberg Hamiltonian

$$H=\sum_{i=1}^{n-1}\left(J_{x}X_{i}X_{i+1}+J_{y}Y_{i}Y_{i+1}+J_{z}Z_{i}Z_{i+1}\right)+H_{f}$$ (8)

where the external field is applied $H_{f}=h_{x}\sum_{i=1}^{n}X_{i}+H_{b}$ with additional field *H_b_* acting on the boundary. The periodic boundary condition is imposed. When *h_x_* = 0 when an additional field $H_{b}=\sqrt{c^{2}-1}\left(Z_{1}-Z_{n}\right)$ is applied, the ferromagnetic Heisenberg Hamiltonian with negative couplings *J_i_* has a constant gap Δ(c)=4(c−1) in the infinite size limit [see (*65*)]. Here, we consider a more challenging regime with the antiferromagnetic types of couplings with $J_{x}=J_{y}=1$ and $J_{z}=2J_{x}$ and field *h_x_* = 0.25, in which more excited states will emerge and is usually more interesting. Nevertheless, even in this case, we find by numerical fitting that, for *n* ≤ 100, the energy gap is not very small. The gap can be fitted by a polynomial function with leading order $\Delta =O\left(n^{-\frac{1}{2}}\right)$, which agrees quite well with the actual gap at small system sizes, while an exponential decaying function does not agree well, see section S8 for the fitting results. We set the initial state overlap as a constant, as similarly used in (*12*, *17*, *21*, *49*), whose dependence is analyzed in Theorem 5 in section S3.

**Fig. 4.**
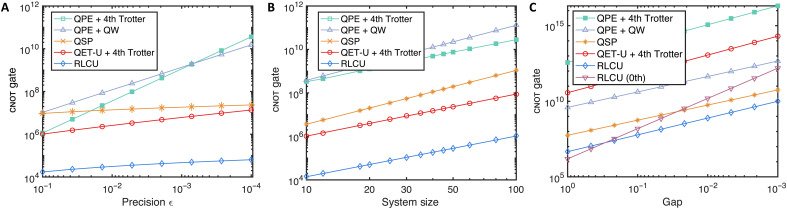
Gate count estimates for the eigenstate property estimation tasks for the Heisenberg Hamiltonian and P450 molecule. (**A**) Gate count comparison with different target precision for 20-site Heisenberg Hamiltonian. (**B**) Gate count comparison with increasing system size to achieve precision of 0.001. The energy gaps are determined through numerical fitting, which agrees well with the results obtained from exact diagonalization. (**C**) Gate count for the P450 with A-type active space as a function of the energy gap, which is treated as an independent variable to analyze its effect. The pairing orders with both *k* = 0 and 1 are shown in (C).

**Fig. 5.**
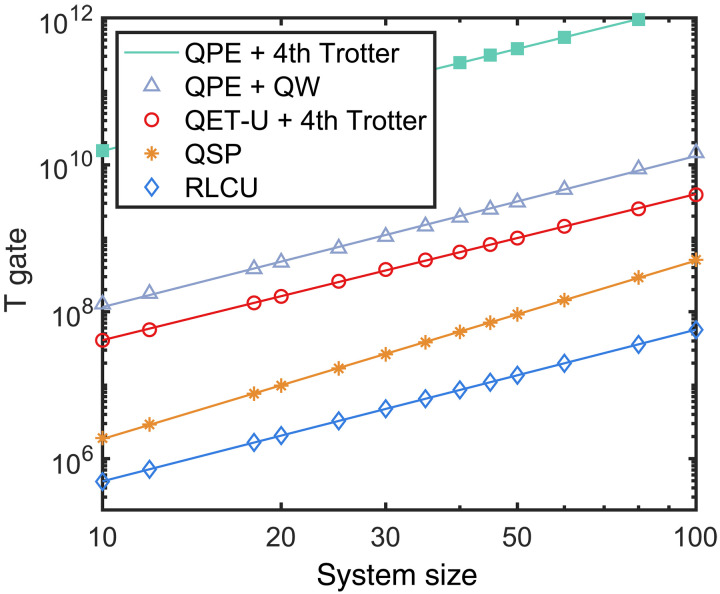
The scaling of the T gate count with system size for the Heisenberg model. The RLCU method involves at most 10^4^ single-qubit Pauli rotation *R_z_* gates and 10^6^ T gates for 20-qubit Heisenberg model. The circuit synthesis method is detailed in section S5.

First, we present the gate number estimates for the above Heisenberg model with different target precisions, as shown in Fig. 4A, which validates the high-precision feature of our method. Next, we show the gate count dependence on the system size. Figure 4B shows that our method has a better system size dependence than QSP, both in the gate count and in the asymptotic scaling. The T gate count scaling for the Heisenberg model has a similar behavior and is shown in Fig. 5. The large reduction compared to QSP arises from exploiting the commutation relations of Hamiltonian terms. Note that our method only requires rotation gates *R_z_*(θ) with identical and small angles, and, thus, further reductions for T gates can be achieved by using the protocols in (*66*, *67*).

**Fig. 6.**
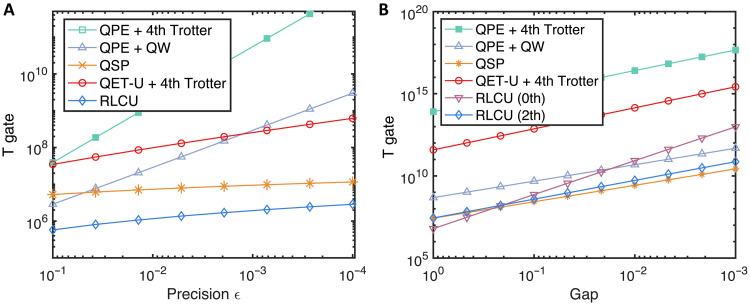
The T gate count under the same setup of Fig. 4. (**A**) The T gate count dependence on precision ε for the Heisenberg model. (**B**) The T gate count dependence on the energy gap for the P450 molecule. As noted in the main text, the simulation of fermionic dynamics with Trotterization is not optimized in this work. The total weight wt(*H*) = 591.

We further discuss the resource cost for the cytochrome enzyme (P450) molecule with the A-type active space specified in (*46*), as shown in Fig. 4C. The methods for compiling the single- and double-excitation operators are used to reduce gate overhead, which is more efficient than naively Trotterizing each fermionic term. However, the cost for Trotterization in every step is not optimized, in which each term is nonlocal and thus contributes substantially to the total gate count. The methods for simulating fermionic Hamiltonians can be directly incorporated (*50*, *59*, *68*). Moreover, our result reflects the gate count in the worst-case scenario. The commutation relations among Hamiltonian terms can be naturally exploited to further reduce the computational cost of our method. We leave the improvements about leveraging the properties of molecular Hamiltonians to future work. The T gate counts for the Heisenberg model, and P450 molecules are presented in Fig. 6.

### Experimental implementation on IBM devices

**Fig. 7.**
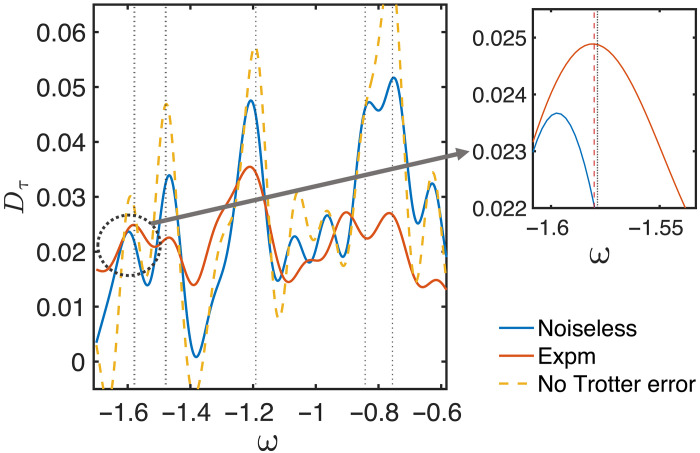
Implementation of the RLCU algorithm on IBM quantum devices. We consider searching the ground state energy of a 12-qubit normalized anisotropic Heisenberg Hamiltonian (Eq. 8) without any external field. We present the ideal result with finite τ and finite cutoff but without any Trotterization error, represented by the orange dotted lines. We also show the results obtained using the noiseless Trotterized quantum circuit and the noisy experimental (Expm) data, denoted by the blue and red lines, respectively. For different lines, *D*_τ_(ω) is computed classically using data points obtained from different setups, including both numerical simulations and experimental measurements. The right panel is a zoom-in of a smaller range of ground-state energy estimates shown in the left panel. The red dotted line represents the experimentally estimated ground-state energy, which is extremely close to the ideal value shown by the black dotted line, with an error of 0.001. The energy estimation error for the excited state is about 0.005.

**Fig. 8.**
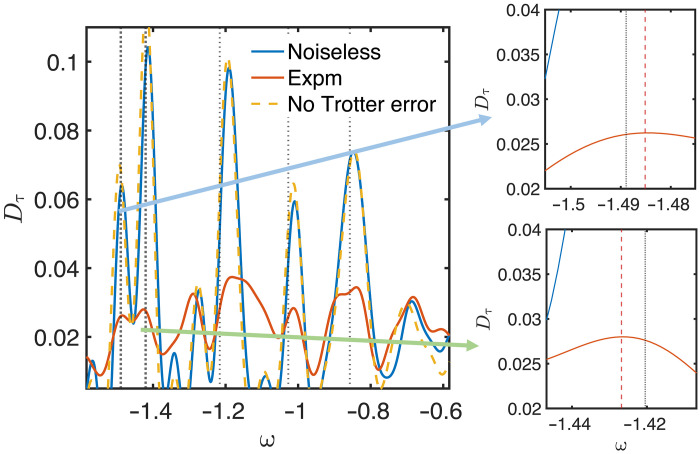
Searching ground state and first excited state energies of the XXZ Heisenberg Hamiltonian on IBM quantum devices. The parameters of the Hamiltonian are *J_x_* = *J_y_* = 0.8, *J_z_* = 1, and *h_z_* = 0.2 in Eq. 8. The initial state is chosen as a Neel state. The reference state is prepared with only one additional cnot gate compared to the original state. The imaginary time and the cutoff are set as τ = 4 and *x_c_* = 2. The figure on the right provides a zoomed-in view of a narrower energy range, highlighting the estimated ground- and first-excited energies shown in the left panel with errors of 0.003 and 0.006, respectively.

We focus on estimating the low-lying eigenenergies of normalized 12-site anisotropic Heisenberg Hamiltonians, especially the ground-state energy and the first excited-state energy. The simulations are performed on the IBM processor with the ancilla-free measurement scheme (see Fig. 1C2). The reference state is set to be orthogonal to the initial antiferromagnetic state. The eigenenergy estimation algorithm is illustrated in Fig. 1B2, where the search by *D*_τ_(ω) over ω (based on experimental measurements) only requires classical computation, indicated by the red line in Fig. 7. The experimental estimation of the ground-state energy achieves high accuracy, with an error of about 0.001 (0.01*J* with Heisenberg coupling *J*), as shown in the zoom-in region in Fig. 7. Then, we consider the antiferromagnetic XXZ model and present the search for its low-lying eigenenergies in Fig. 8. The experiment involved circuits with up to 2000 cnot gates and 20,000 single-qubit gates with a relatively long evolution time. The maximum energy error remains within 0.005 for both eigenvalues. The high precision is attained without relying on error mitigation techniques owing to the intrinsic algorithmic noise robustness.

## DISCUSSION

We have provided a full-stack quantum algorithm based on randomized composite LCU for estimating the eigenstate property and eigenenergy of many-body systems. While previous works have mostly considered query complexity, our approach can achieve near-optimal precision and system size scaling at the quantum circuit level, even with NN connectivity. We present an ancilla-free strategy by choosing the elementary operators to be symmetry-conserved operators (e.g., swap and Pauli-*Z* operators), which is particularly important for implementation on quantum devices, as the restriction of connectivity will incur a large overhead when compiling it into local operations. Moreover, we show a concrete gate count analysis for various physical models concerning the circuit synthesis, which shows remarkable improvements in both asymptotic scaling and actual gate counts. Our work presents concrete resource estimates for lattice and molecular systems, providing guidance for the computation of physical problems on real devices. We would note that it is precisely through adopting this bottom-up quantum circuit design methodology that we can demonstrate deterministic energy estimation in experiments, which achieves higher accuracy and deeper circuit execution (*69*–*71*) without relying on variational ansatz.

Similar to other spectral filter methods, the random-sampling–based method cannot overcome the fundamental limitations set up by the initial state and the energy gap. Lee *et al.* (*5*) numerically investigated its scaling for several many-body examples, which quickly decreases with increasing system size, aligning with the complexity conjecture. Nonetheless, even when provided a sufficiently good initial state, preparing the ground state of two-local Hamiltonians remains as BQP hard (known as the guided local Hamiltonian problem) (*72*, *73*). Therefore, it does not rule out the possibility of quantum speedup. In addition, similar to other random-sampling spectral filter methods (*21*, *24*), the sample complexity with respect to η is less optimal than that achieved by QSP with amplitude amplification, which, nonetheless, also introduces a large circuit compilation cost. On one hand, our algorithm is compatible with initial state preparation methods such as adiabatic evolution and variational methods. On the other hand, the random sampling method offers nontrivial advantages. In particular, when considering qubit connectivity, the depth complexity is independent of η and nearly independent of the system size, which is not achievable by protocols relying on coherent implementations. Therefore, our approach could be useful for estimating the requirement for early quantum usefulness, where the primary constraint lies in gate count and circuit depth.

This work also provides a user-friendly toolbox for researchers to analyze the individual costs for elementary units in quantum simulation and thus enables comparison across different eigenstate algorithms with various initial conditions. Our framework (decomposing the task into the elementary operations), along with the toolbox for analyzing the cost for each elementary operation, is readily useful as a building block for end-to-end resource estimations for a broad class of quantum algorithms. We also note that recent works consider preparing the ground state by simulation of an open system dynamics described by Lindbladians or a thermal process (*34*), which may partially overcome the limitations set by the initial state (*5*) although the simulation time (*33*, *74*) is longer than the spectral filter method by numerics. As the fundamental building block of these methods (*32*–*34*, *74*) is querying the real-time evolution, the actual resource cost can be analyzed similarly with our approach.

## MATERIALS AND METHODS

This section is centered around Fig. 2, which shows the connections of different subsections in Materials and Methods and illustrates the proof idea of Theorem 1 and Theorem 2. In the “Composite LCU for the spectral filter” section, we show how to construct the composite LCU formula as outlined in Fig. 1 and Algorithm 1. The hierarchy of different components in the composite LCU is shown in Fig. 2. Then, we will show the methods for eigenenergy estimation and eigenstate property estimation and provide the circuit depth and gate complexity for the two tasks. Specifically, the “Observable estimation” section shows how errors in observable expectations can be bounded given the approximation error of the composite LCU formula. The “Estimation by quantum circuits: general strategy and ancilla-free strategy” section shows the circuit realization of the composite LCU form by presenting the explicit form of each circuit instance. An ancilla-free composite LCU formula and measurement scheme are presented for symmetry-conserved cases. The “Analysis of circuit depth and gate complexity” section analyzes the depth and gate complexity for general and Hamiltonian-specific cases.

### Composite LCU for the spectral filter

Now, we show how to construct the composite LCU form of the spectral filter. We mainly consider the Gaussian filter $g_{\tau }\left(H\right)=g\left(\tau H\right)$ but present the construction of Hermitian *g* in a general form following the convention in (*22*). Therefore, the results can be readily applied to the investigation of other functions, such as a product of Lorentz and Gaussian functions (*25*) and the Gaussian derivative function in (*20*). To address the eigenstate tasks, we consider the spectral filter g(τ(H−ω)) with ω = *E_j_*, which can be expressed as $g\left(\tau \left(H-\omega \right)\right)=\sum_{i=0}^{2^{n}-1}g\left(\tau \left(E_{i}-\omega \right)\right)|u_{i}\rangle \langle u_{i}|$.

For an input state $|\psi_{0}\rangle =\sum_{i}c_{i}|u_{i}\rangle$, the state after applying the spectral filter g(τ(H−ω)) at a finite τ is given by

$$|\psi \left(\tau \right)\rangle =\frac{g\left(\tau \left(H-\omega \right)\right)|\psi_{0}\rangle }{∥g\left(\tau \left(H-\omega \right)\right)|\psi_{0}\rangle ∥}=\frac{\sum_{i}g\left(\tau \left(E_{i}-\omega \right)\right)c_{i}|u_{i}\rangle }{\sum_{i}{|c_{i}|}^{2}g{\left(\tau \left(E_{i}-\omega \right)\right)}^{2}}$$ (9)

It is easy to see that the Gaussian function $g\left(\tau \left(E_{i}-\omega \right)\right)$ decreases exponentially with τ and *E_i_* − ω. When taking ω = *E_j_*, the amplitudes of the normalized state ∣ψ(τ)〉 concentrate to the eigenstate with energy *E_j_*, and the evolved state asymptotically approximates the eigenstate $|u_{j}\rangle$ with nonzero $|\langle \psi_{0}{|u_{j}\rangle |}^{2}\neq 0$ for sufficiently large τ as ${\text{lim}}_{\tau \to \infty }g\left(\tau \left(H-E_{j}\right)\right)|\psi_{0}\rangle \propto |u_{j}\rangle$.

There are two important parameters τ and ω in the spectral filter *g*(·), in which τ is an imaginary time scaling factor and ω = *E_j_* indicates a shift of the original function. We shall see that τ indicates the timescale for the spectral filtering procedure, and larger τ will cool the state closer to the target eigenstate. The shift ω plays an important role in searching the eigenenergies (see Fig. 1B) and in the eigenstate property estimation. From the above equation, readers may wonder whether we can still get the concentration around the eigenstate $|u_{j}\rangle$ if we do not know the value of *E_j_* a priori. Here, we can find that ω only appears in classical postprocessing and will not be involved in quantum measurement. Therefore, we will classically tune ω as a variable to find the true eigenenergy without increasing any quantum resource cost.

The Gaussian filter can be decomposed into the basis of real-time evolution as

$$g\left(\tau \left(H-\omega \right)\right)=c\int_{-\infty }^{\infty }\mathrm{dp}\left(x\right)e^{\mathrm{ix}\left(\omega -H\right)}$$ (10)

with *p*(*x*) being a Gaussian distribution. The quantum state given by Eq. 9 becomes

$$|\psi \left(\tau \right)\rangle =c\int_{-\infty }^{\infty }\mathrm{dp}\left(x\right)e^{i\tau x\omega }|\phi \left(x\tau \right)\rangle$$

which is now a superposition of real-time evolved states $|\phi \left(x\tau \right)\rangle =e^{-\mathrm{ix}\tau H}|\psi_{0}\rangle$ with probability distribution dp(x)=p(x)dx.

Instead of preparing the above quantum state directly, we focus on the goal of obtaining arbitrary observable expectation values. Specifically, we aim to measure any observable *O* of the evolved state ∣ψ(τ)〉, i.e.,

$${\langle O\rangle }_{\psi \left(\tau \right)}=\langle \psi \left(\tau \right)|O|\psi \left(\tau \right)\rangle =\frac{N_{\tau }\left(O\right)}{D_{\tau }\left(\omega \right)}$$ (11)

where $D_{\tau }\left(\omega \right)=\langle \psi_{0}|g^{2}\left(\tau \left(H-\omega \right)\right)|\psi_{0}\rangle$ and $N_{\tau }\left(O\right)=\langle \psi_{0}|g\left(\tau \left(H-\omega \right)\right)\mathrm{Og}\left(\tau \left(H-\omega \right)\right)|\psi_{0}\rangle$. Hereafter, we have omitted ω in *N*_τ_ for simplicity.

The denominator is $D_{\tau }\left(\omega \right)=c^{2}\int_{-\infty }^{\infty }d\tilde{p}\left(x\right)e^{i\tau x\omega }\langle \psi_{0}|e^{-i\tau \mathrm{xH}}|\psi_{0}\rangle$ with $d\tilde{p}\left(x\right)=\frac{1}{2}\mathrm{dx}\int_{-\infty }^{\infty }p\left(\frac{z+x}{2}\right)p\left(\frac{z-x}{2}\right)\mathrm{dz}$. The expectation values of the denominator and the numerator can be expressed as $v=\text{Tr}\left({\mathrm{Og}}_{\tau }\left(H-\omega \right)\rho_{0}g_{\tau }\left(H-\omega \right)\right)$ with $\rho_{0}=|\psi_{0}\rangle \langle \psi_{0}|$, and the denominator is obtained by taking *O* = *I*.

For the numerator *N*_τ_(*O*), we can efficiently obtain it by sampling the distribution $\mathrm{dp}\left(x,x^{\prime }\right)=\mathrm{dp}\left(x\right)\mathrm{dp}\left(x^{\prime }\right)$ and then estimating the mean value $E_{x,x^{\prime }}\langle \phi \left(x^{\prime }\tau \right)|O|\phi \left(x\tau \right)\rangle$, where each term can be measured by the Hadamard test circuit or using the ancilla-free measurement strategy detailed in the “Estimation by quantum circuits: general strategy and ancilla-free strategy” section. We can similarly obtain the denominator *D*_τ_ by estimating $E_{x}\langle \psi_{0}|e^{-\mathrm{ix}\tau H}|\psi_{0}\rangle$ with probability $d\tilde{p}\left(x\right)$.

The unnormalized eigenstate can be effectively realized by applying a spectral filter $g_{\tau \to \infty }\left(H-E_{j}\right)$ to an initial state. When τ→∞, the expectation value of an observable on the eigenstate satisfies

$$\langle O\rangle =\frac{N_{\tau \to \infty }\left(O\right)}{D_{\tau \to \infty }}$$

Here, it is also easy to check that the denominator is nonvanishing, given by $D_{\tau \to \infty }\left(E_{j}\right)=\eta$ under the assumption. Thus, we arrive at the ideal observable expectation.

To realize the spectral filter with unitary operations, we avoid the infinite time length by truncating the time in real-time evolution to a given threshold. The observable when considering a finite τ is estimated by ${\langle O\rangle }_{\tau }=N_{\tau }\left(O\right)/D_{\tau }$. Below, we discuss the LCU form of *g* when considering the cutoff. The truncated Gaussian filter takes an explicit composite LCU form of

$$g_{\tau }\left(H-\omega \right)=c\int_{-x_{c}}^{+x_{c}}\mathrm{dxp}\left(x\right)e^{\mathrm{ix}\tau \omega }e^{-i\tau \mathrm{xH}}$$ (12)

with the integral region $\left[-x_{c},x_{c}\right]$. The integrand is a real-time evolution with time length τ*x*.

Let us consider implementing $e^{-i\tau \mathrm{xH}}$ with more elementary gates as illustrated in Fig. 1A. Specifically, consider the following LCU decomposition

$$e^{-i\tau \mathrm{xH}}=\mu \left(x\tau \right)\sum_{\vec{r}\in K_{x}}\text{Pr}\left(\vec{r},x\tau ,\nu \left(x\tau \right)\right)U_{\vec{r}}$$ (13)

where $\vec{r}$ specifies the unitary $U_{\vec{r}}$ involved in the LCU formula of $e^{-i\tau \mathrm{xH}}$, $U_{\vec{r}}$ is a unitary operator, and $\text{Pr}\left(\vec{r},x\tau ,\nu \left(x\tau \right)\right)$ represents the normalized decomposition coefficients of $U_{\vec{r}}$. Then, we have

$$\begin{array}{c} g_{\tau }\left(H-\omega \right)\\ =c\left(\mu \right)\int_{-x_{c}}^{+x_{c}}{\mathrm{dxp}}_{\mu }\left(x\right)e^{\mathrm{ix}\tau \omega }\sum_{\vec{r}\in K_{x}}\text{Pr}\left(\vec{r},x\tau ,\nu \left(x\tau \right)\right)U_{\vec{r}} \end{array}$$ (14)

where we have defined the normalization factor $c\left(\mu \right)≔c\int_{-x_{c}}^{+x_{c}}p\left(x\right)\mu \left(x\tau \right)\mathrm{dx}$ and $p_{\mu }\left(x\right)=p\left(x\right)\mu \left(x\tau \right)/c\left(\mu \right)$, see Proposition 5 in section S2 for the detailed derivation.

Then, we show how to find the operator $U_{\vec{r}}$ and the corresponding probability distribution, with which the LCU decomposition in Eq. 13 can be determined. The overall idea is to divide the time evolution into ν segments, and, for each segment, we realize both the Trotter evolution and the Trotter error, as proposed in (*57*). For evolution time *t*, let us denote the real-time evolution $U\left(t\right)≔e^{-\mathrm{iHt}}=U_{m}^{\nu }$ with time interval m=t/ν. We choose to implement the unitary $U\left(m\right)=e^{-\mathrm{imH}}$ by a deterministic *2k*th-order Trotter formula *S*_2*k*_ and the Trotter remainder *V*_2*k*_, which gives us $U\left(m\right)=V_{2k}\left(m\right)S_{2k}\left(m\right).$ As shown in Fig. 1A3, the LCU formula of *V*_2*k*_ can be expressed as

$${\tilde{V}}_{2k}\left(m\right)=\mu \left(m\right)\sum_{r}\text{Pr}\left(r,m,\nu \left(m\right)\right)W_{r}$$ (15)

where *W_r_* is a unitary operator with the error of the formula ε_2*k*_(*m*). The overall LCU formula of *U*(*t*) is a product of each individual formula

$$U\left(t\right)={\left(U\left(m\right)\right)}^{\nu }=\mu \left(t\right)\sum_{\vec{r}\in K_{t}}\text{Pr}\left(\vec{r},t,\nu \left(t\right)\right)U_{\vec{r}}$$ (16)

where $\vec{r}≔\left(r_{1},r_{2},\ldots ,r_{\nu }\right)$ with each $U_{r_{i}}$ being sampled from the distribution of Pr(r,m,ν(m)) introduced in Eq. 15, and we denote $\text{Pr}\left(\vec{r},t,\nu \left(t\right)\right)≔\prod_{i\leq \nu }\text{Pr}\left(r_{i},m,\nu \left(m\right)\right)$ and $U_{\vec{r}}≔\prod_{i\leq \nu }W_{r_{i}}S_{2k}$. With some derivation, one can prove that μ = μ(*t*)^ν^ and $\varepsilon_{2k}\left(t\right)\leq \nu \mu \left(m\right)\varepsilon_{2k}\left(m\right)$. Equation 16 shows how $U_{\vec{r}}$ can be sampled and thus how *g* can be realized. This is illustrated in Fig. 1A3; in the figure, the index by *i* is related to the probability Pr(*i*) when decomposing *g* into real-time evolution *U*(*t_i_*).

Using BCH formula, the *2k*th-order remainder $V_{2k}\left(m\right)≔U\left(m\right)S_{2k}{\left(m\right)}^{†}$ takes an explicit form of $V_{2k}\left(m\right)=\text{exp}\left(i\sum_{s=1}^{\infty }E_{2k,s}m^{s}\right)$ with Hermitian operators *E*_2*k*,*s*_. Because the *2k*th-order Trotter error is compensated, we know that *E*_2*k*,*s*_ *=* 0 for *s* ≤ 2*k*. Expanding *V*_2*k*_(*m*), we have $V_{2k}\left(m\right)=\sum_{s=0}^{\infty }F_{2k,s}\left(m\right)$, where we group the terms by the order of *m*, and *F*_2*k*,*s*_ denotes the *s*-order expansion term of *V*_2*k*_(*m*) associated with *m^s^*.

Below, we briefly discuss the error due to truncation by *s_c_*. The truncation error is found to have a quick decrease with an increasing truncation order *s_c_* (*57*). Using the fact of $F_{2k,s\leq 2k}\left(m\right)=0$, we can rewrite $V_{2k}^{\left(s\right)}\left(m\right)$ as $V_{2k}^{\left(s\right)}\left(m\right)=I+\sum_{s=2k+1}^{s_{c}}F_{2k,s}\left(m\right).$ Given the truncation, the LCU formula for *U*(*m*) is

$$U_{2k}^{\left(s_{c}\right)}\left(m\right)=V_{2k,s}\left(m\right)S_{2k}\left(m\right)$$ (17)

which consists of a deterministic second-order Trotter formula and the Trotter error compensation term. The overall LCU formula for *U*(*t*) is to repeat the sampling of ${\tilde{U}}_{2k}\left(m\right)$ for ν times, $U_{2k}^{\left(s_{c}\right)}\left(t\right)={\left(U_{2k}^{\left(s_{c}\right)}\left(m\right)\right)}^{\nu }$.

Equation 14 is a composite LCU formula; more specifically, it is a (c(μ),0) LCU formula. When we consider a finite $x_{c},s_{c}$, it will introduce algorithmic errors. When considering a finite *s_c_*, the spectral filter becomes

$$g_{\tau ,x_{c},s_{c}}\left(H-\omega \right)=c\int_{-x_{c}}^{x_{c}}\mathrm{dxp}\left(x\right)e^{\mathrm{ix}\tau \omega }U_{2k}^{\left(s_{c}\right)}\left(x\tau ,\nu \left(x\tau \right)\right)$$ (18)

It is easy to check that $∥g_{\tau ,x_{c},s_{c}}\left(H-\omega \right)∥\leq c\left(1+\varepsilon_{s_{c}}\right)$. The operator distance between $g_{\tau ,x_{c}}$ and $g_{\tau ,x_{c},s_{c}}$ due to finite *s_c_* is

$$∥g_{\tau ,x_{c}}-g_{\tau ,x_{c},s_{c}}∥\leq c\int_{-x_{c}}^{x_{c}}\mathrm{dxp}\left(x\right)e^{\mathrm{ix}\tau \omega }∥U\left(x\tau \right)-U_{2k}^{\left(s_{c}\right)}\left(x\tau ,\nu \left(x\tau \right)\right)∥\leq \varepsilon_{s_{c}}$$ (19)

when $∥U-U_{2k}^{\left(s_{c}\right)}\left(x\tau ,\nu \left(x\tau \right)\right)∥\leq \varepsilon_{s_{c}}/c$. To achieve an additive error of the approximation $∥\left.U\left(t\right)-U_{2k}^{\left(s_{c}\right)}\left(t\right)\right)∥\leq \varepsilon_{s_{c}}$, Lemma 1 in section S3B gives the required segment numbers ν.

Based on the above result, the total error of the spectral filter function *g* can be bounded by the sum of the individual error terms. The derivation process is illustrated within the orange box of Fig. 2. Specifically, we can bound the error in spectral filter construction by comparing the operator distance between $∥g_{\tau ,x_{c},s_{c}}-g_{\tau ,x_{c},s_{c}\to \infty }∥$, which gives an upper bound for the numerator and the denominator. Using the triangular inequality, the operator error between *g*_τ_ and $g_{\tau ,x_{c},s_{c}}$ defined in Eq. 18 is

$$∥g_{\tau }-g_{\tau ,x_{c},s_{c}}∥\leq \varepsilon_{x_{c}}+\varepsilon_{s_{c}}$$ (20)

We find that the segment number of the order

$$\nu =O\left({\left(\lambda \tau x_{c}\right)}^{1+\frac{1}{4k+1}}\right)$$ (21)

suffices to ensure the LCU construction error up to ε. The detailed segment number for eigenstate property estimation along with the actual overhead is provided in Theorem 5 in section S3. The required segment number for eigenenergy estimation is shown in Theorem 7 in section S4. Together with Proposition 3 in the “Analysis of circuit depth and gate complexity” section, one proves Theorem 1.

For lattice models, the segment number can be further reduced to $\nu =O\left(n^{\frac{2}{4k+1}}{\left(\tau x_{c}\right)}^{1+\frac{1}{4k+1}}\varepsilon^{-\frac{1}{4k+1}}\right)$. This result leads directly to the proof of Theorem 2.

### Observable estimation

The previous section focused on error propagation in the LCU formulation. Here, given the constructed LCU formula, we analyze the estimation of eigenstate properties and the associated error. We first show how to measure the expectation value of the observable *O*. Denote the initial state on which the spectral operator acts as ρ. The expectation value of the observable *O* on the normalized state can be expressed by

$$\langle O\rangle =\frac{N\left(O\right)}{D}=\frac{\text{Tr}\left(g\rho g^{†}O\right)}{\text{Tr}\left(g\rho g^{†}\right)}$$

The numerator can be expressed by

$$N\left(O\right)=\mu^{2}\sum_{\mathrm{ij}}\text{Pr}\left(i\right)\text{Pr}\left(j\right)e^{i\left(t_{i}-t_{j}\right)\omega }\text{Tr}\left(U\left(t_{i}\right)\rho U^{†}\left(t_{j}\right)O\right)$$ (22)

Denote the expectation value of the estimator of the numerator over measurement outcomes with *U*(*t_i_*) and *U*(*t_j_*), $\text{Tr}\left(U\left(t_{i}\right)\rho U{\left(t_{j}\right)}^{†}O\right)$, as ${\bar{N}}_{\mathrm{ij}}\left(O\right)$. The expectation value of *N*(*O*) is given by sampling over the distribution

$$\bar{N}\left(O\right)=\mu^{2}E_{\mathrm{ij}}{\bar{N}}_{\mathrm{ij}}\left(O\right)$$ (23)

When considering finite gate complexity and sample complexity, the eigenstate property is estimated by

$${\hat{O}}_{\tau ,x_{c},s_{c}}=\frac{{\hat{N}}_{\tau ,x_{c},s_{c}}\left(O\right)}{{\hat{D}}_{\tau ,x_{c},s_{c}}}$$ (24)

The selection of $\tau ,x_{c},s_{c}$ can be determined by analyzing the error of ${\hat{O}}_{\tau ,x_{c},s_{c}}$ compared to the ideal value, which can be analyzed using the operator distance detailed in the above section. Proposition 2 shows the error in observable estimation when we are given a (μ, ε) random sampling formula of *g* by $\tilde{g}$ in Eq. 1.

Proposition 2 **(Observable estimation using the composite LCU formula)** *The estimation error of* $N_{g}\left(O\right)≔\text{Tr}\left(g\rho g^{†}O\right)$ *is bounded by* $\varepsilon_{N}≔|{\hat{N}}_{\tilde{g}}\left(O\right)-N_{g}\left(O\right)|\leq ∥O∥\left(2\mu^{2}\varepsilon +\varepsilon_{n}\right),$ *with* $N_{s}=\mu^{4}\text{ln}\left(2/\vartheta \right)/\varepsilon_{n}^{2}$ *samples and a success probability* 1−ϑ*. The error for the denominator is bounded by* $\varepsilon_{D}≔|{\hat{D}}_{\tilde{g}}-D_{g}|\leq 2\mu^{2}\varepsilon +\varepsilon_{n}$*. The error of observable expectation* $\varepsilon_{O}$ *is bounded by* $\varepsilon_{O}\leq D_{g}^{-1}\left(\left(O+1\right)\varepsilon_{D}+\varepsilon_{N}\right)$*.*

Given Proposition 2, the error of the observables can be bounded given the RLCU form. The proof is shown in section S2 (where the formal version is presented). Using these results of error propagation in the LCU form, we can determine the required segment number ν*_c_* to achieve the desired accuracy as shown in Eq. 21. Theorem 5 in section S3 and Theorem 7 in section S4 present the required segment number ν*_c_* and the gate count (with the actual prefactors included) for achieving the desired accuracy for the two problems. They can be used to estimate the actual gate numbers needed for physical Hamiltonians.

The total gate count is determined by multiplying ν*_c_* and the gate count for realizing *S*_2*k*_ and *V*_2*k*_ within each segment, denoted by *g* hereafter (with an abuse of notation). The gate count for lattice models is straightforward g=O(n). For electronic structure problem in Eq. 7, a naive realization of *S*_2*k*_ and *V*_2*k*_ will result in the cost $g=O\left(n^{3}\right)$. However, we could bring down the scaling by considering the properties of fermions. The kinetic operator is quadratic and thus can be diagonalized by an efficient circuit transformation, either by Givens rotations or fermionic fast Fourier transform (*50*, *62*, *63*). By using the results in (*75*), $e^{-\mathrm{im}\hat{T}}$ and $e^{-\mathrm{im}\hat{V}}$ can be implemented with O(nlogn) gates, with *m* being the time length. The compensation term will cost O(n) gates. Therefore, we have g=O(nlogn), and the total gate count for electronic structure problem is $g_{\text{tot}}=\tilde{O}\left(n{\left(\lambda \Delta^{-1}\right)}^{1+\frac{1}{4k+1}}\text{log}\varepsilon^{-1}\right)$.

### Estimation by quantum circuits: General strategy and ancilla-free strategy

In this section, we discuss the circuit realization for Algorithm 1. As discussed in the main text, to measure $\langle \vec{j}|O|\vec{i}\rangle$ (introduced in Eq. 6), we can use the Hadamard test circuit with an extra ancillary qubit. For Hamiltonians with certain symmetries, we do not need this ancillary qubit and thus do not require any controlled operation. Below, we first discuss the general cases by introducing an ancillary qubit. Then, we discuss the ancilla-free composite LCU formula and the corresponding measurement schemes.

#### General cases

There are two extreme cases: case I, *t_i_* = 0 (or *t_j_* = 0); and case II, *t_i_* = *t_j_*. For *t_j_* = 0, each component in Eq. 6 $\langle \vec{j}|O|\vec{i}\rangle$ can be measured using a Hadamard test circuit, which is the circuit depicted in the green box in Fig. 1C.

For $0<t_{j}<t_{i}$, suppose that *U*(*t_j_*) is divided into *m* segments and *U*(*t_i_*) is divided into ν segments with the first *m* segments being set the same as that of *U*(*t_i_*). The quantum circuit that can measure $\langle \vec{j}|O|\vec{i}\rangle$ is shown in Fig. 1. After postselecting ∣+〉 on the ancillary qubit (but before measurements on *B*), the circuit on system *B* outputs

$$\frac{1}{2}\left(\prod_{q=1}^{m}W_{j_{q}}S+\prod_{q=1}^{\nu }W_{i_{q}}S\right)|\psi_{0}\rangle$$

The case of *t_i_* = *t_j_* is a special case in Fig. 1C1 by only implementing the circuits in the green box.

Here, we can use a single-shot measurement strategy to estimate *N*(*O*) in Eq. 22. Given the sequence of unitary operators obtained from sampling, the circuit in Fig. 1C1 can be used to measure $\langle \vec{j}|O|\vec{i}\rangle$. Specifically, we initialize the ancilla in ∣+〉, apply the controlled-unitaries, and perform the measurement on the *X* basis, with the measurement outcome recorded as a={0,1}. Similarly, we repeat the process but with an inverted phase gate *S*^†^ applied before measurement, with the measurement outcome b={0,1}.

One can verify that the estimator $\hat{d}={\left(-1\right)}^{a}+i{\left(-1\right)}^{b}$ is unbiased, $E_{a,b}\hat{d}=\langle \vec{j}|O|\vec{i}\rangle$. Now, take an estimator as

$$\hat{v}=c^{2}\left(\mu \right)e^{i\omega \left(t_{i}-t_{j}\right)}\hat{d}$$ (25)

which can be proven to be unbiased

$$E_{t_{i},t_{j},\vec{i},\vec{j}}E_{a,b}\hat{v}=\langle \psi_{0}|g_{\tau }\left(H-\omega \right){\mathrm{Og}}_{\tau }\left(H-\omega \right)\psi_{0}\rangle$$ (26)

By analyzing the error dependence on the finite τ, *x_c_*, and segment number ν in Eq. 21, we arrive at the result of property estimation in Theorem 1 (see detailed description in Theorem 5 and Theorem 7). More detailed proof can be found in sections S3 and S4.

#### Ancilla-free composite LCU formula and the measurements

In the above section, given a sampled configuration $\left(t_{i},t_{j},\vec{i},\vec{j}\right)$, the real and imaginary part of $\langle \vec{j}|O|\vec{i}\rangle$ can be obtained by the circuit in Fig. 1C. In cases of the Heisenberg model and electronic structure problems (Eq. 7), the target problem has certain symmetries S satisfying [H,S]=0. For symmetry-conserved systems with [U,S]=0, it is possible to estimate the expectation value of a unitary $\langle \psi_{0}|U|\psi_{0}\rangle$ without ancilla, as proposed in (*76*, *77*). However, the issue here is that, as the unitary operator is realized by a Trotter-LCU expansion in Eq. 5, at least there exists a Pauli operator $W_{i_{q}}$ that does not commute with S. We first briefly introduce how to measure $\langle \psi_{0}|U|\psi_{0}\rangle$ without ancilla, where the unitary is either $U=e^{-\mathrm{iHt}}$ or $U=e^{-{\mathrm{iHt}}_{1}}\hat{O}e^{-{\mathrm{iHt}}_{2}}$, followed by the design of the unitary in Eq. 5 such that [U,S]=0.

The expectation value $\langle \psi_{0}|U|\psi_{0}\rangle$ can be expressed as $\langle \psi_{0}|U|\psi_{0}\rangle ={\mathrm{re}}^{i\theta }$. If $|\psi_{0}\rangle$ is a product state or can be prepared as $|\psi_{0}\rangle =U_{p}{|0\rangle }^{⊗n}$, then the amplitude of the expectation value $r=|\psi_{0}|U|\psi_{0}|$ can be obtained by measuring $U_{p}^{†}{\mathrm{UU}}_{p}{|0\rangle }^{⊗n}$ in the computational basis. The next step is to obtain the phase θ, for which we make use of the fact that the unitary operation conserves the symmetry of S, [U,S]=0. To do so, a reference state is introduced $|\psi_{\text{Ref}}\rangle$, which lies in a different sector of the initial state, such that $\langle \psi_{\text{Ref}}|U|\psi_{0}\rangle =0$. Here, $\langle \psi_{\text{Ref}}|U|\psi_{\text{Ref}}\rangle$ can be computed classically.

In addition, the following state can be prepared

$$|\phi_{0}\rangle =U_{p}|\psi_{0}\rangle =\frac{1}{\sqrt{2}}\left(|\psi_{\text{Ref}}\rangle +|\psi_{0}\rangle \right)$$

As $r_{s}=\left|\langle \psi_{0}|U_{p}^{†}{\mathrm{UU}}_{p}|\psi_{0}\rangle \right|$ can be measured on a computational basis, the phase θ can be computed.

Note that there will be double solutions for determining θ in general and may be hard to distinguish. One may track the dynamics of expectation values to determine the correct phase.

The preparation of the superposition state is simple, requiring only one additional cnot gate compared to the original state, which is used for real demonstrations on IBM devices. Discussions can be found in section S3. Now, to measure $\langle \vec{j}|O|\vec{i}\rangle$, we use the circuit in Fig. 1C to generate

$$U_{p}^{†}\left(\prod_{q=1}^{m}W_{i_{q}}^{†}S^{†}\right)O\left(\prod_{q=1}^{\nu }W_{i_{q}}S\right)U_{p}|\psi_{0}\rangle$$

and then measure on a computational basis.

Recall that our algorithm consists of implementing the Trotter formula *S* and the Trotter remainder *V*. For the implementation of *S*, we need to pair the terms to ensure each individual term does not break the symmetry. Take the quantum chemistry problems, $H=\hat{T}+\hat{V}$, for example. We group each term ${\hat{T}}_{\mathrm{ij}}=h_{\mathrm{ij}}\left({\hat{a}}_{i}^{†}{\hat{a}}_{j}+{\hat{a}}_{j}^{†}{\hat{a}}_{i}\right)$ in the kinetic term $\hat{T}$, which conserve the particle number. Its exponentiation $e^{-i\theta {\hat{T}}_{\mathrm{ij}}}$ can be directly implemented, which is similar to the implementation for the Heisenberg model case. For the potential term $\hat{V}$, we can group its component $g_{\text{ijkl}}\left({\hat{a}}_{i}^{†}{\hat{a}}_{j}^{†}{\hat{a}}_{k}{\hat{a}}_{l}+{\hat{a}}_{l}^{†}{\hat{a}}_{k}^{†}{\hat{a}}_{j}{\hat{a}}_{i}\right)$ and implement its exponentiation similarly.

Note that operators are typically indicated by using the hat notation. In this work, to avoid confusion with estimators, we reserve the hat notation exclusively for fermionic operators and estimators. Here, the symbol *V* refers to the Trotter remainder. The potential term in molecular Hamiltonians is denoted as $\hat{V}$ and is distinguished from the Trotter remainder by the hat notation.

The issue is that, if we expand the Trotter remainder into Pauli bases, then applying each individual Pauli term will break the symmetry. One may think of expanding *V*(*m*) into $V\left(m\right)=\sum_{k,l,i,j}{\hat{T}}_{i}^{k}{\hat{V}}_{j}^{l}m^{k+l}$, where ${\hat{T}}_{i}$ and ${\hat{V}}_{j}$ are components of the kinetic and potential terms, respectively. Although each term conserves the symmetry, the issue is that ${\hat{T}}_{i}$ or ${\hat{V}}_{j}$ is not unitary and cannot be implemented directly. To address this issue, we decompose the Hamiltonian into swap and tensor products of Pauli-*Z* operators rather than Pauli operators.

The explicit formulation for the Fermi-Hubbard model and quantum chemistry problems has been presented in the main text. Although the Hamiltonian is reformulated in the basis of swap and Pauli-*Z* operators, the implementation of the Trotter formulae remains the same as that in the Pauli basis. Only the compensation term will be different from the case with one ancillary qubit. As shown in the “Analysis of circuit depth and gate complexity” section, the ancilla-free measurement strategy shows advantages when qubit connectivity is restricted.

### Analysis of circuit depth and gate complexity

We first discuss the gate complexity for general Hamiltonians. Then, we analyze the circuit depth and gate complexity for various Hamiltonians of physical relevance when using either our method or other early fault-tolerant quantum algorithms. A comparison with a focus on circuit depth for each elementary block is shown in Table 2.

**Table 2.** Circuit depth *d* and gate count *g* in each segment with NN or arbitrary architecture. The ancilla-free or the one-ancilla schemes are compared when using our method or QETU method.

Hamiltonians	NN (ancilla-free)	NN (1-ancilla)	NN (QETU) (*43*)	Arbitrary
Heisenberg model	d=O(1)	d=O(n)	d=O(n)	d=O(1)
	g=O(n)	g=O(n)	g=O(n)	g=O(n)
Electronic structure (Eq. 7)	d=O(n)	$d=\Omega \left(n^{2}\right)$	$d=\Omega \left(n^{2}\right)$	d=O(n)
	$g=O\left(n^{2}\right)$	$g=O\left(n^{3}\right)$	$g=O\left(n^{3}\right)$	g=O(nlogn)
		d=O(n) ([n/2] ancilla)		
Molecular Hamiltonians (Eq. 29)	$d=O\left(n^{2}\right)$	$d=\Omega \left(n^{3}\right)$	$d=\Omega \left(n^{3}\right)$	$d=O\left(n^{2}\right)$
	$g=O\left(n^{3}\right)$	$g=O\left(n^{4}\right)$	$g=O\left(n^{4}\right)$	$g=O\left(n^{2}\text{log}n\right)$

#### Gate complexity for general cases

The maximum resource appears in the case of *t_j_* = 0 in Fig. 1C, represented by the orange box. In this case, we need to implement one controlled Trotter formula plus two controlled compensation terms specified by *V_i_*. The minimum resource appears in the case when *t_j_* = *t_j_*, in which case we need to implement the Trotter formula plus two controlled compensation terms specified by *V_i_*. This is because a controlled Trotter is more costly than a controlled compensation operation, as analyzed in Proposition 3. The resources in other cases with *t_j_* < *t_i_* will be between case I and case II.

Define the following characters of the Hamiltonian, $\text{wt}\left(H\right)≔\sum_{l=1}^{L}\text{wt}\left(P_{l}\right)$, and ${\text{wt}}_{m}\left(H\right)≔{\text{max}}_{l}\text{wt}\left(P_{l}\right)$. Here, wt(*P_l_*) indicates the weight of the Pauli operator *P_l_*, i.e., the number of {X,Y,Z} terms in *P_l_*. The following result gives the required elementary gates.

Proposition 3 **(Elementary gate count)** *Suppose the composite LCU is realized by Algorithm 1 and the Hamiltonian dynamics U = e*^–*iHt*^ *is realized by Eq. 5 with maximum segment number* ν*. The gate complexity of the circuit instance in eigenstate property estimation is the following:* cnot *gate number:* $\nu \left(4\times 5^{k-1}\text{wt}\left(H\right)+4{\text{wt}}_{m}\left(H\right)+2\text{min}\left(n,s_{c}{\text{wt}}_{m}\right)\right)-2L$*, single-qubit Pauli rotation gate number:* (2L+4)ν*.*

We give more details in the following. Overall, the circuit within each segment consists of one controlled Trotter plus controlled compensation terms. Each of the components within the Trotter formula takes the form of *e*^–*iHm*^ with evolution time m=t/ν. The cost by Trotter is given by Lemma 3 in section S5.

A controlled-Pauli-rotation gate, Ctrl-exp(−iPθ/2) with Pauli operator *P*, can be realized by 2(wt(H)−1) cnot gates, a controlled single-qubit *R_z_*(θ) gate with rotation angle θ, and some single-qubit Clifford gates. Furthermore, the controlled *R_z_*(θ) gate can be decomposed into two single-qubit *Z*-axis rotation gates and two cnot gates. Therefore, the cost for the controlled Trotter operator Ctrl-*S*_2*k*_(*m*) in each segment and the number of cnot gates are $2\times 5^{k-1}\left(2\text{wt}\left(H\right)-2L\right)$ and *2L* controlled single-qubit Pauli rotations.

The compensation term *V* consists of a controlled multiqubit Pauli rotation operator and some controlled Pauli operators. The controlled multiqubit Pauli rotation operator can be realized by $2{\text{wt}}_{m}\left(H\right)$ cnot gates and two single-qubit Pauli rotations. The Pauli gates are randomly drawn from ${\left\{P_{l}\right\}}_{l}$ according to the corresponding probability distribution. In the worst case, the gate sequence length is the truncated value *s_c_*, and, thus, the cnot gate number is bounded by $\left(s_{c}{\text{wt}}_{m}\left(H\right)\right)$. Note that saturation occurs when the number of compensation Pauli operations reaches *n*. To sum up, for the compensation term, the number of cnot gates is upper bounded by

$$2{\text{wt}}_{m}\left(H\right)+\text{min}\left(n,s_{c}{\text{wt}}_{m}\right)\leq 3n$$ (27)

and two single-qubit Pauli rotations. As there is a saturation of the gate count for the Trotter-error-compensation indicated by Eq. 27, one can take *s_c_* to be infinity in deriving the asymptotic scaling in gate complexity in Theorem 1 and Theorem 2.

One can thus check that case I is more costly than case II in Fig. 1. For observable dynamics described in Eq. 6, there are two compensation terms, but simply one controlled Trotter Ctrl-*S*_2*k*_, as illustrated in Fig. 1C1. Together with the cost for the Trotter formula, the number of cnot gates is upper bounded by

$$\nu \left(4\times 5^{k}\text{wt}\left(H\right)-2L+4{\text{wt}}_{m}\left(H\right)+2\text{min}\left(n,s_{c}{\text{wt}}_{m}\right)\right)$$ (28)

The number of single-qubit Pauli rotations is

(2L+4) ν

The gate complexity with the ancilla-free strategy can be analyzed in a similar way. Its key advantage is better depth scaling as it allows circuit compilation using only NN gates without overhead. This is discussed in detail in the “Analysis of circuit depth and gate complexity” section.

#### Hamiltonian-specific circuit compilation and depth analysis

The circuit depth and gate count for Problem 1 and Problem 2 using either the ancilla-free scheme and the one-ancilla scheme are displayed in Table 2. Below, we will discuss the result when the qubit connectivity is taken into account.

We consider Hamiltonians including (i) Heisenberg models, (ii) electronic structure in the plane wave dual basis in Eq. 7, and (iii) second-quantized molecular Hamiltonian in the form of $H=\sum_{i,j=1}^{n}h_{\mathrm{ij}}{\hat{a}}_{i}^{†}{\hat{a}}_{j}+\frac{1}{2}\sum_{i,j,k,l,=1}^{n}g_{\text{ijkl}}{\hat{a}}_{i}^{†}{\hat{a}}_{j}^{†}{\hat{a}}_{k}{\hat{a}}_{l}$. A common strategy for efficiently representing the Hamiltonians with fewer terms and low weights (*49*, *53*, *78*, *79*) is to reformulate the two-body fermionic operators as a sum of squared one-body operators by the Cholesky decomposition (*79*), reformulated as

$$H=\hat{K}+\frac{1}{2}\sum_{\ell }^{\Gamma }{\hat{L}}_{\ell }^{2}$$ (29)

where $\hat{K}$ and ${\hat{L}}_{\ell }$ are the one-body terms, the number of terms is Γ=O(n) (*53*), and the constant has been removed.

For general cases, the circuit within each segment consists of two parts: the controlled implementation of the Trotter formula *S* and the Trotter remainder *V*. The only difference is the compensation term, which uses the symmetry-conserved gates as the elementary gates, i.e., swap, Pauli-*Z* gates, and their exponentiations. We observe that, to realize the compensation term, at most, d=O(n) and g=O(n) are required in the worst case, which is fewer than the implementation of the Trotter formula in all physical Hamiltonian cases. Table 2 displays the gate complexity (including both cnot gates and non-Clifford gates) within each segment when taking the qubit connectivity into account. We elaborate on these results below.

We first discuss the Heisenberg-type Hamiltonians. Each component $e^{-i\delta t_{i}\left(J_{1}X_{i}X_{i+1}+J_{1}Y_{i}Y_{i+1}+J_{2}Z_{i}Z_{i+1}\right)}$ with time duration δ*t_i_* can be realized by three cnot gates and three single-qubit Pauli rotation gates. When restricted to a linear NN architecture, $d_{\text{Trotter}}=O\left(1\right)$. In the ancilla-free scenario, the compensation term is ${\text{SWAP}}_{i,i+1}$, Pauli-*Z* operators and the exponentiations, resulting in d=O(1) and g=O(n). In the one-ancilla scenario, because we need to implement controlled two-qubit Pauli rotations, when restricted to linear NN architecture, we need to swap the ancillary qubit sequentially to qubit 1,…,n and then perform the controlled rotation. Then, we need to undo the swap to change back the ordering of the qubits. The compensation term can be done when the ancillary qubit is adjacent to the target controlled qubit. Compared to the ancilla-free scheme, we cannot do it in parallel, resulting in d=O(n) and g=O(n). When removing the restriction of the qubit connectivity, both scenarios have d=O(1) and g=O(n).

For the electronic structure problem in Eq. 7, it can be grouped into $H=\hat{T}+\hat{V}$. Below, we discuss the cost for the ancilla-free scheme and the one-ancilla scheme with a linear NN architecture. The kinetic term is a noninteracting term that can be diagonalized as $\hat{T}=\hat{C}\left(\sum_{i}\alpha_{i}{\hat{n}}_{i}\right){\hat{C}}^{†}$. For the implementation of $e^{-i\theta \hat{T}}$, an NN architecture requires d=O(n) and $g=O\left(n^{2}\right)$. For the interacting potential term $e^{-i\theta \hat{V}}$ that consists of $O\left(n^{2}\right)$ terms, an NN architecture requires d=O(n) and $g=O\left(n^{2}\right)$. Therefore, $d_{\text{Trotter}}=O\left(n\right)$ and $g_{\text{Trotter}}=O\left(n^{2}\right)$. For the compensation term, because there is only one term in the form of ${\text{SWAP}}_{i,j}{⊗}_{k=i+1}^{j-1}Z_{k}$ or ${⊗}_{k=i+1}^{j-1}Z_{k}$ or ${⊗}_{k=i}^{j}Z_{k}$, which can be implemented at most $d_{\text{Remainder}}=O\left(n\right)$ and $g_{\text{Remainder}}=O\left(n\right)$. We note that, for the two-dimensional (2D) Fermi-Hubbard model, which is a special case of Eq. 7, the compensation term can be implemented with $d_{\text{Remainder}}=1$ given a planar NN architecture. To sum up, an NN architecture requires d=O(n) and $g=O\left(n^{2}\right)$.

We then discuss the gate and depth cost when we need a control ancilla (used in both the so-called one-ancilla scheme and QETU). The first observation is that we do not need to implement controlled $\hat{C}$. The rest of the diagonal part is similar to the case of the Heisenberg model, which requires d=O(n) and g=O(n). The implementation of $e^{-i\theta \hat{T}}$ requires d=O(n) and $g=O\left(n^{2}\right)$. However, the implementation of $e^{-i\theta \hat{V}}$ requires $d=\Omega \left(n^{2}\right)$ and $g=\Omega \left(n^{2}\right)$ in an NN architecture. The challenge of realizing the controlled $\hat{V}$ with one control qubit is that, at each time, the control qubit can only control one rotation $R_{z}\left(\theta_{\mathrm{ij}}\right)$ given a pair of (i,j). Therefore, the controlled rotation cannot be realized in parallel. Naively, we could first apply a cyclic swap operation and then realize each individual $R_{z}\left(\theta_{\mathrm{ij}}\right)$. This results in the total depth $d=O\left(n^{3}\right)$ and gate count $g=O\left(n^{3}\right)$. A lower bound on the depth complexity for realizing the controlled $\hat{V}$ may be given by $\Omega \left(n^{2}\right)$. The compensation term requires at most d=O(n) and g=O(n). Therefore, an NN architecture requires $d=\Omega \left(n^{2}\right)$ and $g=O\left(n^{3}\right)$. Note that the estimate for gate complexity with one-ancilla scheme may not be optimal.

The depth can be reduced to O(n) provided [n/2] ancillary qubits. The result is summarized in Proposition 4.

Proposition 4 *When restricted to linear NN qubit connectivity, a controlled exponentiation Ctrl-e*^*i*θ*H*^ *with molecular Hamiltonian H in Eq. 7 can be implemented in depth* O(n) *using* [n/2] *ancillary qubits. The circuit implementation is illustrated in Fig. 3. When restricted to only one ancillary qubit, the circuit depth is* $\Omega \left(n^{2}\right)$*.*

A graphic proof is shown in Fig. 3. Specifically, Fig. 3 (A and B) shows the overall structure of the circuit with O(n) depth. Figure 3A shows how to copy the classical information of *A*_1_ to the rest of ancilla and lastly give it back to *A*_1_. By using the circuit (example) illustrated in Fig. 3C for 1D architecture and Fig. 3D for 2D architecture, the controlled-*H* can be realized with only NN operations.

When considering an arbitrary architecture, the gate count can be further reduced from $O\left(n^{2}\right)$ to O(nlogn) using the results in (*75*). Note that, in (*59*, *62*, *63*), the potential term $\hat{V}$ is a symmetric translationally invariant two-body coupling term, specifically ${\hat{V}}_{\mathrm{pq}}=\sum_{\nu \neq 0}\frac{2\pi }{\Omega k_{\nu }^{2}}\text{cos}\left(k_{\nu }\cdot r_{p-q}\right){\hat{n}}_{p}{\hat{n}}_{q}$ where momentum modes are defined as $k_{\nu }=2\pi \nu /\Omega^{1/d}$, $r_{m}=m{\left(2\Omega /N\right)}^{1/d}$ with momentum difference *m* = *p* − *q*, and the computational cell volume Ω. This reduction from $O\left(n^{2}\right)$ to O(nlogn) uses the translationally invariant property. The exponentiation of $e^{-\mathrm{ix}\hat{T}}$ can be implemented by the fermionic fast Fourier transform (FFFT), $\text{FFFT}e^{-i\sum_{i}t_{i}{\hat{n}}_{i}}{\text{FFFT}}^{†}$, which can be implemented with g=O(nlogn) and d=O(logn). The exponentiation of $e^{-\mathrm{ix}\hat{V}}$ can be implemented with d=O(n) and g=O(nlogn). In addition, the results for molecular Hamiltonians in Eq. 29 are displayed in Table 2, where the dominant cost comes from the potential terms.

Table S3 in section S2 displays the total gate and depth complexity concerning both cnot and T gates, which can be obtained by using the cost within each segment shown in Table 2. We can readily find that our method performs better in circuit depth in all these cases when qubit connectivity is restricted to an NN architecture.

In addition to the cnot gate count estimates in the main text, the corresponding T gate count is shown in Fig. 6. The search for the eigenenergies of the XXZ Heisenberg model is presented in Fig. 8.

## Supplementary Materials

### This PDF file includes:

Sections S1 to S8 Tables S1 to S3 Figs. S1 to S3 References
