# Supplementary material for: High-precision and low-depth quantum algorithm design for eigenstate problems
Source: Sci Adv. 2026 Jan 16;12(3):eaeb1622. doi: 10.1126/sciadv.aeb1622 (PMC12810647; doi:10.1126/sciadv.aeb1622)
Supplement: Supplementary file 1 — Sections S1 to S8 Tables S1 to S3 Figs. S1 to S3 References [file sciadv.aeb1622_sm.pdf]

Science Advances
AAAS

Supplementary Materials for

High-precision and low-depth quantum algorithm design for eigenstate problems

Jinzhao Sun *et al.*

Corresponding author: Jinzhao Sun, jinzhao.sun.phys@gmail.com; Pei Zeng, qubitpei@gmail.com; Tom Gur, tom.gur@cl.cam.ac.uk; M. S. Kim, m.kim@imperial.ac.uk

Sci. Adv. **12**, eaeb1622 (2026)
DOI: 10.1126/sciadv.aeb1622

This PDF file includes:

Sections S1 to S8
Tables S1 to S3
Figs. S1 to S3
References

In Sec. S1, we provide an overview of quantum algorithms for ground state preparation and energy estimation, followed by an overview of the theoretical results in this work. In the remaining supplementary sections, we present the technical details that support the main results of this work. In Sec. S2, we discuss the construction of the spectral filter with composite LCU. We discuss the error in observation estimation given an LCU form. In Sec. S3, we discuss the building blocks of the eigenstate property estimation algorithm outlined in Algorithm 1. We will follow the proof idea illustrated in Fig. 2 and Methods to prove the main theorems for eigenstate property estimation in the main text. In Sec. S4, we prove the result for eigenenergy estimation in the main text. Sec. S5 analyses the circuit compilation cost for block-encoding-based methods. Sec. S6 and Sec. S7 discuss the cost when using QSP and QPE.

S1. COMPARISON WITH OTHER WORKS

A. Overview of quantum algorithms for ground state preparation

In the main text, we have provided an overview of the quantum algorithms for ground state preparation and property estimation. Here, we reviewed the progress in this rapidly growing field. Among these quantum algorithms, spectral filter-based methods offer a rigorous and deterministic solution, with clear assumptions regarding the initial overlap and energy gap, which will be the major focus of this work. In spectral filter methods, QSP has achieved near-optimal query complexity $\mathcal{O}(\Delta^{-1}\log(\varepsilon^{-1}))$ for target precision ε, as one of the state-of-the-art algorithms [28]. While it is favourable in the long term, QSP hinges on querying the block encoding of the Hamiltonian H, which is challenging for noisy intermediate-scale quantum (NISQ) or early fault-tolerant quantum computing (FTQC) applications. In the early FTQC regime [21, 40], where the number of logical qubits is limited, minimising controlled operations and achieving low circuit depth are essential goals.

Considering the feature of early FTQC [21], there has been considerable progress on quantum algorithms for ground-state property estimation [12, 18–26]. In 2021, Lin and Tong proposed a spectral filter algorithm based on random sampling [21], which achieved the Heisenberg limit for ground-state energy estimation. Using similar techniques, Zeng *et al.* [22] and Zhang *et al.* [24] showed that the time complexity can achieve logarithmic in precision $\log(1/\varepsilon)$ for the ground state property estimation problem. Note that Ref. [22] extends to eigenstate property estimation. Wan *et al.* proposed the randomised algorithm for ground energy estimation, which has a relatively worse gap dependence [17]. On the other hand, following the spirit of QSP, QETU was proposed [43] to prepare the ground state, which achieves near-optimal asymptotic scaling but avoids querying the block-encoding of the Hamiltonian by querying time evolution. The above algorithms assume the usage of only one ancillary qubit and achieve good asymptotic sample and time complexity, which are competitive for the application of noisy intermediate-scale quantum and early FTQC. However, these algorithms assume perfect and efficient queries to the time evolution operator e^{-iHt}. It remains unclear whether the good properties of the above early FTQC algorithms can be preserved if we further expand the time evolution operator into elementary gates. For example, if the time evolution operator is implemented using Trotterisation methods, this will eliminate the advantage of logarithmic scaling in precision. On the other hand, if we introduce advanced block-encoding-based methods to realise the time evolution operator, it needs many ancillary qubits and nonlocal controlled gates, which violates the spirit of early FTQC. The system-size dependence of the algorithms is rarely discussed in existing works, as it highly depends on the detailed circuit-level implementation of e^{-iHt} as well as the qubit connectivity of the device. The central objective of this work is to design full-stack algorithms with high-precision and low-depth features towards NISQ and early FTQC applications.

Summary of the main results. We provide end-to-end gate complexity analysis of the eigenstate property and energy estimation task. Our randomised LCU approach achieves nearly logarithmic scaling on inverse precision, with improved scaling on Δ and λ compared to oracle-free methods. We have built up a framework based on randomised composite LCU formulae, which contains several hierarchies, for analysing the gate complexity for the eigenstate problems. The comparison with other methods is shown in Table I with detailed comparison in Table S1 and Table S2. For eigenstate property estimation, we focus on the comparison for the maximum gate complexity per circuit, while in the literature, the gate complexity may refer to the total number of gates required in the whole algorithm. For electronic structure problems specified in Eq. (7) which is usually compared in the existing literature,

the gate complexity scales $\tilde{\mathcal{O}}(n(\lambda\Delta^{-1}\log\varepsilon^{-1})^{1+\frac{1}{4k+1}})$, which is nearly linear in n (excluding the dependence on λ) and logarithmic in inverse precision. We note that compared to [45, 52, 53, 63], this result is for the depth or gate count in a single-run circuit, which has worse sample complexity.

In addition to addressing gate complexity for generic Hamiltonians, the second contribution of our work is the advantages in circuit depth for various physical problems. These include lattice models and second-quantised plane-wave electronic structures with n spin orbitals and $L = \mathcal{O}(n^2)$ terms in the Hamiltonian. Notably, the 2D Fermi-Hubbard model falls within the problem class. In theory, for problems that conserve certain symmetries, we demonstrate the ability to achieve high-precision and low-depth eigenstate property estimation without the need for ancillary qubits.

To accomplish this, we design new Trotter and Trotter-error compensation circuits that maintain the system's symmetries. The random sampling approach can exploit the commutation relation of the target Hamiltonian terms to reduce the gate complexity, outperforming QSP for the 1D lattice model. Moreover, our approach favours a linear nearest-neighbour architecture. When restricted to nearest-neighbour architecture, for the 1D lattice model, the circuit depth scales $d = \mathcal{O}(n^{\frac{2}{4k+1}})$, while the circuit depth in the QETU-Trotter method scales as $d_{\text{QETU}} = \mathcal{O}(n^{1+\frac{1}{2k}})$. For electronic structure problems specified in Eq. (7), when considering the commutation relation and restricting to a nearest-neighbour architecture, the circuit depth is $d = \mathcal{O}(n^{2+\frac{2}{4k+1}})$, while QETU requires $d = \Omega(n^{3+\frac{1}{2k}})$. A side product is that controlled $e^{-i\theta H}$ can be implemented by a linear-depth circuit $d = \mathcal{O}(n)$, summarised in Proposition 4, comparable to the control-free simulation of electronic problems in [62, 63]. This result can be directly useful for other quantum algorithms which require controlled unitaries as a subroutine.

In the existing resource estimation works [44–48], the energy estimation or eigenstate preparation for practical problems, such as second-quantised quantum chemistry problems with $L = \mathcal{O}(n^4)$ terms [52, 53, 78] and condensed-phase electrons [50, 51], is predominantly based on phase estimation. A typical strategy is to encode the eigenspectra of the Hamiltonian in a unitary for phase estimation by the evolution e^{-iHt}, which is synthesised by Trotterisation [50, 51], or a qubitised quantum walk [52, 53] with eigenspectrum proportionally to $e^{\pm i \arccos(H/\lambda)}$, where λ is a parameter related to the norm of the Hamiltonian. However, due to the cost of phase estimation, the circuit depth will inevitably be polynomial in the precision, which is not optimal for eigenstate energy and property estimation. Recent works [17, 31] have also estimated resources for algorithms based on the computation of the cumulative distribution function (CDF) of the spectral measure of a Hamiltonian which was originally proposed in [21].

The third contribution is to present resource estimations with the method developed in this work, which has better asymptotic scaling in precision and system size in theory, and also has a smaller circuit compilation overhead in practice. In the NISQ applications, the major bottleneck is the number of two-qubit gates for the noisy quantum computer. The major overhead for the error-corrected quantum computer is the T gate count [52], which requires more gates to perform error correction than Clifford gates. We provide a systematic comparison with the existing advanced methods, which include QPE combined with Trotter formulae or qubitised quantum walk (as have been used in [46, 52, 53]), QSP [28] and QETU. The CNOT gate cost for a 20-site Heisenberg model is about 3×10^5 while the T gate cost is about 6×10^6.

For different query models, we provide a useful toolbox for researchers to analyse the individual costs for elementary units (like block encoding and controlled real-time evolution), and thus enable comparison across different eigenstate preparation methods with various initial conditions. We hope that our framework (decomposing the task into the elementary operations) and the toolbox for analysing the cost for each elementary operation can be useful as a building block for resource estimations for other quantum algorithms based on querying the block encoding of H or the time evolution. We analyse the actual gate count for typical problems and compare it with the state-of-the-art methods.

B. Problem setup and comparison with existing works

Problem setup. Consider a gapped n-qubit Hamiltonian with a Pauli decomposition $H = \sum_{l=1}^{L} \alpha_l P_l := \lambda \sum_{l=1}^{L} \tilde{\alpha}_l P_l$ where P_l is a Pauli operator, $\lambda := \sum_l |\alpha_l|$, and $\tilde{\alpha} := \alpha_l/\lambda$. The eigenstate property estimation task is to estimate the expectation value of an observable O on the jth eigenstate of H, $|u_j\rangle$. The assumptions are the following. We assume that we have an estimate of E_j, $\hat{E}_j$ with a small estimation error $\kappa := |\hat{E}_j - E_j|$. We assume that we have a good initial state $|\psi_0\rangle$ which has a nonvanishing overlap with the target eigenstate, $\eta := |\langle\psi_0|u_j\rangle|^2 = \Omega(1/\operatorname{poly}(n))$. We assume a nonvanishing energy gap $\Delta := \min(E_{j+1} - E_j, E_j - E_{j-1})$.

Note that in Problem 1, we follow the convention in [22], which has a slight difference in the definition of the initial overlap η compared to Refs. [28, 43].

Below, we provide relatively formal descriptions for Theorem 1 and Theorem 2, respectively.

Theorem 3 (Eigenstate property estimation for generic Hamiltonians (formal version of Theorem 1))**.** *Suppose we use the method in Algorithm 1 and the conditions and assumptions in Problem 1 hold. Observable estimation (Problem 1): To achieve the error of observable's expectation on the eigenstate $|u_j\rangle$ within ε, the gate complexity in a single circuit is $\mathcal{O}\left(5^{k-1}L(\lambda\Delta^{-1}\ln(\eta^{-1}\varepsilon^{-1}))^{1+\frac{1}{4k+1}}\right)$ when the number of samples is $N_s = \mathcal{O}\left(\eta^{-2}\varepsilon^{-2}\|O\|_1^2\ln(1/\vartheta)\right)$ with a success probability at least $1-\vartheta$.*

Eigenenergy estimation (Problem 2): To achieve the eigenenergy estimation error within κ using Algorithm 1, the gate complexity in a single circuit is $\mathcal{O}\left(5^{k-1}L(\lambda\kappa^{-1}\ln(\eta^{-1}))^{1+\frac{1}{4k+1}}\right)$, with number of samples $N_s = \mathcal{O}\left(\eta^{-2}\ln(1/\vartheta)\right)$ (independent of κ) with a success probability at least $1-\vartheta$, approaching to the Heisenberg limit. Alternatively, by using the methods proposed in [20] and Algorithm 1, the gate complex-

ity in a single circuit is $\mathcal{O}\left(5^{k-1}L(\lambda\Delta^{-1}\ln(\eta^{-1}\kappa^{-1}))^{1+\frac{1}{4k+1}}\right)$ *at the cost of more number of samples* $N_s = \mathcal{O}\left(\eta^{-2}\Delta^4\kappa^{-4}(\ln(\kappa^{-2}\eta^{-1}))^2\ln(1/\vartheta))\right)$ *with a success probability at least* $1-\vartheta$.

Theorem 4 (Gate and depth complexity for lattice Hamiltonians)**.** *For n-qubit Heisenberg Hamiltonians, to estimate the observable on the eigenstate with a precision* ε *and a success probability* $1-\vartheta$*, in a single run, the gate complexity is* $\mathcal{O}(n^{1+\frac{2}{4k+1}}\Delta^{-(1+\frac{1}{4k+1})}\varepsilon^{-\frac{1}{4k+1}}\log(\vartheta^{-1}))$*. The circuit depth when compiled on qubits with nearest-neighbour geometry is* $\mathcal{O}(n^{\frac{2}{4k+1}}\Delta^{-(1+\frac{1}{4k+1})}\varepsilon^{-\frac{1}{4k+1}}\log(\vartheta^{-1}))$*.*

More detailed versions with the actual gate overheads are presented in Theorem 5 and Theorem 7, respectively. The eigenenergy is first assumed to be known a priori. This is the case for linear algebra tasks, in which the true solution corresponds to the ground state of a constructed Hamiltonian with the eigenenergy E_0 being exactly zero. The task with an unknown eigenenergy will be discussed in Supplementary Sec. S3 F.

The comparisons with advanced methods for eigenstate property and eigenenergy estimation are displayed in Table S1 and Table S2, respectively. The near-optimal eigenstate property estimation is indeed enabled by our algorithm design. Specifically, our algorithm design does not rely on a coherent implementation of phase estimation, allowing the use of different Hamiltonian simulation strategies.

As the implementation of real-time evolution in Fig. 1(a2) is a subroutine in the eigenstate algorithm, one could employ the recent advances in Hamiltonian simulation algorithms. There are various approaches that can achieve favourable scaling in the system size and other key parameters, e.g. [13, 60, 61, 80].

The reason why the Trotter error compensation method is chosen and its particular suitability are the following: 1) It integrates naturally into the randomised linear-combination-of-unitaries construction; 2) The actual overhead is small - the actual overhead with prefactors are explicitly calculated in Theorem 5 and Theorem 7; 3) In each circuit run, the circuit structure is deterministic, determined by sampling the operations according to a well-defined randomisation procedure. For the interleaved forward and backward time-evolutions [80], they have shown the scaling advantage but the drawback is that the actual gate count may be even worse than that of either standard Trotter or QSP in small sizes.

In addition, in an ongoing project, we expect that the Trotter error compensation may indeed simultaneously achieve optimal scaling in the system size and precision for k-local Hamiltonians. Therefore we expect that the gate complexity in Theorem 2 may be improved to $\mathcal{O}(n^{1+\frac{2}{4k+1}}\log(\varepsilon^{-1}))$

It may be worth noting here about the advantages of the above Point 3. For each sampled time t, we do not need to change the step-size as in [61] (in which the stepsize $\{s_i\}_{i=1}^m$ needs to be changed for m times). This has a two-fold advantage (1) it does not incur additional measurement overhead could bring bias and fluctuations to the estimated result). (2) in theory single-shot measurement is sufficient (i.e. we do not require the expectation value of $\langle O(t)\rangle$). On the other hand, extrapolation-based methods typically require additional measurements at multiple evolution times, which increases the variance of observable estimation and also may be more susceptible to measurement noise.

As noted in the main text, the spectral filter can be constructed with the Trotter expansion order $k = 0$ (i.e., without the Trotter term S). The corresponding gate complexity is covered in both tables. Our zeroth-order design with $k = 0$ is similar to that of [17], though our sampling process is simpler, as we only need to sample the first-order terms. In contrast, Ref. [17] employs full-order pairing and needs to sample from higher-order terms. Another difference is that our zeroth-order design may be advantages when qubit connectivity is restricted.

To summarise, our work provides a full-stack solution, from high-level query-based design down to end-to-end algorithmic design. This shifts in perspectives which in our view is an appropriate approach for bridging near-term capabilities and long-term goals of fault-tolerant quantum computing. It is precisely through adopting this bottom-up methodology that we are able to achieve the near-optimality at the gate level, and are able to demonstrate the deterministic eigenstate solution on current quantum devices.

S2. CONSTRUCTION OF THE SPECTRAL FILTER

A. Composite LCU formulae for decomposing a nonunitary operator

In the main text, we have introduced a few tools to analyse the property of the composite form of an LCU formula. Proposition 1 shows how to bound the error of a composite LCU formula written in the discretised form. We provide the proof here.

Proof. (of Proposition 1)

Methods	Gate complexity	Depth complexity (lattice models)	Extra qubits
QPE + Trotter (2kth-order)	$\mathcal{O}(L\tilde{\Delta}^{-(1+\frac{1}{2k})}\varepsilon^{-(1+\frac{1}{2k})})$	$\mathcal{O}(n^{1+\frac{1}{2k}}\Delta^{-(1+\frac{1}{2k})}\varepsilon^{-(1+\frac{1}{2k})})$	$\log(\varepsilon^{-1})+\log(\Delta^{-1})$
QPE + QW [53]	$\tilde{\mathcal{O}}(L\tilde{\Delta}^{-1}\varepsilon^{-1})$	$\mathcal{O}(n^2\Delta^{-1}\varepsilon^{-1})$	$\log(L)+\log(\varepsilon^{-1})+\log(\Delta^{-1})$
QSP [28]	$\mathcal{O}(L\tilde{\Delta}^{-1}\log\varepsilon^{-1})$	$\mathcal{O}(n^2\Delta^{-1}\log\varepsilon^{-1})$	$\log(L)+\log(\varepsilon^{-1})+\log(\Delta^{-1})$
QETU [43]	$\tilde{\mathcal{O}}(L\tilde{\Delta}^{-(1+\frac{1}{2k})}\varepsilon^{-\frac{1}{2k}})$	$\mathcal{O}(n^{1+\frac{1}{2k}}\Delta^{-(1+\frac{1}{2k})}\varepsilon^{-\frac{1}{2k}})$	1
This work (2kth-order)	$\tilde{\mathcal{O}}(L\tilde{\Delta}^{-(1+\frac{1}{4k+1})}\log\varepsilon^{-1}))$	$\tilde{\mathcal{O}}(n^{\frac{2}{4k+1}}\Delta^{-(1+\frac{1}{4k+1})}\varepsilon^{-\frac{1}{4k+1}})$	0 or 1
(zeroth-order)	$\mathcal{O}(\tilde{\Delta}^{-2}\log^2\varepsilon^{-1})$	$\mathcal{O}(n^2\Delta^{-2}\log^2\varepsilon^{-1})$	0 or 1

TABLE S1. Comparison of observable estimation on the eigenstate of a Hamiltonian (Problem 1). Here we compare the gate complexity of the algorithm in a single coherent run. The results in the second column in the table are based on Algorithm 1 and Theorem 1 using the 2kth-order Trotter error compensation. Here, $\tilde{\Delta} := \lambda/\Delta$. The results in the third column in the table are based on Theorem 2 when the 2kth-order Trotter formula is used. The eigenenergy is assumed to be known a priori and the unknown eigenenergy case is analysed in Sec. S3 F. The dependence on η is not included in the table since it only appears in sample complexity for our method. Similar to other random-sampling spectral filter methods (see [21, 24]), the sample complexity with respect to η is $\mathcal{O}(\eta^{-2})$ for which the optimal scaling is $\mathcal{O}(\eta^{-1/2})$ achieved by QSP and QETU with amplitude amplification. As noted in the main text, one may simultaneously achieve near optimal scaling in both the size and precision as $\mathcal{O}(n^{\frac{2}{4k+1}}\log\varepsilon^{-1})$ if the higher-order commutators in the Trotter error remainder could be compensated.

Methods	Gate complexity	Depth complexity (lattice models)	Extra qubits
QPE + Trotter (2kth-order)	$\mathcal{O}(L\lambda^{1+\frac{1}{2k}}\varepsilon^{-(1+\frac{1}{2k})})$	$\mathcal{O}(n^{1+\frac{1}{2k}}\varepsilon^{-(1+\frac{1}{2k})})$	$\log(\varepsilon^{-1})+\log(\Delta^{-1})$
QPE + QW [53]	$\tilde{\mathcal{O}}(L\lambda\varepsilon^{-1})$	$\tilde{\mathcal{O}}(n^2\varepsilon^{-1})$	$\log(L)+\log(\varepsilon^{-1})+\log(\Delta^{-1})$
QSP [28]	$\tilde{\mathcal{O}}(L\lambda\varepsilon^{-1})$	$\tilde{\mathcal{O}}(n^2\varepsilon^{-1})$	$\log(L)+\log(\varepsilon^{-1})+\log(\Delta^{-1})$
QETU [43]	$\tilde{\mathcal{O}}(L\lambda^{1+\frac{1}{2k}}\varepsilon^{-(1+\frac{1}{2k})})$	$\tilde{\mathcal{O}}(n^{1+\frac{1}{2k}}\varepsilon^{-(1+\frac{1}{2k})})$	1
This work (2kth-order)	$\tilde{\mathcal{O}}(L\lambda^{1+\frac{1}{4k+1}}\varepsilon^{-(1+\frac{1}{4k+1})})$	$\tilde{\mathcal{O}}(n^{\frac{2}{4k+1}}\varepsilon^{-(1+\frac{1}{4k+1})})$	0 or 1
(zeroth-order) and [17]	$\tilde{\mathcal{O}}(\lambda^2\varepsilon^{-2})$	$\tilde{\mathcal{O}}(n^2\varepsilon^{-2})$	0 or 1

TABLE S2. Total gate complexity in eigenenergy estimation up to estimation error ε (Problem 2). Note that this table presents the total gate complexity, which includes the sample complexity. The dependence on η is not included in the table, as similarly discussed in Table I.

Hamiltonians	Ancilla-free method (NN)	QETU (NN) [43]	arbitrary
1D lattice models	$d=\mathcal{O}(\mathbf{n}^{\mathbf{\frac{2}{4k+1}}}\Delta^{-(1+\frac{1}{4k+1})}\varepsilon^{-\frac{1}{4k+1}})$	$d=\mathcal{O}(n^{1+\frac{1}{2k}}\Delta^{-(1+\frac{1}{2k})}\varepsilon^{-\frac{1}{2k}})$	$d=\mathcal{O}(n^{\frac{2}{4k+1}}\Delta^{-(1+\frac{1}{4k+1})}\varepsilon^{-\frac{1}{4k+1}})$
	$g=\mathcal{O}(n^{1+\frac{2}{4k+1}}\Delta^{-(1+\frac{1}{4k+1})}\varepsilon^{-\frac{1}{4k+1}})$	$g=\mathcal{O}(n^{1+\frac{1}{2k}}\Delta^{-(1+\frac{1}{2k})}\varepsilon^{-\frac{1}{2k}})$	$g=\mathcal{O}(n^{1+\frac{2}{4k+1}}\Delta^{-(1+\frac{1}{4k+1})}\varepsilon^{-\frac{1}{4k+1}})$
Electronic structure	$d=\mathcal{O}(\mathbf{n}^{\mathbf{2}+\mathbf{\frac{2}{4k+1}}}\Delta^{-(1+\frac{1}{4k+1})}\varepsilon^{-\frac{1}{4k+1}})$	$d=\Omega(n^{3+\frac{1}{2k}}\Delta^{-(1+\frac{1}{2k})}\varepsilon^{-\frac{1}{2k}})$	$d=\mathcal{O}(n^{2+\frac{2}{4k+1}}\Delta^{-(1+\frac{1}{4k+1})}\varepsilon^{-\frac{1}{4k+1}})$
(Eq. (7))	$g=\mathcal{O}(n^{3+\frac{2}{4k+1}}\Delta^{-(1+\frac{1}{4k+1})}\varepsilon^{-\frac{1}{4k+1}})$	$g=\mathcal{O}(n^{4+\frac{1}{2k}}\Delta^{-(1+\frac{1}{2k})}\varepsilon^{-\frac{1}{2k}})$	$g=\tilde{\mathcal{O}}(n^{2+\frac{2}{4k+1}}\Delta^{-(1+\frac{1}{4k+1})}\varepsilon^{-\frac{1}{4k+1}})$

TABLE S3. Gate complexity with respect to the energy gap Δ, target precision ε, and system size n for different Hamiltonians' eigenstate property estimation. In the second and third columns, the qubit connectivity is restricted to a linear nearest-neighbour (NN) architecture. The fourth column is the result of our method when there is no restriction on qubit connectivity. In this table, the commutation relation of the Hamiltonian terms is used to improve the system-size scaling. It is worth mentioning that without restriction on connectivity, the gate complexity of electronic structure problems studied in this table can be $g = \mathcal{O}(n(\lambda\Delta^{-1}\log\varepsilon^{-1})^{1+\frac{1}{4k+1}})$ which is logarithmic in inverse precision. The circuit depth results for different physical Hamiltonians are displayed in Table S3. The gate-complexity estimate for electronic Hamiltonians using QETU may not be optimal. QSP and methods based on full-order pairing [17] have worse system-size scaling, and thus they are not included in Table S3.

Recall that the LCU formula of g can be written as

$$g_2 = \mu_1 \sum_i \Pr(i)(U(t_i/\nu))^{\nu}. \tag{S30}$$

Given the LCU formula of $U(t_i/\nu)$, g_2 can be written as

$$g_2 = \mu_1\mu_2^{\nu}\sum_i \Pr(i) \left(\sum_{i_\ell}\Pr(i_\ell)P_{i_\ell}\right)^{\nu} = \mu_1\mu_2^{\nu}\sum_i \Pr(i)\sum_{\{i_\ell\}}\prod_{q=1}^{\nu}\Pr(i_{\ell_q})\prod_{q=1}^{\nu}P_{i_{\ell_q}} \tag{S31}$$

with some abuse of notation.

Given a (μ_2, ε_2)-LCU form of $U(\delta t)$, one can prove that the product of LCU formula $U(\delta t)^\nu$ has a normalisation factor $\mu_2' = \mu_2^\nu$ and an error $\varepsilon_2' \leq \nu\mu_2'\varepsilon_2$. We can prove the result by using the triangle inequality

$$\|g_2 - g\| \leq \|g_1 - g\| + \|g_2 - g_1\| \leq \varepsilon_1 + \nu\mu_1\mu_2^\nu\varepsilon_2,$$

which completes the proof. □

To implement LCU in practice, we could consider either a discretised form or a continuous form. As shown in the main text, the Fourier transform gives an explicit form for decomposing the spectral filter into unitary operators in a continuous form. When the integral form of a spectral filter has a well-defined probability distribution, it can be well-characterised by Eq. (1). Therefore, we use the continuous form for the Gaussian spectral filter. We provide a discretised version of the spectral filter in Sec. S3 G and show that the discretisation error for the Gaussian spectral filter can be sufficiently small.

We provide the composite LCU formula in a continuous form for completeness.

Proposition 5 (Composite LCU formula in a continuous form)**.** *Suppose g_1 is a (μ_1, ε_1)-LCU formula of g,*

$$g_1 = \mu_1 \int dx\, p(x) U(x) \tag{S32}$$

Suppose that each of the summand $U(x)$ has a (μ_2, ε_2)-LCU formula,

$$\tilde{U}(x) = \mu_2(x) \int dy\, q(x, y) V(x, y) \tag{S33}$$

Then the formula

$$g_2 = \mu_1 \int dx\, p(x) \tilde{U}(x) \tag{S34}$$

is a (μ, ε)-LCU formula of g, with $\mu := \mu_1 \int_{-\infty}^{\infty} p(x)\mu_2(x) dx$, and $\varepsilon = \varepsilon_1 + \mu_1\varepsilon_2$.

Proof. (of Proposition 5)

The formula of g_2 can be written as

$$g_2 = \mu_1 \int dx p(x)\mu_2(x) \int dy q(x, y) V(x, y) \tag{S35}$$

We define

$$p_\mu(x) = \mu_1\mu_2(x)p(x)/\mu \tag{S36}$$

with $\mu := \mu_1 \int_{-\infty}^{\infty} p(x)\mu_2(x)dx$. Then g_2 takes the form of

$$g_2 = \mu \int dx p_\mu(x) \int dy q(x, y) V(x, y). \tag{S37}$$

The proof is straightforward by using the triangle inequality $\|g_2 - g\| \leq \|g_1 - g\| + \|g_2 - g_1\| \leq \varepsilon_1 + \mu_1\varepsilon_2$. Note that $p_\mu(x)$ and $q(x, y)$ (for given x) are both normalised and can thus be regarded as probability distributions. Therefore, g_2 can be realised in a random-sampling way by sampling from the distribution $p_\mu(x)$ and then $q(x, y)$.

□

Eq. (S37) is a general (μ, ε) composite LCU form of g. As we shall see in the later discussion, to reduce the maximum evolution time (related to x) we usually set a truncation of x in the integral, i.e.,

$$g_1 = \mu_1 \int_{-x_c}^{x_c} dx p(x) U(x) \tag{S38}$$

with $\tilde{\varepsilon}_1 = \varepsilon_1 + \varepsilon_c$ and ε_c being a small truncation error. Then we may set the constant μ_2 to be $\mu_2 = \max_x \mu_2(x)$ in the LCU formula of $U(x)$, $\tilde{U}(x) = \mu_2 \int dy\, q(x, y) V(x, y)$. Then the LCU formula of g could be simplified as

$$g_2 = \mu \int dx p(x) \int dy q(x, y) V(x, y), \tag{S39}$$

with $\mu = \mu_1\mu_2$.

B. Spectral filter by randomised composite LCU formulae

Recall that we choose a Gaussian spectral filter $g_\tau(H) = e^{-\tau^2 H^2}$. Below, we elaborate on a few properties of the spectral filter. A general matrix function acting on the Hamiltonian is defined as

$$g(H) := \sum_{i=0}^{N-1} g(E_i) \left|u_i\right\rangle \left\langle u_i\right| . \tag{S40}$$

where $g(h) : \mathbb{R} \to \mathbb{C}$ is a generic continuous-variable function determining the transformation of the spectrum of the Hamiltonian. As a spectral filter, the function $g(h)$ is required to satisfy strictly non-increasing absolute value, $|g(h')| < |g(h)|,\ \forall |h'| > |h|$, and vanishing asymptotic value, $\lim_{\tau\to\infty} |g(\tau h')/g(\tau h)| = 0,\ \forall |h'| > |h|$, and is an even function, $g(h) = g(-h)$. In this work, we choose the Gaussian function $g(h) = e^{-h^2}$, corresponding to a generalised imaginary-time evolution $g(\tau H) = e^{-\tau^2 H^2}$. As shown in the main text, the Gaussian spectral filter is decomposed into the basis of real-time evolution and is further decomposed into elementary operations, either Pauli operators for general cases or symmetry-conserved operators for ancilla-free consideration.

Given an LCU form of g, Proposition 2 shows the error in observable estimation. Below we provide the proof of Proposition 2.

Proposition 6 (Formal version of Proposition 2)**.** *For a target operator g and its (μ, ε)-randomised LCU formula defined in Eq. (1), if we estimate the value on the unnormalised state $N_g(O) := \mathrm{Tr}(g\rho g^\dagger O)$ with an initial state ρ and observable O, then the distance between the mean estimator value $\hat{O}$ and the true value $N_g(O)$ is bounded by*

$$\varepsilon_N := |\hat{N}_{\tilde{g}}(O) - N_g(O)| \le \|O\|(2\mu^2\varepsilon + \varepsilon_n), \tag{S41}$$

with a success probability $1-\vartheta$. Here, we use the variant of the Hadamard test circuit for $N_s = \mu^4 \ln(2/\vartheta)/\varepsilon_n^2$ times and $\|O\|$ is the spectral norm of O. The error for the denominator is bounded as $\varepsilon_D := |\hat{D}_{\tilde{g}} - D_g| \le 2\mu^2\varepsilon + \varepsilon_n$. Given the error ε_D and ε_N, the error for the normalised observable expectation $\langle O\rangle_g = N_g(O)/D_g$ is

$$|\frac{\hat{N}_{\tilde{g}}(O)}{\hat{D}_{\tilde{g}}} - \frac{N_g(O)}{D_g}| \le \frac{1}{D_g}((\langle O\rangle + 1)\varepsilon_D + \varepsilon_N). \tag{S42}$$

Proof. (of Proposition 2)

Suppose $\tilde{g}$ is a (μ, ε)-RLCU formula of g. We first have

$$N_{\tilde{g}}(O) = \mathrm{Tr}(\tilde{g}_\tau(H - E_0) O \tilde{g}_\tau(H - E_0)) \tag{S43}$$

we have

$$\begin{aligned}
|N_{\tilde{g}}(O) - N_g(O)| &= |\,\mathrm{Tr}(\tilde{g}_\tau(H - E_0) O \tilde{g}_\tau(H - E_0)) - \mathrm{Tr}(g_\tau(H - E_0) O g_\tau(H - E_0))| \\
&\le \varepsilon(\|\tilde{g}_\tau\| + \|g_\tau\|)\|O\|_\infty \\
&\le 2\mu^2\varepsilon\|O\|_\infty
\end{aligned} \tag{S44}$$

Here, we have use the fact that $\|\rho\| \le 1$, $\|\tilde{g}_\tau\| \le \mu^2$, and $g = g^\dagger$.

Suppose we have the estimator $\hat{N}_{\tilde{g}}(O)$ defined in Eq. (25). The expectation range is $[-\sqrt{2}\mu^2\|O\|_\infty, \sqrt{2}\mu^2\|O\|_\infty]$. Using the Hoeffding bound, we have the following probability tail bound for the mean estimator $\hat{N}_{\tilde{g}}(O)$,

$$\Pr(|\hat{N}_{\tilde{g}}(O) - N_{\tilde{g}}(O)|) \le 2\exp(-\frac{N_s\varepsilon_n^2}{\mu^4}) \tag{S45}$$

By setting $N_s := \mu^4 \ln(2/\vartheta)/\varepsilon_n^2$, we have the estimation error

$$\varepsilon_N := |\hat{N}_{\tilde{g}}(O) - N_g(O)| \le \|O\|(2\mu^2\varepsilon + \varepsilon_n), \tag{S46}$$

with a success probability $1 - \vartheta$.

The result for the denominator can be similarly derived since

$$D_{\tilde{g}} := \langle \tilde{g}_\tau^2(H - E_0)\rangle = N_{\tilde{g}}(I). \tag{S47}$$

We have

$$\varepsilon_D := |\hat{D}_{\tilde{g}} - D_g| \leq 2\mu^2\varepsilon + \varepsilon_n. \tag{S48}$$

The error for the normalised observable expectation is

$$\begin{aligned}
|\frac{\hat{N}_{\tilde{g}}(O)}{\hat{D}_{\tilde{g}}} - \frac{N_g(O)}{D_g}| &= |\frac{(\hat{N}_{\tilde{g}}(O)D_g - N_g(O)D_g) + (N_g(O)D_g - \hat{D}_{\tilde{g}}(O)N_g)}{\hat{D}_{\tilde{g}}D_g}| \\
&\leq |\frac{N_g(O)\varepsilon_D + D_g\varepsilon_N}{\hat{D}_{\tilde{g}}D_g}| \\
&\leq |\frac{(D_g + N_g(O))\varepsilon_D + D_g\varepsilon_N}{D_g^2}| = \frac{1}{D_g}((\langle O\rangle_g + 1)\varepsilon_D + \varepsilon_N)
\end{aligned} \tag{S49}$$

□

The performance of our method in eigenstate property estimation can be evaluated by the error in constructing the RLCU formula. We note that the random sampling algorithm is not a deterministic state preparation method, as it cannot prepare the target state σ. However, when we focus on the property estimation of the target state, our scheme has similar performance to the normal Hamiltonian simulation methods. Specifically, the sample complexities of the former algorithms and the RCLU algorithm to learn the observable properties of the state are similar, as long as the norm of the estimator is a constant. This is guaranteed by Proposition 2. One can thus compare our method with other deterministic schemes at the same level.

S3. EIGENSTATE PROPERTY ESTIMATION

In this section, we provide error analysis for eigenstate property estimation described in Problem 1. We will provide the proof of gate complexity (Theorem 1) in the main text.

A. Estimator

Recall that the task in Problem 1 is to estimate the observable expectation on the target eigenstate $|u_j\rangle$ up to a certain precision ε, which is characterised by

$$\langle O\rangle = \frac{N(O)}{D} = \frac{\langle u_i|O|u_i\rangle}{\langle u_i|u_i\rangle} \tag{S50}$$

where the denominator D and the numerator N is defined in respect to the eigenstate $|u_i\rangle$. The unnormalised eigenstate can be effectively realised by applying a spectral filter $g_{\tau\to\infty}(H-\omega)$ to an initial state, which holds when $\omega = E_j$ and the imaginary-time becomes infinity. In this section, the eigenenergy is first assumed to be known a priori. The task with an unknown eigenenergy will be discussed in Sec. S3 F.

It is easy to see that we arrive at the ideal observable expectation when $\tau \to \infty$,

$$\langle O\rangle = \frac{N_{\tau\to\infty}(O)}{D_{\tau\to\infty}}, \tag{S51}$$

given a nonvanishing denominator, which is $D_{\tau\to\infty}(E_j) = |c_j|^2 = \eta$. Note that we assume that the spectral weight $\langle u_i|\psi_0\rangle$ is nonvanishing. The observable when considering a finite τ is estimated by

$$\langle O\rangle_\tau = \frac{N_\tau(O)}{D_\tau}. \tag{S52}$$

The denominator and the numerator have the same definition in Eq. (11). In practice, when considering finite gate complexity and sample complexity, the eigenstate property is estimated by

$$\hat{O}_{\tau,x_c,s_c} = \frac{\hat{N}_{\tau,x_c,s_c}(O)}{\hat{D}_{\tau,x_c,s_c}}. \tag{S53}$$

The selection of τ, x_c, s_c can be determined by analysing the error of $\hat{O}_{\tau,x_c,s_c}$ compared to the ideal value. As shown in Proposition 1, the spectral filter can be written as an RLCU formula. The spectral filter takes an explicit form of

$$g_\tau(H-\omega) = c\int_{-\infty}^{\infty} dxp(x)e^{ix\tau\omega}e^{-i\tau xH} \tag{S54}$$

The integrand is a real-time evolution with total real-time τx. Suppose we further use LCU formula to implement $e^{-i\tau xH}$, which takes the form of

$$e^{-i\tau xH} = \mu(x\tau)\sum_{\vec{r}\in\mathcal{K}_x} \Pr(\vec{r}, x\tau, \nu(x\tau))U_{\vec{r}} \tag{S55}$$

where we have follow the definition in Eq. (14): $\vec{r}$ specifies the unitary $U_{\vec{r}}$ involved in the LCU formula of $e^{-i\tau xH}$, $\Pr(\vec{r}, x\tau, \nu(x\tau))$ represents the normalised decomposition coefficients of $U_{\vec{r}}$. Then, we have

$$g_\tau(H-\omega) = c(\mu)\int_{-\infty}^{\infty} dxp_\mu(x)e^{ix\tau\omega}\sum_{\vec{r}\in\mathcal{K}_x} \Pr(\vec{r}, x\tau, \nu(x\tau))U_{\vec{r}}. \tag{S56}$$

B. Error analysis

Now, we discuss the errors when considering finite τ, x_c, s_c. Note that Eq. (S56) is a composite LCU formula, more specifically, it is a $(c(\mu), 0)$-LCU formula. When we consider a finite x_c, s_c, it will introduce some errors. The key idea of analysing the errors is to compare the operator distance between

$$\|g_{\tau,x_c,s_c} - g_{\tau,x_c,s_c\to\infty}\| \tag{S57}$$

which will gives a bound for the numerator and the denominator.

a. Error due to finite τ. The imaginary-time τ determines the strength of the spectral filter. The spectral weight on the unwanted eigenstates, namely those energies away from the pre-set parameters E, will be exponentially suppressed. In the infinite time limit, g will effectively project out all the spectral weights on the unwanted eigenstates, given that the initial state has a nonvanishing spectral weight on the target eigenstate. The error of the denominator and numerator can be analysed by considering the operator distance between g_τ and $g_{\tau\to\infty}$. In this section, we choose $\omega = E_j$ and omit ω when there is no ambiguity.

Proposition 7 (Error due to a finite τ)**.** *When $\tau \geq \frac{1}{\Delta}\sqrt{\ln(2/\varepsilon_\tau)}$, the error of the denominator and the numerator that are defined in Eq. (11) when compared with those defined with respect to the ideal eigenstates in Eq. (S50) satisfy $|D_\tau - D| \leq \varepsilon_\tau$, $|N_\tau(O) - N(O)| \leq \varepsilon_\tau\|O\|$.*

Proof. The distance between $g_\tau(H-E_j)$ and $g_{\tau\to\infty}(H-E_j)$ can be bounded by

$$g_\tau(H-E_j) - g_{\tau\to\infty}(H-E_j) = \sum_{i\neq j} g_\tau(E_i - E_j)\,|u_i\rangle\,\langle u_i| \tag{S58}$$

where $g_\tau(H-\omega)$ is defined in Eq. (S56). According to the definition of g_τ, when $\tau \geq \frac{1}{\Delta}\sqrt{\ln(2/\varepsilon_\tau)}$, it is easy to check that

$$g_\tau(E_i - E_j) \leq \varepsilon_\tau/2,\ \forall i \neq j \tag{S59}$$

and thus we have

$$\|g_\tau(H-E_j) - g_{\tau\to\infty}(H-E_j)\| \leq \varepsilon_\tau/2 \tag{S60}$$

Using the result in Proposition 2, the error of the numerator can be bounded by

$$N_\tau(O) - N(O) \leq \varepsilon\|O\| \tag{S61}$$

The error of the denominator can be obtained straightforwardly by taking $O = I$.

□

b. Error due to finite truncation in real-time evolution. Then we analyse the error due to finite x_c and s_c. In practice when the integral is truncated to from infinite range $[-\infty, \infty]$ a finite time length $[-x_c, x_c]$, $g_\tau(H-\omega)$ becomes

$$g_{\tau,x_c}(H-\omega) = c\int_{-x_c}^{x_c} dx p(x) e^{ix\tau\omega} e^{-i\tau x H}. \tag{S62}$$

Using the property of the Gaussian tail, it is easy to verify that the truncation error of g_τ with a finite x_c is given by

$$\|g_\tau - g_{\tau,x_c}\| \leq \varepsilon_{x_c} \tag{S63}$$

when $x_c \geq 2\sqrt{\ln(1/\varepsilon_{x_c})}$.

As shown in the main text, we consider dealing with the real-time evolution using Trotter-LCU expansion. For evolution time t, let us denote the real-time evolution $U(t) := e^{-iHt} = U_m^\nu$ where

$$U(m) = e^{-imH} = \mu(m)\mu(m)\sum_r \Pr(r, m, \nu(m))W_r$$

is a short time dynamics during the time interval $m = t/\nu$. In a $2k$th-order Trotter-LCU algorithm, the overall LCU formula for $U(m)$ is

$$U(m) = V_{2k}(m)S_{2k}(m), \tag{S64}$$

which consists of a deterministic $2k$th-order Trotter formula S_{2k} and the Trotter error compensation term V_{2k}. We consider a truncated Trotter-LCU formula to realize a (μ, ε)-LCU formula for $U(t) = e^{-iHt}$ based on ν segments of $\tilde{U}_{2k}$ in Eq. (S70).

We will use the results established in [57] to analyse the segment number ν to control the error to a certain level. The complexity of the zeroth-order leading-order-pairing algorithm scales quadratically to t, which is undesirable for simulation with a long time. If we apply $2k$th-order Trotter formula $S_{2k}(m)$ in each segment, the $2k$th-order remainder is $V_{2k}(m) := U(m)S_{2k}(m)^\dagger$. Using BCH formula, we can have an explicit form of $V_{2k}(m)$

$$V_{2k}(m) = \exp(i\sum_{s=2k+1}^{\infty} E_{2k,s}m^s) = \sum_{s=0}^{\infty} F_{2k,s}(m) = \sum_{s=0}^{\infty} \|F_{2k,s}(m)\| V_{2k}^{(s)}(m) \tag{S65}$$

where we group the terms by the order of m, and $F_{1,s}$ denotes the s-order expansion term of $V_{2k}(m)$ with m^s. Here, $\|F_{2k,s}(m)\|_1$ is the 1-norm of the s-order expansion formula $F_{2k,s}(m)$ and $V_{2k,s}(m)$ is the normalised LCU formula for the s-order terms. It is easy to see that

$$F_{2k,s}(m) = im^s E_{2k,s}, \;\; s \in [2k+1, 4k+1] \tag{S66}$$

Formally, we can express

$$E_{2k,s} = \lambda_r \sum_r \Pr(r|s) P_{2k}(r). \tag{S67}$$

By pairing the sth order expansion term with the identity I, the order of $F_{2k,s}(m)$ can be doubled as

$$I + F_{2k,s}(m) = \lambda_r \sqrt{1+m^{2s}} \sum_r \Pr(r|s)\exp(i\arctan(m^{2s})P_{2k}(r). \tag{S68}$$

Therefore, the norm of $F_{2k,s}$ decreases from $\mathcal{O}(1+m^s)$ to $\mathcal{O}(1+m^{2s})$. Here we note that this holds for the symmetry-conserved LCU decomposition where the elementary operations are SWAP and Pauli-Z operators.

The error can be shown to have a quick decrease with an increasing truncation order s_c. Considering of a finite truncation and the vanishing of $F_{2k,j\leq 2k}(m) = 0$, we can rewrite $V_{2k}^{(s)}(m)$ as

$$V_{2k}^{(s)}(m) = I + \sum_{s=2k+1}^{s_c} F_{2k,s}(m). \tag{S69}$$

Given the truncation, the LCU formula for $U(m)$ is

$$U_{2k}^{(s_c)}(m) = V_{2k,s}(m)S_{2k}(m). \tag{S70}$$

The overall LCU formula for $U(t)$ is to repeat the sampling of $\tilde{U}_{2k}(x)$ for ν times. The overall LCU formula for time t is

$$U_{2k}^{(s_c)}(t) = \left(U_{2k}^{(s_c)}(m)\right)^{\nu} \tag{S71}$$

with $\nu = t/m$. Here $U_{2k}^{(s_c)}(t)$ denotes the RLCU formula of U when truncating it to s_c orders and t is the total evolution time, and $U_{2k}^{(s_c)}(t)$ is a $(\mu_{2k,tot}(t), \varepsilon_{2k,tot}(t))$-LCU formula of $U(t)$ with

$$\mu_{2k,tot}(t) = \mu(m)^{\nu},\ \varepsilon_{2k,tot}(t) \leq \nu\mu_{2k,tot}(t)\varepsilon_{2k}(m) \tag{S72}$$

The key component is to analyse the quantum resources needed to achieve an additive error of the approximation given by Eq. (17)

$$\|U(t) - U_{2k}^{(s_c)}(t))\| \leq \varepsilon_{s_c}. \tag{S73}$$

Lemma 1 gives the Hamiltonian simulation error ε_{s_c} when using 2kth-order paired Taylor-series compensation with finite s_c.

Lemma 1 (Hamiltonian simulation error using 2kth-order paired Taylor-series compensation (Theorem 1 in [57]))**.** *In a 2kth-order Trotter-LCU algorithm, if the segment number ν and the truncation order s_c satisfy*

$$\begin{aligned} \nu &\geq \left(\frac{2(e+c_k)\lambda t}{\ln\mu}\right)^{\frac{1}{4k+1}} 2\lambda t, \\ s_c &\geq \max\left\{\left\lceil \frac{\ln\left(\frac{\mu}{\varepsilon}\nu_{2k}(t)\right)}{W_0\left(\frac{1}{2e\lambda t}\nu_{2k}(t)\ln\left(\frac{\mu}{\varepsilon}\nu_{2k}(t)\right)\right)} - 1\right\rceil, 4k+1\right\}, \end{aligned} \tag{S74}$$

we can then realize a (μ, ε)-LCU formula for $U(t) = e^{-iHt}$, i.e., $\|U(t) - U_{2k}^{(s_c)}(t))\| \leq \varepsilon_{s_c}$, based on ν segments of $\tilde{U}_{2k}$ in Eq. (S70). Here, c_k is defined as $c_k := \frac{1}{2}\left(\frac{e}{2k+1}\right)^{4k+2}$.

When considering a finite s_c, the spectral filter becomes

$$g_{\tau,x_c,s_c}(H-\omega) = c\int_{-x_c}^{x_c} dx p(x) e^{ix\tau E} U_{2k}^{(s_c)}(x\tau, \nu(x\tau)) \tag{S75}$$

It is easy to check that $\|g_{\tau,x_c,s_c}(H-\omega)\| \leq c(1+\varepsilon_{s_c})$. The operator distance between g_{τ,x_c} and g_{τ,x_c,s_c} due to finite s_c is

$$\|g_{\tau,x_c} - g_{\tau,x_c,s_c}\| \leq c\int_{-x_c}^{x_c} dx p(x) e^{ix\tau\omega} \|U(x\tau) - U_{2k}^{(s_c)}(x\tau, \nu(x\tau))\| \leq \varepsilon_{s_c} \tag{S76}$$

when $\|U - U_{2k}^{(s_c)}(x\tau, \nu(x\tau))\| \leq \varepsilon_{s_c}/c$.

Using the triangular inequality, the operator error between g_τ and g_{τ,x_c,s_c} defined in Eq. (S75) is

$$\|g_\tau - g_{\tau,x_c,s_c}\| \leq \varepsilon_{x_c} + \varepsilon_{s_c} \tag{S77}$$

With the error due to a finite time when evaluating the integral Eq. (S63) and the truncation order s_c Eq. (S76), we arrive at the following Proposition 8.

Proposition 8 (Error due to finite time length and truncation)**.** *When $x_c \geq 2\sqrt{\ln(2/\varepsilon_{x_c})}$, ν_c and s_c satisfying Eq. (S74), we have $|D_{\tau,x_c,s_c} - D_\tau| \leq 3\varepsilon_c$, $|N_{\tau,x_c,s_c}(O) - N_\tau(O)| \leq 3\varepsilon_c\|O\|$ with $\varepsilon_c = \varepsilon_{x_c} + \varepsilon_{s_c}$*

Proof. The estimator

$$\begin{aligned} |N_\tau(O) - N_{\tau,x_c,s_c}(O)| =& |\langle\psi_0|g_\tau(H-\omega)Og_\tau(H-\omega)|\psi_0\rangle - \langle\psi_0|g_{\tau,x_c,s_c}(H-\omega)Og_{\tau,x_c,s_c}(H-\omega)|\psi_0\rangle \\ \leq& |\langle\psi_0|g_\tau(H-\omega)Og_\tau(H-\omega)|\psi_0\rangle - \langle\psi_0|g_{\tau,x_c,s_c}(H-\omega)Og_\tau(H-\omega)|\psi_0\rangle| \\ &+ |\langle\psi_0|g_{\tau,x_c,s_c}(H-\omega)Og_\tau(H-\omega)|\psi_0\rangle - \langle\psi_0|g_{\tau,x_c,s_c}(H-\omega)Og_{\tau,x_c,s_c}(H-\omega)|\psi_0\rangle \\ \leq& \varepsilon_c(\|g_\tau\| + \|g_{\tau,x_c,s_c}\|)\|O\| \leq 3\varepsilon_c\|O\| \end{aligned} \tag{S78}$$

where $\varepsilon_c := \varepsilon_{x_c} + \varepsilon_{s_c}$. In the third inequality, we have used the inequality by Eq. (S77).

□

The gate complexity of the overall algorithm can then be estimated. To construct controlled-$U(t)$, we split it to ν segments. In each segment, we need to implement kth-order Trotter circuits and random Taylor-series sampling circuits. The number of gates in the random Taylor-series sampling circuit is $\mathcal{O}(s_c)$. Therefore, the gate complexity of the overall algorithm using Kth Trotter formula ($K = 0, 1, 2k$) is given by

$$N_K = \mathcal{O}(\nu(\kappa_K L + s_c)), \tag{S79}$$

where

$$\kappa_K = \begin{cases} K, & K = 0,\ 1 \\ 2 \cdot 5^{K/2-1}, & K = 2k,\ k \in \mathbb{N}_+. \end{cases} \tag{S80}$$

c. Unbiased estimator. Recall that to estimate $\langle \vec{\mathbf{j}} | O | \vec{\mathbf{i}} \rangle$, the Hadamard-test type of circuit in Fig. 1(c1) in the main text is used. In Fig. 1(c1), we first prepare the state $|\psi_0\rangle$ and an extra ancillary qubit prepared on $|+\rangle$. Afterwards, we perform a C-U gate from ancillary to $|\psi_0\rangle$. If we directly measure the ancillary qubit on the X-basis, the outcome a will be 0 with a probability of $\Pr(a = 0) = \frac{1}{2}(1 + \mathrm{Re}(\langle \vec{\mathbf{j}} | O | \vec{\mathbf{i}} \rangle)$ and 1 with a probability of $\Pr(a = 1) = \frac{1}{2}(1 - \mathrm{Re}(\langle \vec{\mathbf{j}} | O | \vec{\mathbf{i}} \rangle)$. Then, similarly, we repeat the circuit but with an inverted phase gate $S^\dagger = R_z(-\frac{\pi}{2})$ before measurement. The outcome b will be 0 with a probability of $\Pr(b = 0) = \frac{1}{2}(1 + \mathrm{Im}(\langle \vec{\mathbf{j}} | O | \vec{\mathbf{i}} \rangle)$ and 1 with a probability of $\Pr(b = 1) = \frac{1}{2}(1 - \mathrm{Im}(\langle \vec{\mathbf{j}} | O | \vec{\mathbf{i}} \rangle)$.

Proposition 9. *The estimator $\hat{v}$ defined in Eq. (25) is an unbiased estimator of $\langle \psi_0 | g_\tau(H - \omega) O g_\tau(H - \omega) \psi_0 \rangle$.*

Proof. Taking an average over a, b, r we have

$$\mathbb{E}_{a,b,r} \hat{v} = c^2(\mu) e^{i\tau\omega(t_i - t_j)} \mathbb{E}_{a,b,r} \hat{d} = c^2(\mu) e^{i\tau\omega(t_i - t_j)} = c^2(\mu) e^{i\tau\omega(t_i - t_j)} \langle \vec{\mathbf{j}} | O | \vec{\mathbf{i}} \rangle \tag{S81}$$

Taking an average of $t_i, t_j, \vec{i}, \vec{j}$, we have

$$\begin{aligned} \mathbb{E}_{t_i, t_j, \vec{i}, \vec{j}} \mathbb{E}_{a,b,r} \hat{v} &= c^2(\mu) \mathbb{E}_{t_i, t_j} e^{i\tau\omega(t_i - t_j)} \mathbb{E}_{\vec{i}, \vec{j}} \langle \vec{\mathbf{j}} | O | \vec{\mathbf{i}} \rangle \\ &= c^2(\mu) \sum_{i,j} \Pr(i) \Pr(j) e^{i\tau\omega(t_i - t_j)} \sum_{\vec{i}, \vec{j}} \Pr(\vec{i}, \tau t_i, \nu) \Pr(\vec{j}, \tau t_j, \nu) \langle \vec{\mathbf{j}} | O | \vec{\mathbf{i}} \rangle \\ &= \langle \psi_0 | g_\tau(H - \omega) O g_\tau(H - \omega) \psi_0 \rangle = N_\tau(O). \end{aligned} \tag{S82}$$

□

In the other case, where $O = \sum_l o_l P_l$ is composed of many terms as described in Problem 1, an importance sampling method can be used to estimate the observable. Compared to measuring each term separately, the measurement cost using importance sampling is independent of L. In particular, we sample the observables' index l from the probability distribution $o_l / \|O\|_1$. Given a sampled l, we define the estimator,

$$\hat{d}_l = (-1)^{a_l} + i(-1)^{b_l},$$

in a similar way to that in Methods. It is easy to see that $\hat{d}_l$ is an unbiased estimator of $\langle \vec{\mathbf{j}} | O | \vec{\mathbf{i}} \rangle$ as

$$\mathbb{E}_{a,b,l} \hat{d}_l = \langle \vec{\mathbf{j}} | O_l | \vec{\mathbf{i}} \rangle, \tag{S83}$$

as one can verify that

$$\mathbb{E}_{a_l} (-1)^{a_l} = \mathrm{Re}(\langle \vec{\mathbf{j}} | O_l | \vec{\mathbf{i}} \rangle),\ \mathbb{E}_{b_l} (-1)^{b_l} = \mathrm{Im}(\langle \vec{\mathbf{j}} | O_l | \vec{\mathbf{i}} \rangle). \tag{S84}$$

Similarly, we define the estimator as

$$\hat{v}_l = c^2(\mu) e^{i\tau\omega(t_i - t_j)} \hat{d}_l \tag{S85}$$

Taking the average of $t_i, t_j, \vec{i}, \vec{j}, a, b, r, l$, we have

$$\mathbb{E}_{t_i, t_j, \vec{i}, \vec{j}, a, b, r, l} \hat{v}_l = N_\tau(O). \tag{S86}$$

The range of the estimator is $[-\sqrt{2}c^2(\mu)\|O\|_1, \sqrt{2}c^2(\mu)\|O\|_1]$. Using Hoefflding inequality, the estimation error can be bounded

$$
\begin{aligned}
|\hat{D}_{\tau,x_c,s_c} - D_{\tau,x_c,s_c}| &\leq \varepsilon_n \\
|\hat{N}_{\tau,x_c,s_c}(P_l) - D_{\tau,x_c,s_c}(P_l)| &\leq \varepsilon_n \|O\|_1
\end{aligned}
\tag{S87}
$$

with a success probability of $1-\vartheta$ when the number of measurements $N_s \geq 2c^4(\mu)\frac{1}{\varepsilon_n^2}\ln(1/\vartheta)$. In this work, we will set $c(\mu)=2$. We have the following result.

Proposition 10 (Error due to finite measurements using Hadamard test)**.** *$|\hat{D}_{\tau,x_c,s_c} - D_{\tau,x_c,s_c}| \leq \varepsilon_n$ and $|\hat{N}_{\tau,x_c,s_c}(O) - N_{\tau,x_c,s_c}(O)| \leq \varepsilon_n$ has a success probability of $1-\vartheta$ when the number of measurements $N_s \geq 2c^4(\mu)\frac{\|O\|_1^2}{\varepsilon_n^2}\ln(1/\vartheta)$.*

d. Measurement strategy. To measure $\langle \vec{\mathbf{j}}|O|\vec{\mathbf{i}}\rangle$, we can use the circuit in Fig. 1(c) to generate

$$
U_p^\dagger(\prod_{q=1}^{\nu} W_{i_q} S)^\dagger O \prod_{q=1}^{m} W_{i_q} S U_p |\psi_0\rangle\,, \tag{S88}
$$

and then measure on a computational basis.

In many practical cases, such as Heisenberg models and electronic structure problems (Eq. (7)), the target problem has certain symmetries $\mathcal{S}$ satisfying $[H,\mathcal{S}]=0$. Consequently, the state can be divided into state spaces with different symmetry sectors. For instance, for fermionic problems, the Hamiltonian has particle number symmetry, $[H,\hat{N}]=0$ with $\hat{N}=\sum_i \hat{a}_i^\dagger \hat{a}_i$. In this case, the state can be divided into different sectors in the Hilbert space with different particle numbers, $\mathcal{H}:\mathrm{span}\{|i\rangle\}$ with $|i\rangle$ representing the state with i particles. We can use the ancilla-free measurement strategy in Fig. 1(c2). Note that in this case, the operator W_{i_q} must be symmetry preserving.

Recall that the requirements of this protocol include the preparation of the superposition state and efficient computation of $\langle\psi_{\mathrm{Ref}}|U|\psi_{\mathrm{Ref}}\rangle$. Let us take the fermionic Hamiltonian as an example (note that the Heisenberg model can be regarded as a 1D Fermi-Hubbard model after the Jordan-Wigner transformation). If the reference state is the vacuum state with the number of particles being zero, then $\langle\psi_{\mathrm{Ref}}|U|\psi_{\mathrm{Ref}}\rangle$ can be computed classically. The Hartree-Fock state takes the simple product-state form $|\psi_0\rangle = |1\rangle^{\otimes N_e}|0\rangle^{\otimes N-N_e} \in \{|N_e\rangle\}$, where the first N_e qubits are prepared in the $|1\rangle$ states and the rest of the qubits remain in the $|0\rangle$ states. To prepare the target superposition state $|\phi_0\rangle$, a Hadamard gate is first applied to the initial qubit, followed by a chain of CNOT gates up to the N_eth qubit, requiring a total of N_e-1 CNOT operations. However, we can alternatively choose a reference state that differs from the initial state by only a single site. In this case, the preparation of the superposition state becomes simpler, requiring only one additional CNOT gate compared to the original state.

We have adopted this ancilla-free measurement strategy in the experiments, which avoids decomposing long-range controlled gates into local operations and hence has a low compilation overhead. That said, in our resource estimates we used a relatively conservative evaluation, ensuring that the reported results remain applicable even if the first method are employed instead.

C. Proof of Theorem 1 (Problem 1)

When τ is taken as a finite value, g_τ can be regarded a $(1,\varepsilon_\tau)$-RLCU formula of g. The integral is evaluated within a finite time range,

$$
g_{\tau,x_c} = \int_{-x_c}^{x_c} dx p(x) U(x\tau) \tag{S89}
$$

One can check that g_{τ,x_c} is a $(1,\varepsilon_{x_c})$-RLCU formula of g_τ defined in Eq. (10), and is a $(1,\varepsilon_{x_c}+\varepsilon_\tau)$-RLCU formula of $g_{\tau\to\infty}$. Each of the integrand $U(x\tau)$ in g_{τ,x_c} is approximated by $U_{2k}^{(s_c)}(x\tau)$ defined in Eq. (S70). According to Proposition 8 and Lemma 1, when considering a segment number ν_c and the truncation order s_c defined in Eq. (S95), $U_{2k}^{(s_c)}(x\tau)$ is a $(\mu_2(x),\varepsilon_x)$-LCU formula, where upper bounds of μ_2 and ε_x can be obtained by Proposition 1.

According to Proposition 1, g_{τ,x_c,s_c}, which is defined as

$$
g_{\tau,x_c,s_c} = \int_{-x_c}^{x_c} dx p(x) \tilde{U}(x\tau) \tag{S90}
$$

is a (μ,ε)-LCU formula of g, with $\mu := \int_{-\infty}^{\infty} p(x)\mu_2(x)dx \leq \max_x \mu_2(x)$, and $\varepsilon = \varepsilon_c + \varepsilon_\tau + \varepsilon_{s_c}$.

Next, we analyse the error due to finite measurements. In the case where we can directly measure in the eigenbases of the observable O, we can take the estimator in Eq. (25). Then the error of the expectation value of observable can be bounded by directly using Proposition 2. The error distance can be bounded in a similar fashion to Proposition 2. For the case with observables composed of many Pauli terms. Suppose we measure it using importance sampling; the only difference is the amplification of the prefactor by $\|O\|_1$.

Equipped with all these results, we arrive at the following result of eigenstate property estimation.

Theorem 5 (Eigenstate property estimation for generic Hamiltonians)**.** *Suppose the conditions and assumptions in Problem 1 hold. Suppose we use the method in Algorithm 1 where we choose the time-segment number function* $\nu_c = \mathcal{O}\left((\lambda\Delta^{-1}\ln(\eta^{-1}\varepsilon^{-1}))^{1+\frac{1}{4k+1}}\right)$ *for realising the real-time evolution (in Eq. (S94)) and the truncation order* $s_c = \mathcal{O}(\ln(\nu_c/\varepsilon_c)/\ln\ln(\nu_c/\varepsilon_c)))$*. We can achieve the error of observable's expectation on the eigenstate* $|u_j\rangle$ *within* ε, $|\hat{v} - \langle u_j|O|u_j\rangle| \leq \varepsilon$ *when the sampling number is* $N_s = \mathcal{O}\left(\eta^{-2}\varepsilon^{-2}\|O\|_1^2\ln(1/\vartheta)\right)$ *(in Eq. (S97)), where* $\hat{v}$ *is the estimator defined in Eq. (25) with a success probability at least* $1-\vartheta$*.*

Proof. We set the imaginary-time as

$$\tau \geq \frac{1}{\Delta}\sqrt{\ln\frac{2}{\varepsilon_\tau}} \tag{S91}$$

and set truncation time x_c

$$x_c \geq 2\sqrt{\ln(2/\varepsilon_c)}. \tag{S92}$$

The maximum real-time is

$$t_c = \tau x_c = \frac{2}{\Delta}\sqrt{\ln(2/\varepsilon_\tau)}\sqrt{\ln(2/\varepsilon_c)} \tag{S93}$$

According to Proposition 8, when the segment number is set as

$$\nu_c = 2\left(\frac{2(e+c_k)}{\ln 2}\right)^{\frac{1}{4k+1}}(\lambda\tau x_c)^{1+\frac{1}{4k+1}} = 4\left(\frac{4(e+c_k)}{\ln 2}\right)^{\frac{1}{4k+1}}(\frac{\lambda}{\Delta}\sqrt{\ln(2/\varepsilon_\tau)\ln(2/\varepsilon_c)})^{1+\frac{1}{4k+1}} \tag{S94}$$

and set

$$s_c = \mathcal{O}\left(\frac{\ln\left(\frac{4\nu_c}{\varepsilon_{s_c}}\right)}{\ln\left(\nu_c^{\frac{1}{4k+2}}\ln\left(\frac{4\nu_c}{\varepsilon_{s_c}}\right)\right)}\right) \tag{S95}$$

$U_{2k}^{(s_c)}(t_i)$ is a $(2,\varepsilon_{s_c})$-LCU formula of $U(t_i)$, and the approximation error is given by

$$\max_{t_i}\|U_{2k}^{(s_c)}(t_i) - U(t_i)\| \leq \varepsilon_{s_c}. \tag{S96}$$

According to Proposition 8, we have $|D_{\tau,x_c,s_c} - D_\tau| \leq \varepsilon_c$, $|N_{\tau,x_c,s_c}(O) - N_\tau(O)| \leq \varepsilon_c\|O\|$ with $\varepsilon_c = 3(\varepsilon_{x_c}+\varepsilon_{s_c})$
We set the sampling number as

$$N_s = \frac{2c^4(\mu)\|O\|_1^2}{\varepsilon_n^2}\ln(1/\vartheta). \tag{S97}$$

According to Proposition 10, $|\hat{D}_{\tau,x_c,s_c} - D_{\tau,x_c,s_c}| \leq \varepsilon_n$ and $|\hat{N}_{\tau,x_c,s_c}(O) - N_{\tau,x_c,s_c}(O)| \leq \varepsilon_n$ has a success probability of $1-\vartheta$ when the number of measurements $N_s \geq 32\frac{\|O\|_1^2}{\varepsilon_n^2}\ln(1/\vartheta)$. Using the triangular inequality, we have

$$\begin{aligned}\varepsilon_N &:= |\hat{N}_{\tau,x_c,s_c}(O) - N_{\tau\to\infty}(O)| \leq \|O\|(\varepsilon_\tau+\varepsilon_c) + \|O\|_1\varepsilon_n,\\ \varepsilon_D &:= |\hat{D}_{\tau,x_c,s_c} - D_{\tau\to\infty}| \leq \varepsilon_\tau+\varepsilon_c+\varepsilon_n,\end{aligned} \tag{S98}$$

The observable's error in respect to ε_τ, ε_c and ε_n is given by

$$
\begin{aligned}
\left|\langle\hat{O}\rangle_{\tau,x_c,s_c}-\langle O\rangle\right| &= \left|\frac{\hat{N}_{\tau,x_c,s_c}(O)}{\hat{D}_{\tau,x_c,s_c}}-\frac{N(O)}{D}\right| \\
&= \left|\frac{D\hat{N}_{\tau,x_c,s_c}(O)-N(O)\hat{D}_{\tau,x_c,s_c}}{D\hat{D}_{\tau,x_c,s_c}}\right| \\
&\leq \left|\frac{D\left(N(O)+\varepsilon_N(O)\right)-N(O)\left(D-\varepsilon_D\right)}{D\left(D-\varepsilon_D\right)}\right| \\
&= \left|\frac{D\varepsilon_N(O)+N(O)\varepsilon_D}{D^2-D\varepsilon_D}\right| \\
&\leq \left|\frac{D\varepsilon_N(O)+\left(N(O)+D\right)\varepsilon_D}{D^2}\right| \\
&= \eta^{-1}\left(\left(\varepsilon_\tau+\varepsilon_c\right)\|O\|+\varepsilon_n\|O\|_1\right)+\eta^{-1}\left(\langle O\rangle+1\right)\left(\varepsilon_\tau+\varepsilon_c+\varepsilon_n\right) \\
&\leq \eta^{-1}\left(2\|O\|+1\right)\left(\varepsilon_\tau+\varepsilon_c\right)+\eta^{-1}\left(\|O\|+\|O\|_1+1\right)\varepsilon_n .
\end{aligned} \tag{S99}
$$

If we set the precision for each component

$$
\begin{aligned}
\varepsilon_\tau = \varepsilon_c &= \frac{1}{3}\eta\varepsilon\left(\frac{1}{2\|O\|_\infty+1}\right), \\
\varepsilon_n &= \frac{1}{3}\eta\varepsilon\frac{1}{\|O\|_\infty+\|O\|_1+1},
\end{aligned} \tag{S100}
$$

then we have

$$
\left|\langle\hat{O}\rangle_{\tau,x_c,s_c}-\langle O\rangle\right| \leq \varepsilon \tag{S101}
$$

for observable O with a success probability bounded by $1-\vartheta$.

Under the precision requirement in Eq. (S100), we know from Eq. (S94) that the segment number scales as

$$
\nu_c = \mathcal{O}\left((\lambda\Delta^{-1}\ln(\eta^{-1}\varepsilon^{-1}))^{1+\frac{1}{4k+1}}\right) \tag{S102}
$$

and the sampling number scales as

$$
N_s = \mathcal{O}\left(\eta^{-2}\varepsilon^{-2}\|O\|_1^2\ln(1/\vartheta)\right). \tag{S103}
$$

□

Importantly, the actual quantum resources can be directly estimated by Eq. (S94) and Eq. (S100) (and thus Eq. (S106)) and Eq. (S97).

D. Gate complexity analysis

Using the results in Proposition 3 and Theorem 5, we have the following result for generic Hamiltonians.

Theorem 6 (Gate complexity for generic Hamiltonians' eigenstate property estimation)**.** *Suppose we are given the same condition in Theorem 5. Suppose we synthesise the circuit to* CNOT *gates, single-qubit Clifford gates and single-qubit Z rotation gates, we have gate count*

$$
\mathrm{C}_{\mathrm{CNOT}} = \mathcal{O}(\mathrm{wt}(H)\nu_c),\ \mathrm{C}_{R_z} = \mathcal{O}(L\nu_c) \tag{S104}
$$

with ν_c given by Eq. (S106).

Proof. We can calculate the maximum required evolution time based on Theorem Theorem 5 with Gaussian function if we want to estimate an observables O with an accuracy ε.

For the ground state preparation, the maximum real-time is given by Eq. (S93). Suppose we divide the time slice into ν segments and use $2k$th order Trotterisation, in this context, the segment number ν can be chosen as $\nu_c = \mathcal{O}((\lambda\Delta^{-1}\ln(\eta^{-1}\varepsilon^{-1}))^{1+\frac{1}{4k+1}})$. We note that since there is a saturation of the gate count for the Trotter-error-compensation indicated by Eq. (27), we take s_c to be infinity in deriving the asymptotic scaling, and the gate count

is less than or at the same order of L or $\mathrm{wt}(H)$. Therefore, the gate complexity in each sample is given by Eq. (S104). In cases where we could implement each $e^{-iH_l t}$, then the gate complexity in each single run is

$$\mathcal{O}(L(\lambda\Delta^{-1}\ln(\eta^{-1}\varepsilon^{-1}))^{1+\frac{1}{4k+1}}). \tag{S105}$$

Thus far, we have completed the proof of Theorem 1.

□

Suppose O is simply a Pauli operator, $\|O\| = 1$. A more explicit form for the segment number can be obtained using Eq. (S100). With some derivation, we have

$$\nu_c = 4\left(\frac{4(e+c_k)}{\ln 2}\right)^{\frac{1}{4k+1}} (\frac{\lambda}{\Delta}\ln(\frac{9}{\eta\varepsilon}))^{1+\frac{1}{4k+1}}, \tag{S106}$$

which can be used to carry out resource estimations when given the parameters of the Hamiltonian.

Theorem 5 gives an upper bound on the gate complexity required for estimating generic Hamiltonians' eigenstate properties in relation to λ, L, and $\mathrm{wt}(H)$. For physical Hamiltonians, we could reduce the gate complexity by using the properties of the Hamiltonians. As shown in Theorem 2 in [57], the segment number can be reduced to

$$\nu_c = \mathcal{O}(n^{\frac{2}{4k+1}}(\Delta^{-1}\ln(\eta^{-1}\varepsilon^{-1}))^{1+\frac{1}{4k+1}}). \tag{S107}$$

Using Proposition 3, we can obtain the gate complexity for lattice models. In short, by using the commutation relation of the Hamiltonian terms in real-time evolution, the gate complexity for eigenstate property estimation using Algorithm 1 scales $\mathcal{O}(n^{1+\frac{2}{4k+1}})$. When using the ancilla-free LCU formulae and the corresponding measurement strategy, the gate operations within each segment can be implemented in parallel, as discussed in Sec. D. Therefore, the depth complexity is $\mathcal{O}(n^{\frac{2}{4k+1}})$. Therefore, we have completed the proof of Theorem 2 in the main text

E. Discussions on molecular Hamiltonians

The following discusses the resource cost for fermionic problems with the Hamiltonian Eq. (7) in the main text and its qubit form given by Eq. (S108). Note that each term in the expansion of V_i is a Pauli operator. Therefore, at each segment, δt, the gates that effectively implement the remainder will merge into a single Pauli operator. This means that the gate count for Trotter-error compensation will saturate to n, regardless of the truncation order s_c.

The Trotter formula will be implemented in a split-operator way. The nonlocal kinetic term will be converted to local terms with an additional cost for implementing the diagonalisation. However, to derive the remainder, we still need to expand all the terms in the Pauli basis. The second-quantised Hamiltonian given by Eq. (7) can be mapped by the Jordan-Wigner transform to a qubit Hamiltonian of the form

$$H = \hat{T} + \hat{V} = \sum_{pq}\tilde{T}_{pq}\left(X_p Z_{p+1}Z_{p+2}\cdots Z_{q-1}X_q + Y_p Z_{p+1}Z_{p+2}\cdots Z_{q-1}Y_q\right) + \sum_p \tilde{U}_p Z_p + \sum_{pq}\tilde{V}_{pq}Z_p Z_q \tag{S108}$$

A direct application of our zeroth-order method gives CNOT gate count: $\mathrm{wt_m}(H)(s_c{+}2)\nu = \mathcal{O}(n(s_c{+}2)\nu) = \mathcal{O}(n(s_c{+}2)(\lambda t)^2)$, and 2kth order CNOT gate count gives $(\mathrm{wt_m}(H)s_c + 2\,\mathrm{wt}(H))\nu = \mathcal{O}(n^3(\lambda t)^{1+\frac{1}{4k+1}})$ with $\mathrm{wt}(H) = nL = n^3$. However, the kinetic operator is quadratic and thus can be diagonalised by an efficient circuit transformation C

$$\hat{T} = C\left(\sum_p T_p Z_p\right)C^\dagger \tag{S109}$$

The second-order Trotterised time evolution is

$$S_2 = (e^{-iTx/2}e^{-iVx}e^{-iTx/2})^\nu = (Ce^{-iTx/2}C^\dagger e^{-iVx}Ce^{-iTx/2}C^\dagger)^\nu \tag{S110}$$

For the error compensation term, the gate cost shown in Eq. (27) has a saturation, which is upper bounded by $3n$. As analysed in Methods, the dominant cost is from the Trotterisation. For Hamiltonian in Eq. (7), one can use the fermionic swap network to simulate the Hamiltonian dynamics with depth $\mathcal{O}(n)$ and gate count $\tilde{\mathcal{O}}(n)$ in each segment. Using the results in Table S3, we can estimate the gate complexity for molecular systems in Eq. (7).

Corollary 1 (Eigenstate property estimation for molecular systems). *Suppose we aim to estimate the observable O on the eigenstate $|u_i\rangle$ of an n-qubit second-quantised plane wave Hamiltonian specified in Eq. (7), $\langle u_i|\hat{O}|u_i\rangle$. To achieieve an estimation error within ε, it is sufficient to have the gate count of $\tilde{\mathcal{O}}(n^2(\Delta^{-1})^{1+\frac{1}{4k+1}}\log(\eta^{-1}\varepsilon^{-1})\log(\vartheta^{-1}))$ and a success probability $1-\vartheta$.*

For quantum molecular systems, the Hamiltonian H takes the following form

$$H := \hat{T} + \frac{1}{2}\hat{V} + C = \sum_{i,j=1}^{n} h_{ij}\hat{a}_i^\dagger \hat{a}_j + \frac{1}{2}\sum_{i,j,k,l,=1}^{n} g_{ijkl}\hat{a}_i^\dagger \hat{a}_j^\dagger \hat{a}_k \hat{a}_l, \tag{S111}$$

where n is the number of spin orbitals of the molecular system; $\hat{a}_i^\dagger$ and $\hat{a}_i$ are the fermionic generation and annihilation operators, respectively; h_{ij} and g_{ijkl} are the corresponding coefficients for the one-body and two-body interactions, respectively. Note that the identity term in the Hamiltonian is a trivial term, so it is removed in the Hamiltonian in this work for simplicity. For quantum chemistry Hamiltonians, there has been considerable progress in efficiently representing the Hamiltonians with fewer terms and low weights.

These results, such as single factorisation [78] and double factorisation [49], can be directly applied to reduce the cost. A common strategy is to reformulate the two-body fermion operators as a sum of squared one-body operators by Cholesky decomposition, as has been used in AFQMC. The Hamiltonian is reformulated as

$$H = \hat{K} + \hat{V} := \hat{K} + \frac{1}{2}\sum_{\ell}^{\Gamma} \hat{L}_\ell^2, \tag{S112}$$

with $\hat{K} := \sum_{i,j=1}^{n}\left[h_{ij} - \frac{1}{2}\sum_{\ell=1}^{\Gamma}\sum_{k=1}^{n} L_{(ik)\ell}L^*_{(jk)\ell}\right]\hat{a}_i^\dagger \hat{a}_j$, and $\hat{L}_\ell := \sum_{i,l=1}^{n} L_{(il)\ell}\hat{a}_i^\dagger \hat{a}_l$. Here, the constant is removed and $\Gamma = \mathcal{O}(n)$ is the number of terms of $\hat{L}_\ell$.

Below, we use the first-order Trotter formula as an example to analyse the cost within each Trotter segment

$$e^{-iHt} \approx e^{-i\hat{K}t}\prod_{\ell=1}^{\Gamma} e^{-\frac{it}{2}U_\ell \sum_p (f_p\hat{n}_p)^2 U_\ell^\dagger} = e^{-i\hat{K}t}\prod_{\ell=1}^{\Gamma} U_\ell e^{-\frac{it}{2}\sum_p (f_p\hat{n}_p)^2} U_\ell^\dagger \tag{S113}$$

In the first line, Trotterisation is used, and thus, this is an approximation with some Trotter errors up to the second order. In the second line, some derivations have been abbreviated and the key facts that we used are $[U_\ell \hat{n}_p U_\ell^\dagger, U_\ell \hat{n}_q U_\ell^\dagger] = 0$ and $e^{-itU_\ell \hat{n}_p U_\ell^\dagger} = U_\ell e^{-it\hat{n}_p} U_\ell^\dagger$.

Using the idea in [75], the implementation of $e^{-\frac{it}{2}\sum_p (f_p\hat{n}_p)^2}$ may be done with $\mathcal{O}(n)$ depth circuit. Therefore in total, for each time segment, we require $\Gamma \times \mathcal{O}(n) = \mathcal{O}(n^2)$ depth circuit.

A very inefficient way to implement the above process may be like this. We first compute Z_j by applying a controlled NOT gate and store the information on the ancillary qubit $|j_1\rangle \dots |j_n\rangle$. Then implement as follows:

$$O_a \bigotimes_i |n_i\rangle |0\rangle_o \to \bigotimes_i |n_i\rangle |\sum_i f_i n_i\rangle \to \bigotimes_i |n_i\rangle |(\sum_i f_i n_i)^2\rangle \to \bigotimes_i |n_i\rangle e^{-i(\sum_i f_i n_i)^2} |(\sum_i f_i n_i)^2\rangle \tag{S114}$$

We require the following circuit oracle

$$O_a |j_1\rangle \dots |j_n\rangle |0\rangle_r = |j_1\rangle \dots |j_n\rangle |(-1)^{j_1} + (-1)^{j_2} + \dots + (-1)^{j_n}\rangle_r . \tag{S115}$$

$$O_{\vec{A}}|j\rangle|0\rangle_o|0\rangle_{\text{garb}} = |j\rangle |A_j\rangle_o |g(j)\rangle_{\text{garbage}} \tag{S116}$$

with $A_j = j^2$. Then

$$|j\rangle|0\rangle_o|0\rangle_{\text{garb}}|0\rangle \underset{O_{\vec{A}}}{\to} |j\rangle |A_j\rangle_o |g(j)\rangle_{\text{garb}}|0\rangle \underset{\text{PHASE}}{\to} e^{-iA_jt}|j\rangle |A_j\rangle_o |g(j)\rangle_{\text{garb}}|0\rangle \underset{O_{\vec{A}}^\dagger}{\to} e^{-iA_jt}|j\rangle|0\rangle_o|0\rangle_{\text{garb}}|0\rangle \tag{S117}$$

F. Effect of energy error on eigenstate property expectation estimation

In this section, we analyse the effect of energy error on eigenstate property expectation estimation. Our result indicates that as long as the energy estimation is ϵ-close to the true energy, the observable error can be bounded.

Suppose the energy has an estimation error $\kappa := |\hat{E}_j - E_j| \leq \Delta$. The observable expectation will become

$$\hat{O}_{\tau,x_c,s_c}(\hat{E}_j) = \frac{\hat{N}_{\tau,x_c,s_c}(O,\hat{E}_j)}{\hat{D}_{\tau,x_c,s_c}(\hat{E}_j)}. \tag{S118}$$

In the presence of estimation error $\kappa \neq 0$, $g_\tau(H-\omega)$ will tend to be zero $\|g_{\tau\to\infty}(H-\omega)\| = 0$. In such a case, we consider the projector $\hat{P}_i = |u_i\rangle\langle u_i|$. Eq. (S58) will need to be modified a little. For a general E, we have

$$g_\tau(H-\omega) = \sum_i g_\tau(E_i-\omega)\hat{P}_i. \tag{S119}$$

When $\tau \geq \frac{1}{\Delta}\sqrt{\ln(2/\varepsilon_\tau)}$, we have

$$\|g_\tau(H-\omega) - g_\tau(E_j-\omega)\hat{P}_j\| \leq \varepsilon_\tau/2 \tag{S120}$$

The error of the numerator can be bounded by

$$|N_\tau(O,E) - g_\tau^2(E_j-\omega)N(O)| \leq \varepsilon_\tau\|O\| \tag{S121}$$

Compared to the result with an accurate estimation, the only difference is that $N(O)$ and D is coupled with an additional factor $g_\tau^2(\kappa)$.

Recall that the objective is to estimate $N(O)$. Therefore, we put the factor $g_\tau^{-2}(\kappa)$ coupled with N_τ, and denote

$$\varepsilon_N := |g_\tau^{-2}(\kappa)N_\tau(O,E) - N(O)|. \tag{S122}$$

Since $g_\tau^{-2} > 1$, the error ε_N compared to the previous estimation error of the numerator is amplified.

The results concerning a finite cutoff and number of samples can be derived similarly to that in Theorem 5. More specifically, we have

$$\begin{aligned}
\left|\langle\hat{O}\rangle_{\tau,x_c,s_c} - \langle O\rangle\right| &= \left|\frac{\hat{N}_{\tau,x_c,s_c}(O,\omega)}{\hat{D}_{\tau,x_c,s_c}(\omega)} - \frac{N(O,\omega)}{D(\omega)}\right| \\
&= \left|\frac{D(\omega)\hat{N}_{\tau,x_c,s_c}(O,\omega) - N(O,\omega)\hat{D}_{\tau,x_c,s_c}(\omega)}{D(\omega)\hat{D}_{\tau,x_c,s_c}(\omega)}\right| \\
&\leq \frac{1}{|D(\omega)\hat{D}_{\tau,x_c,s_c}(\omega)|}\left(\left|D(\omega)\hat{N}_{\tau,x_c,s_c}(O) - \frac{\hat{N}_{\tau,x_c,s_c}(O,\omega)\hat{D}_{\tau,x_c,s_c}(\omega)}{g_\tau^2(\kappa)}\right|\right. \\
&\quad \left. + \left|\frac{\hat{N}_{\tau,x_c,s_c}(O,\omega)\hat{D}_{\tau,x_c,s_c}(\omega)}{g_\tau^2(\kappa)} - N(O,\omega)\hat{D}_{\tau,x_c,s_c}(\omega)\right|\right) \\
&\leq \left|\frac{D(\omega)\varepsilon_N(O,\omega) + N(O,\omega)\varepsilon_D}{g_\tau^2(\kappa)(D(\omega)^2 - D(\omega)\varepsilon_D)}\right| \\
&\leq \left|\frac{D(\omega)\varepsilon_N(O,\omega) + (N(O,\omega)+D(\omega))\varepsilon_D}{g_\tau^2(\kappa)D^2}\right| \\
&\leq g_\tau^2\eta^{-1}(2\|O\|+1)(\varepsilon_\tau+\varepsilon_c) + g_\tau^2\eta^{-1}(\|O\|+\|O\|_1+1)\varepsilon_n.
\end{aligned} \tag{S123}$$

Compared to the case with known eigenenergy, the observable estimation error ε will be amplified by a factor $g_\tau^{-2}(\kappa) = \exp(2\tau^2\kappa^2)$.

To ensure the estimation is nonvanishing, we require $\tau\kappa \leq c$, which indicates the energy precision needs to satisfy

$$\kappa \leq c\mathcal{O}(\Delta\log^{-1}((\eta\varepsilon)^{-1})). \tag{S124}$$

G. Discretised version

For the Gaussian-type spectral filter, we show a deterministic version of the LCU decomposition of the spectral filter in Eq. (9). Using the Gaussian integral, also known as the Hubbard-Stratonavich transformation which is widely used in field theories and auxiliary-field quantum Monte Carlo [35], we have

$$g_\tau(H) = e^{-\tau^2H^2} = \frac{1}{\sqrt{2\pi}}\int dx e^{-x^2/4}e^{-i\tau xH}. \tag{S125}$$

By converting the integral into a summation, we define a discretised version of $g_{\tau,x_c}^{(D)}(H-\omega)$ with a maximum evolution time x_c as

$$g_{\tau,x_c}^{(D)}(H-\omega) = \frac{1}{\sqrt{2\pi}} \sum_{j=-N_m}^{N_m} b e^{-x_j^2/4} e^{i\tau x_j (H-\omega)} \tag{S126}$$

with total number of steps N_m, the step size $b = x_c/N_m$, and $x_j = jb$, a superscript D denoting a discretised version. An infinite sum of $g_{\tau,x_c}^{(D)}(H-\omega)$ is given by

$$g_{\tau}^{(D)}(H-\omega) = \frac{1}{\sqrt{2\pi}} \sum_{j=-\infty}^{\infty} b e^{-x_j^2/4} e^{i\tau x_j (H-\omega)} \tag{S127}$$

with total number of steps N_m, the step size $b = x_c/N_m$, and $x_j = jb$.

The discretisation error can be bounded by

$$\varepsilon_{d,x_c} := |g_{\tau,x_c} - g_{\tau,x_c}^{(D)}| \leq |g_\tau - g_\tau^{(D)}| + |g_\tau - g_{\tau,x_c}| + |g_\tau^{(D)} - g_{\tau,x_c}^{(D)}| \tag{S128}$$

We define the discretisation error with an infinite expansion as $\varepsilon_d = |g_\tau - g_\tau^{(D)}|$ the truncation error $\varepsilon_{x_c} := |g_\tau - g_{\tau,x_c}|$ and its discretisation as $\varepsilon_{x_c}^{(D)} := |g_\tau^{(D)} - g_{\tau,x_c}^{(D)}|$

Since e^{-x^2} is a monotonic function, the discretisation form is less than the integral. From the definition of $g_{\tau,x_c}^{(D)}$ Eq. (S126), we can check that $\varepsilon_{x_c}^{(D)} \leq \varepsilon_{x_c}$. From Proposition 8, we know that $\varepsilon_{s_c} \leq \exp(-x_c^2/2)$ and thus $\varepsilon_{x_c}^{(D)}$ is bounded by $\exp(-x_c^2/2)$ as well. With the result derived in [14], ε_d can be bounded by

$$\varepsilon_d \leq \exp\left((\frac{2\pi}{b} - \tau)/2 \right)^2 \tag{S129}$$

For an equal distribution of error, we choose to set $\varepsilon_d = \varepsilon_{x_c} = \varepsilon/3$. When the stepsize is set as $b = 2\pi/(x_c+\tau) = \mathcal{O}(\Delta(\ln(\varepsilon^{-1}))^{-1/2})$, the total error is bounded by ε. The total number of steps is

$$N_m = x_c(x_c+\tau)/2\pi \leq \frac{2}{\pi\Delta} \ln\frac{2}{\varepsilon_\tau} = \mathcal{O}(\Delta^{-1}\ln(\varepsilon^{-1})) \tag{S130}$$

with ε_τ defined in Eq. (S100).

S4. EIGENENERGY ESTIMATION

In this section, we discuss the gate complexity of eigenenergy estimation described in Problem 2. We provide a proof for the second part of Theorem 1. We first discuss how to use the denominator to estimate the eigenenergy E_j. Intuitively, we can find that $D_\tau(\omega)$ indeed shows a coarse-grained energy spectrum. For the initial state $|\psi_0\rangle = \sum_i c_i |u_i\rangle$, the spectrum of the initial state can be characterised by

$$P(E) = \sum_i |c_i|^2 \delta(\omega - E_i). \tag{S131}$$

One can prove that $D_\tau(E) = [g_\tau^2 \star P](E)$ where $\star$ denotes the convolution of two functions.

Suppose we have a prior knowledge of $E_j \in [E_j^L, E_j^R]$. It is worth noting that we cannot distinguish eigenenergies that are very close to each other. The eigenenergies that are close to each other could be merged and regarded as a broadened eigenenergy. Here, we assume that the target resolution is less than the energy gap, i.e., $\kappa < \Delta$, and $E_j^R < E_j + \Delta/2$, $E_j^L > E_j - \Delta/2$. Given this range, the jth eigenenergy can be searched by

$$E_j = \operatorname*{arg\,max}_{\omega \in [E_j^L, E_j^U]} \hat{D}_\tau(\omega). \tag{S132}$$

The maximum of $D_\tau(\omega)$ within the range $[E_j^L, E_j^U]$ gives us an estimate of eigenenergy E_j.

In practice, we can only obtain an estimation $\hat{D}_\tau^{(x_c)}(E)$ of $D_\tau(E)$, when considering finite cutoff time x_c, segment number ν_c, truncation s_c and number of samples N_s. The eigenenergy is determined by

$$\hat{E}_j := \operatorname*{arg\,max}_{\omega \in [E_j^L, E_j^U]} \hat{D}_{\tau,x_c,s_c}(\omega). \tag{S133}$$

Similar to property estimation in Sec. S3, the error sources include a finite imaginary time, a finite cutoff of real-time evolution, discretization error, and the statistical error due to Hamiltonian simulation and finite number of samples

$$\hat{D}_{\tau,x_c,s_c}(\hat{E}_j) - D_\tau(E_j) \le \varepsilon_\tau + \varepsilon_{x_c} + \varepsilon_{s_c} + \varepsilon_n \tag{S134}$$

Based on the error dependence, we can estimate the resource requirements (i.e., circuit depth and number of samples) for eigenenergy estimation. Compared to Sec. S3, the only difference is that the denominator is a function of ω in energy estimation, while we take $\omega = E_j$ in property estimation in Sec. S3. The following lemma establishes the error due to a finite τ.

Lemma 2 (Error due to a finite τ (Proposition 3 in [22]))**.** *When $\tau \ge \frac{2}{\Delta}\sqrt{\ln(2/\varepsilon_\tau)}$, $|D_\tau(\omega) - \eta g_\tau^2(\omega - E_j)| \le \varepsilon_\tau$.*

The rest of the proof is nearly identical to Sec. S3. We give the result here.

Theorem 7 (Eigenenergy estimation for generic Hamiltonians)**.** *Suppose that $\hat{E}_j$ is determined by Eq. (S133) and the conditions and assumptions in Problem 2 hold.*

Case I: Suppose that we choose the time-segment number function $\nu_c = \mathcal{O}\left((\lambda\kappa^{-1}\ln(\eta^{-1}))^{1+\frac{1}{4k+1}}\right)$ for realising the real-time evolution using the method in Algorithm 1 (with the truncation order $s_c = \mathcal{O}(\ln(\nu_c)/\ln\ln(\nu_c)))$). We can achieve the error of eigenenergy estimation within κ, $|\hat{E}_j - E_j| \le \kappa$ with a success probability at least $1-\vartheta$ when the number of samples is $N_s = \mathcal{O}\left(\eta^{-2}\ln(1/\vartheta)\right)$.

Case II: When we choose the time-segment number function $\nu_c = \mathcal{O}\left((\lambda\Delta^{-1}\ln(\eta^{-1}\kappa^{-1}))^{1+\frac{1}{4k+1}}\right)$ for realising the real-time evolution, one can achieve the error of eigenenergy estimation within κ, $|\hat{E}_j - E_j| \le \kappa$ with a success probability at least $1-\vartheta$ when the number of samples is $N_s = \mathcal{O}\left(\eta^{-2}\Delta^4\kappa^{-4}(\ln(\kappa^{-2}\eta^{-1}))^2\ln(1/\vartheta)\right)$.

Proof. We start by proving the result in Case I. From Eq. (S133), we have

$$\hat{D}_{\tau,x_c,s_c}(\hat{E}_j) \ge \hat{D}_{\tau,x_c,s_c}(E_j). \tag{S135}$$

Then we bound the difference between $D_\tau(\omega)$ and $\eta g_\tau^2(\omega - E_j)$ using Lemma 2. That is, when we set the imaginary-time as $\tau \ge \frac{2}{\Delta}\sqrt{\ln\frac{2}{\eta\varepsilon_\tau}}$ we have

$$|D_\tau(E_j) - \eta| \le \eta\varepsilon_\tau,\ |D_\tau(\hat{E}_0) - \eta g_\tau(\hat{E}_0 - E_0)^2| \le \eta\varepsilon_\tau. \tag{S136}$$

Here it is worth noting that we do not have to know the value of η.

The maximum real-time is

$$t_c = \tau x_c = \frac{4}{\Delta}\sqrt{\ln(2/\eta\varepsilon_\tau)}\sqrt{\ln(2/\eta\varepsilon_c)}. \tag{S137}$$

According to Proposition 8, when the segment number is set by

$$\nu_c = 4\left(\frac{2(e+c_k)}{\ln 2}\right)^{\frac{1}{4k+1}}(\lambda\tau x_c)^{1+\frac{1}{4k+1}} \tag{S138}$$

$U_{2k}^{(s_c)}(t_i)$ is a $(2,\varepsilon_{s_c})$-LCU formula of $U(t_i)$ with the approximation error given by $\max_{t_i}\|U_{2k}^{(s_c)}(t_i) - U(t_i)\| \le \varepsilon_{s_c}$, and we have $|D_{\tau,x_c,s_c}(\omega) - D_\tau(\omega)| \le \eta\varepsilon_c$ with $\varepsilon_c = 3(\varepsilon_{x_c} + \varepsilon_{s_c})$

According to Proposition 10, when the number of measurements $N_s = 32\eta^{-2}\varepsilon_n^{-2}\ln(1/\vartheta)$, we have

$$\begin{aligned}\left|D_\tau(E_j) - \hat{D}_{\tau,x_c,s_c}(E_j)\right| &\le \eta(\varepsilon_c + \varepsilon_n)\\ \left|D_\tau(\hat{E}_j) - \hat{D}_{\tau,x_c,s_c}(\hat{E}_j)\right| &\le \eta(\varepsilon_c + \varepsilon_n)\end{aligned} \tag{S139}$$

with a success probability of $1-\vartheta$. According to [22], the following inequality holds

$$|\hat{E}_j - E_j| \le \frac{1}{\tau}g_\tau^{-1}\left(\sqrt{1 - 2(\varepsilon_\tau + \varepsilon_c + \varepsilon_n)}\right). \tag{S140}$$

When we set the precision for each component

$$\varepsilon_\tau = \varepsilon_c = \varepsilon_n \le \frac{1}{6}(1 - e^{-1}) \le 0.1 \tag{S141}$$

and $\tau \geq \kappa^{-1}$, we can make sure that the energy estimation precision is no greater than κ. From the precision requirement Eq. (S141) and Eq. (S137), we require

$$t_c \geq \kappa^{-1} \ln(20\eta^{-1}) \tag{S142}$$

when $\kappa \leq \Delta/4$. Using Proposition 8, the segment number is set to be

$$\nu_c = 4\left(\frac{2(e+c_k)}{\ln 2}\right)^{\frac{1}{4k+1}} (\lambda\kappa^{-1}\ln(20\eta^{-1}))^{1+\frac{1}{4k+1}} = \mathcal{O}\left((\lambda\kappa^{-1}\ln(\eta^{-1}))^{1+\frac{1}{4k+1}}\right) \tag{S143}$$

and set s_c by Eq. (S95).

In [20], Wang et al. showed that when using a Gaussian filter, the precision dependence can be improved, where the eigenenergy is also determined by Eq. (S133). Compared to Case I, the key difference is that show that the distance $|D_\tau(\omega) - \eta|$ is modulated by the estimation error $\omega - E_j$. When we set the imaginary time

$$\tau = \frac{1}{0.9\Delta}\sqrt{\ln(20\kappa^{-2}\eta^{-1})} = \mathcal{O}(\Delta^{-1}(\ln(\kappa^{-2}\eta^{-1}))^{-\frac{1}{2}}) \tag{S144}$$

we can distinguish E_j from the others. The cutoff by x_c can be similarly obtained. Using Proposition 8, when the segment number is set to be

$$\mathcal{O}\left((\lambda\Delta^{-1}\ln(\eta^{-1}\kappa^{-1}))^{1+\frac{1}{4k+1}}\right) \tag{S145}$$

the eigenenergy error can be bounded by κ by using the results in [20]. Note that in [20], the error is $\varepsilon_n = \tau\kappa$. Thus, the number of samples

$$N_s = \mathcal{O}(\eta^{-2}\tau^{-4}\varepsilon^{-4}\ln(1/\vartheta)) = \mathcal{O}(\eta^{-2}\Delta^4\kappa^{-4}(\ln(\kappa^{-2}\eta^{-1}))^2\ln(1/\vartheta)) \tag{S146}$$

□

For eigenstate property estimation, the gate complexity depends on the energy gap Δ. For eigenenergy estimation, the total gate count nearly reaches the Heisenberg limit $\mathcal{O}(\kappa^{-(1+o(1))})$ [21, 22], which may not be directly related to Δ.

Given a Hamiltonian with parameters n, L, $\mathrm{wt}(H)$, and $\mathrm{wt_m}(H)$, the gate complexity of eigenenergy estimation can be similarly obtained by using Proposition 3.

S5. CIRCUIT COMPILATION AND GATE COST FOR BLOCK-ENCODING-BASED METHODS

A. Stage Setting

In this section, we briefly introduce and estimate the gate cost for block-encoding-based methods. Here we mainly discuss the cost based on the result by Google's team in [52] which is used in our numerical simulation. There are considerable progress in reducing the cost for block encoding. We will not introduce these advanced techniques which are not the main focus of this work.

To estimate the gate cost of each algorithm, we synthesize the circuits to CNOT gates, single-qubit Clifford gates and T-gates. The CNOT gate number is more important for a near-term application on a quantum computer with no or limited fault tolerance, while the T-gate number is more critical for a long-term application on a fully fault-tolerant quantum computer. In some subroutines of the above algorithms, a direct estimation of the T-gate number is hard to obtain. In this case, we first synthesize the circuits to CNOT gates, single-qubit Clifford gates and single-qubit Z-axis rotation gates $R_z(\theta)$. Then we estimate the T-gate number n_T using the $R_z(\theta)$ gate number n_{Rz}.

We consider the optimal ancilla-free gate synthesis algorithm in Ref. [81], which requires $3\log_2(1/\varepsilon) + \mathcal{O}(\log\log(1/\varepsilon))$ T-gates to approximate the $R_z(\theta)$ gate to a precision ε. Here, we set the gate synthesis error of each $R_z(\theta)$ ε_{CS} to be a small value compared to the total error. In practice, we should determine the resource overhead c_T based on the number of R_z gates in the quantum algorithm.

We remark that, if we are allowed to introduce extra ancillary qubits and entangling Clifford gates, one can further reduce the required T gates to

$$1.15\log_2(1/\varepsilon) + 9.2$$

using a repeat-until-success strategy proposed in [82]. However, this will introduce extra ancillary qubit requirements and more CNOT gate costs.

In our resource analysis, to streamline the comparison, we exclude the observable estimation error due to a finite sampling cost. We will focus on the circuit depth to achieve a certain level of accuracy of the RCLU formula. Here, we remark that while the RLCU method cannot prepare the state, it can effectively prepare the eigenstate at the level of expectation. To get the resource cost, the key component is to get the segment number ν_c, which is directly related to the maximum real evolution time t_c. The gate count for CNOT gates and single-qubit rotation gates can be obtained by using Proposition 3.

Let us define

$$\begin{aligned} n_L &:= \lceil \log_2 L \rceil \\ \Lambda &:= \max_l \alpha_l \end{aligned} \tag{S147}$$

In the standard block encoding procedure [64], the n-qubit Hamiltonian H is encoded in a $(n_L + n)$ -qubit unitary, select(H)

$$\text{select}(H) := \sum_{l=1}^{L} |l\rangle\langle l| \otimes H_l \tag{S148}$$

Denote

$$|G\rangle := \text{PREPARE}\, |0\rangle^{n_L} = \frac{1}{\sqrt{\lambda}} \sum_{l=1}^{L} \sqrt{\alpha_l} |l\rangle, \tag{S149}$$

then we have

$$H = \lambda(\langle G| \otimes I)\, \text{select}(H)(|G\rangle \otimes I) \tag{S150}$$

which indicates that H is block-encoded into select(H). Here, PREPARE encodes the amplitude into the state on the ancillary space, and it is also referred to as the amplitude-encoding unitary or PREPARE operation in the literature.

Reflection unitary R, which is $R := (I - 2|0\rangle\langle 0|) \otimes I$ where the operation $(I - 2|0\rangle\langle 0|)$ is defined on the ancillary space with dimension n_L.

B. Gate cost

1. *The index enumeration circuit*

We follow the circuit construction in Ref. [52] to build the amplitude encoding operation (denoted by PREP or $B(x)$) and controlled select operation C-select(H). A major gadget of both operations is the following operation,

$$\text{C-select}(X) = \sum_{a=0}^{1} |a\rangle\langle a| \otimes \sum_{l=1}^{L} |l\rangle\langle l| \otimes (X_l)^a\,, \tag{S151}$$

where $X_l \in \{I, X\}$ is a single-qubit Pauli operator. The value of X_l depends on the value of l stored in the classical register. We can regard C-select(X) as a simplified version of C-select(H), where $H = \sum_{l=1}^{L} X_l$ is a single-qubit Hamiltonian where X_l is either I or X, based on the storage in the classical register.

In Sec. III in Ref. [52], the authors construct a "sawtooth" circuit to realise the C-select(X) gate (which is called the indexed operation in the original paper). In the simplified circuit of C-select(X) in Fig. 7 in Ref. [52], we need $(L-1)$ computing AND operations, $(L-1)$ uncomputing AND operations, L control- X_l gates, and $(L-1)$ extra CNOT gates. Suppose that we decompose the computing and uncomputing AND operations based on Fig. 4 in Ref. [52], and synthesize all the gates to Clifford $+T$ gates. We present the following observation.

Observation 1 (Gate cost in the index enumeration circuit). *If we construct the index enumeration circuit* C-*select*(X) *defined in Eq. (S151) following the 'sawtooth' way in Ref. [52] and synthesize all the gates to* CNOT *gates, single-qubit Clifford gates and* T *gates, then we can realise* C-*select* (X) *using*

1. $(6L - 5)$ CNOT *gates;*
2. $(4L - 4)$ T *gates.*
3. $(2L - 2)$ *Hadamard gates.*

C. The amplitude encoding, select gates, and reflection gates

Now, we estimate the gate cost in amplitude encoding, select, and reflection operations. The amplitude-encoding unitary B realises the following transformation,

$$B|0\rangle = \sum_{l=1}^{L} \sqrt{\omega_l}|l\rangle\,|\text{temp}_l\rangle$$

where $\omega_l := \alpha_l/\lambda$, is the normalised amplitude of the Hamiltonian. Following Ref. [52], we assume that it is allowed to introduce temporary storage $|\text{temp}_l\rangle$ during the amplitude encoding. This will not cause problems as long as we finally disentangle the system $|\text{temp}_l\rangle$ during the implementation of $B^\dagger$.

The dominant subroutine of the PREPARE circuit is the SUBPREPARE circuit defined in Eq. (48) in Ref. [52], which realises the amplitude encoding to different orbitals, ignoring the spin information first. In our discussion, we first ignore the detailed structure of the Hamiltonian H with respect to different spins. In this case, we can treat SUBPREPARE circuit to be the PREPARE circuit. To realise the SUBPREPARE circuit, we use the method introduced in Sec. IIID in Ref. [52]. The basic idea is to first prepare ancillaries with uniformly distributed coefficients over indecies l and then use a pre-determined binary representation of a probability keep_l, to perform a controlled-swap on the amplitude register l and another predetermined amplitude location alt_l. With well-designed values of swap probability keep send swap location alt_l, we can use the circuit in Fig. 11 in Ref. [52] to realise the SUBPREPARE circuit.

Suppose we want to realise the amplitude encoding with an accuracy of ε_{AE}, that is, to realise the following transformation,

$$B^{\varepsilon_{AE}}|0\rangle = \sum_{l=1}^{L} \sqrt{\tilde{\omega}_l}|l\rangle\,|\text{temp}_l\rangle \tag{S152}$$

where $\tilde{\omega}_l$ is a n_{AE} bit approximation to the true value ω_l

$$|\tilde{\omega}_l - \omega_l| \leq \varepsilon_{AE}, \quad l = 1, \ldots, L.$$

The number of ancillary qubits is required to be $n_{AE} = \lceil -\log_2 \varepsilon_{AE} \rceil$.

Due to the relation of the rescaled spectrum by block encoding, we have $\varepsilon_{\text{PREPARE}} = \frac{\varepsilon}{\lambda}$. The relation of the amplitude encoding error and PREPARE error could be derived by considering the norm of the Hamiltonian, and a simple relation is given by $\varepsilon_{AE} \sim \frac{\varepsilon_{\text{PREPARE}}}{L}$. Following Ref. [52] (see Fig. 11), we need to introduce at least $2n_{AE} + 2n_L + 1$ extra ancillary qubits, and $n_L := \lceil \log_2 L \rceil$. To simplify the gate cost, we assume L is a power of 2. In this case, the first layer of the circuit in Fig. 11 in Ref. [52] can be realised using Hadamard gates. If L is not a power of 2, additional quantum resources are needed.

The second and the third layer of the circuit requires the QROM circuit in Fig. 10 in Ref. [52], which is a modified version of the index enumeration circuit C-select(X) defined in Eq. (S151).

Based on Observation 1, the second layer of date loading requires $5(L-1) + L(n_L + n_{AE})$ CNOT gates, $4(L-1)$ T gates. The third layer is a coherent inequality test, which requires $(n_{AE} - 1)$ AND and uncomputing AND operations, and additional $6(n_{AE} - 1)$ CNOT gates plus 1 Toffoli gate. Thus, it requries $11n_{AE} - 5$ CNOT gates and $4n_{AE} + 3$ T gates.

The fourth layer is a Fredkin gate, which is a controlled swap gate. Following Fig. 5 in [83], this gate can be synthesised into Clifford + T gates using $8n_L$ CNOT gates and $7n_L T$ gates.

Observation 2 (Ancillary qubit and gate costs in the second-type amplitude encoding operation)**.** *If we synthesize the $B^{\varepsilon_{AE}}$ unitary defined in Eq. (S152) to* CNOT *gates, single-qubit Clifford gates and T gates, then the approximate ancillary and gate cost of $B^{\varepsilon_{AE}}$ are listed as follows,*

1. $2n_{AE} + 2n_L + 1$ *extra ancillary qubits;*
2. $n_L(L+8) + n_{AE}(L+11) + 5L - 10$ CNOT *gates;*
3. $4(L + n_{AE}) + 7n_L + 3$ *T gates;*

Here, $n_{AE} := \lceil -\log_2 \varepsilon_{AE} \rceil$ and $n_L := \lceil \log_2 L \rceil$.

With the above result, it is easy to analyse the gate cost of the C-select(H) gate. A straightforward implementation of the C-select(H) gate is to replace of X_l gate in C-select(X) defined in Eq. (S151) to multi-qubit Pauli gates P_l. For instance, consider the transverse field Ising model

$$H = J\sum_i \sigma_i^z\sigma_{i+1}^z + h\sum_i \sigma_i^x \tag{S153}$$

with the periodic boundary condition. The gate cost for the lattice Hamiltonian is shown in Corollary 2.

Corollary 2 (Gate cost in the C-select(H) operation of the lattice model)**.** *If we construct the controlled-select circuit* C-select(H) *of the lattice model following the 'sawtooth' way in Ref. [52] and synthesize all the gates to* CNOT *gates, single-qubit Clifford gates and T gates, then the approximate gate cost of* C-select(H) *is listed as follows,*

1. $5(L-1)+\mathrm{wt}(H)$ CNOT *gates;*
2. $(4L-4)$ T *gates.*

Next, we consider the gate cost for the fermionic Hamiltonian in Eq. (S111). The fermionic Hamiltonian can be mapped to a qubit form by JW transformation. We suppose there are L terms with distinctive coefficients in total. To further improve the gate cost in a fermionic Hamiltonian, Ref. [52] introduces an accumulator during the Pauli gate query process (Sec. IIIB and Fig. 8 in Ref. [52]). The accumulator will 'accumulate' the effect of the Pauli operators accessed in the previous data queries and save the CNOT gate cost. Using this improved select operation, we can reduce the CNOT cost for each Pauli operator P_l to a constant independent of the weight of P_l. We will use an optimistic estimate of the CNOT gate cost for controlled-P_l operations, which is $5(L-1)+3L=8L-5$ for QSP in the numerical simulation. The actual cost should be greater than this value.

Observation 3 (Ancillary qubit and gate costs in the reflection operation [84])**.** *If we construct the reflection operation* $I-2|0\rangle\langle 0|$ *on n qubits following the methods in Proposition 4 in [84] and synthesise all the gates to* CNOT *gates, single-qubit Clifford gates and T gates, then the approximate ancillary qubit and gate costs are listed as follows,*

1. $\lceil \frac{n-3}{2} \rceil$ *ancillary qubits*
2. $(6n-12)$ CNOT *gates;*
3. $(8n-17)$ T *gates.*

D. Gate cost for Trotter methods

In the Trotter methods, we first divide the real-time evolution into ν segments,

$$e^{-iHt} = \left(e^{-iHx}\right)^{\nu}. \tag{S154}$$

where $x := t/\nu$. The first-order Trotter formula is

$$S_1(x) = \prod_{l=1}^{L} e^{-ixH_l}. \tag{S155}$$

and the second-order Trotter formula is

$$S_2(x) = \prod_{l=L}^{1} e^{-i(x/2)H_l}\prod_{l=1}^{L} e^{-i(x/2)H_l}. \tag{S156}$$

The $(2k)$th-order Trotter formula is

$$S_{2k}(x) = \left[S_{2k-2}\left(p_k x\right)\right]^2 S_{2k-2}\left(\left(1-4p_k\right)x\right)\left[S_{2k-2}\left(p_k x\right)\right]^2 \tag{S157}$$

with $p_k := 1/\left(4-4^{1/(2k-1)}\right)$ for $k \geq 1$

We use the results for $2k$th-order Trotter formula from [58, 85] to analyse the Trotter Cost. We put their results below for the ease of readers.

Lemma 3 (Simple Trotter error bound for the $2k$th-order Trotter formula ([58, 85])). *Let $H = \sum_{l=1}^{L} H_l$ be a Hamiltonian consisting of L summands and $t \geq 0$. We denote*

$$a_{2k}(\nu) := 2\frac{\left(2\cdot 5^{k-1}L\Lambda t\right)^{2k+1}}{(2k+1)!\nu^{2k+1}}e^{2\cdot 5^{k-1}L\Lambda t/\nu}, b_{2k}(\nu) := \frac{L^{2k}\left(2\cdot 5^{k-1}\Lambda t\right)^{2k+1}}{(2k-1)!\nu^{2k+1}}e^{2\cdot 5^{k-1}L\Lambda t/\nu}, \tag{S158}$$

where $k \geq 1, \nu$ is the time segment number, Λ is defined in Eq. (2). If we set the segment number ν to be

$$\nu_{2k}^{\det} = \min\left\{\nu \in \mathbb{N} : \frac{\nu}{2}a_{2k}(\nu) \leq \varepsilon\right\} \tag{S159}$$

for the deterministic Trotter formula, or set ν to be

$$\nu_{2k}^{random} = \min\left\{\nu \in \mathbb{N} : \frac{\nu}{2}\left(a_{2k}(\nu)^2 + 2b_{2k}(\nu)\right) \leq \varepsilon\right\}, \tag{S160}$$

for the randomised Trotter formula, then the spectral norm distance of the resulting simulation channel to the unitary channel of e^{-iHt} is at most ε.

From Eq. (S159), the time segment can be roughly approximated by

$$\nu_{2k} \leq \frac{\left(2\cdot 5^{k-1}L\Lambda t\right)^{1+\frac{1}{2k}}}{((2k+1)!)^{\frac{1}{2k}}\varepsilon^{\frac{1}{2k}}}. \tag{S161}$$

In our numerical simulation, we search the required segment number by Eq. (S159).

S6. GROUND STATE PROPERTY ESTIMATION BY QUANTUM SIGNAL PROCESSING PROPOSED BY LIN AND TONG

A. Overview

In this section, we first review the key ingredient of the seminal algorithm proposed by Lin and Tong [28]. Their method relies on the block encoding of a non-unitary matrix in the quantum circuit. To establish a clear connection to [28] and facilitate the reader, we will follow the notation and conventions used in [28].

To simplify the notations, we denote the CNOT gate and T gate required for select(H) operation as S_{CNOT} and S_{T}, and the CNOT gate and T gate required for PREPARE operation as P_{CNOT} and P_{T}, respectively.

A matrix $A \in \mathbb{C}^{N\times N}$ where $N = 2^n$ can be encoded in the upper-left corner of an $(n_L + n)$-qubit unitary matrix if

$$\|A - \alpha(\langle 0^{n_L}| \otimes I)U(|0^{n_L}\rangle \otimes I)\|_2 \leq \varepsilon. \tag{S162}$$

and we refer to U as an $(\alpha, n_L, \varepsilon)$-block-encoding of A. In this work, we consider the Hamiltonian written in an LCU form in Problem 1. In the standard block encoding procedure, the n-qubit Hamiltonian H can be explicitly block-encoded into $U_H := \text{PREPARE}^\dagger \cdot \text{select}(H) \cdot \text{PREPARE}$, as shown in Eq. (S150).

The state preparation algorithm based on QSP is summarised below.

1. Obtain the $(\lambda, n_L, 0)$-block-encoding of a Hermitian matrix $H = \sum_k E_k |\psi_k\rangle\langle\psi_k| \in \mathbb{C}^{N\times N}$, $N = 2^n$, $\mathbb{E}_k \leq \mathbb{E}_{k+1}$. This block encoding is constructed by U_H.

2. Construct a $(\lambda + |\mu|, n_L + 1, 0)$-block-encoding of matrix $H - \mu I$ using of [42, Lemma 29] for any $\mu \in \mathbb{R}$.

3. Construct an $(1, n_L + 2, \varepsilon)$-block-encoding of

$$R_{<\mu} = \sum_{k:\mathbb{E}_k<\mu} |\psi_k\rangle\langle\psi_k| - \sum_{k:E_k>\mu} |\psi_k\rangle\langle\psi_k|.$$

   This is realised by constructing a block encoding of the sign function $-S(\frac{H-\mu I}{\alpha+|\mu|}; \delta, \varepsilon)$ for any δ and ε where $S(\cdot, \delta, \varepsilon)$ is the sign function of degree $d = \frac{e}{2\delta}\ln(32\pi^{-1/2}\varepsilon^{-1})$. Note that if we assume further that $\Delta/2 \leq \min_k |\mu - \mathbb{E}_k|$, then we let $\delta = \frac{\Delta}{4\lambda}$, all the eigenvalues of $-S(\frac{H-\mu I}{\lambda+|\mu|}; \delta, \varepsilon)$ are ε-close to either -1 or 1, and thus $-S(\frac{H-\mu I}{\lambda+|\mu|}; \delta, \varepsilon)$ is ε-close, in operator norm, to the reflector about the direct sum of eigen-subspaces corresponding to eigenvalues smaller than μ:

4. Using the block encoding of $R_{<\mu}$, we can construct an $(1, n_L+3, \varepsilon/2)$ block encoding of the projection operator $P_{<\mu} := \frac{1}{2}(R_{<\mu} + I)$.

5. Obtain the ground state with a success probability close to 1 by amplitude amplification.

6. Observable estimation.

B. Gate count and depth analysis for QSP and QETU

Next, we show the resource analysis of each step when compiling into elementary gates. We denote the resource as $(\cdot,\cdot,\cdot,\cdot)$ with the four elements $(\cdot)$ representing the ancillary qubits, the number of CNOT gates, the number of T gates, and single-qubit R_z rotations.

1. Block encoding: $(n_L, S_{\text{CNOT}} + 2P_{\text{CNOT}},\ S_{\text{T}} + 2P_{\text{T}}, 0)$

2. Controlled select(H) and two PREPARE operations: $(n_L+1, S_{\text{CNOT}} + 2P_{\text{CNOT}},\ S_{\text{T}} + 2P_{\text{T}}, 2)$

3. QSP of the sign function and hence the R operator: $(n_L + 2, d(S_{\text{CNOT}} + 2P_{\text{CNOT}}) + 2d, d(S_{\text{T}} + 2P_{\text{T}}), 3d)$ with $d = [\frac{2e\lambda}{\Delta}\ln(32\pi^{-1/2}\varepsilon^{-1})]$ obtained from Lemma 4

4. Projector, which is a controlled version of R: $(n_L+3, d(4+6S_{\text{CNOT}}+2P_{\text{CNOT}}+2S_{\text{T}}+2L), d(7S_{\text{CNOT}}+5S_{\text{T}}+2P_{\text{T}}+4L), 4d)$.

5. Amplitude amplification. $(n_L+3+\lceil\frac{n-3}{2}\rceil, d\gamma^{-1}(S_{\text{CNOT}}+2P_{\text{CNOT}}+6n-10), d\gamma^{-1}(S_{\text{T}}+2P_{\text{T}}+8n-17), 3d\gamma^{-1})$ with $d = [\frac{2e\lambda}{\Delta}\ln(32\pi^{-1/2}\gamma^{-1}\varepsilon^{-1})]$.

In the 4th step. In each block, the controlled gates:

1. Controlled Phase iterate: 2 CNOT + 2 single-qubit rotation. Thus, the total single-qubit Pauli rotation gate is $4d$.

2. Controlled select(H) and 2 PREPARE. CNOT: $6S_{\text{CNOT}} + 2P_{\text{CNOT}} + 2S_{\text{T}}$. The thrid $2S_{\text{T}}$ is from that one controlled T gate can be synthesised by 2 CNOT and 2 $\sqrt{\text{T}}$ gates.

   T gate: $7S_{\text{CNOT}}+5S_{\text{T}}+2P_{\text{T}}$, where the first 7 comes from Toffolis gates, the second 5 is from that one controlled T gate can be synthesised by 2 CNOT and 2 $\sqrt{\text{T}}$ gates, and we simply assume that 2 $\sqrt{\text{T}}$ may be catalysed by 5 T gats using Hamming weight by [50].

   Note that the select(H) has the Hadamard gates: each controlled Hadamard gives 2 T gates and 1 CNOT gate. At least, we have $2L + n_L$ Hadamard gates in select(H).

3. The other operations are symmetric.

In total, QSP requires the number of CNOT gates

$$d(2+2+6S_{\text{CNOT}}+2P_{\text{CNOT}}+2S_{\text{T}}+2L)$$

T gates

$$d(7S_{\text{CNOT}}+5S_{\text{T}}+2P_{\text{T}}+4L)$$

In the 5th step, the additional cost is from the reflection from Observation 3.

The ground state preparation error is composed of two parts: the error from the approximation of the sign function, and the block-encoding error of the PREPARE operation

$$\varepsilon = \varepsilon_{sgn} + \varepsilon_{tot,\text{PREPARE}}. \tag{S163}$$

Considering the $2d$ repetition of the PREPARE operation and the relation indicated by, we have

$$\varepsilon_{\text{PREPARE}} = \frac{\varepsilon}{4d\lambda} \tag{S164}$$

we choose to set the amplitude encoding error as

$$\varepsilon_{AE} \sim \frac{\lambda}{L}\varepsilon_{\text{PREPARE}} = \frac{\varepsilon}{4Ld} \tag{S165}$$

and $n_{AE} = \lceil -\log_2 n_{AE} \rceil$.

A key component in [28] is a polynomial approximation of the sign function in the domain $[-1, -\delta] \cup [\delta, 1]$. To derive the actual degree, we use an explicit construction of a polynomial with the same error scaling provided in [75] based on the approximation of the *erf* function.

Lemma 4 (Polynomial approximation to the sign function sgn(x) (QETU overhead) [75])**.** *For any $\delta < 1$, $\varepsilon \leq \sqrt{2/\pi e}$, the polynomial $S(x, \delta, \varepsilon) = p_{sgn,\delta,n}(x) = p_{erf,k,n}(x)$ of odd degree $d = \left[\frac{e}{2\delta}\ln(32\pi^{-1/2}\varepsilon^{-1})\right] = \mathcal{O}(\delta^{-1}\log(\varepsilon^{-1}))$ satisfies*

$$\varepsilon_{sgn,\delta,n} = \max_{x\in[-1,-\delta]\cup[\delta,1]} |p_{sgn,\delta,n}(x) - sgn(x)| \leq \varepsilon. \tag{S166}$$

The actual gate cost for the QETU method presented in this work is analysed by Lemma 4.

It is also interesting to note why QETU may not be able to run the real-time evolution in parallel when the qubit connectivity is restricted to nearest-neighbours. The QETU method requires one ancillary qubit because of the phase iteration. For certain Hamiltonians, if there exists a single Pauli operator K_j such that it anticommutes with each component of H, then the evolution can be implemented in a control-free way. For example, for Heisenberg models, we can divide the Hamiltonian into three terms $H = H_X + H_Y + H_Z$ where H_X, H_Y, H_Z contain tensor products of Pauli X, Y, Z, respectively. For H_X, we can choose $K_1 = \otimes_{i\in\text{odd}} X_i \otimes_{i\in\text{even}} Y_i$. However, the controlled-K operations still need depth $d_K = \mathcal{O}(n)$, as opposed to $d = \mathcal{O}(1)$ in our ancilla-free scheme. For electronic structure problems, to reduce the Trotter error and implement the operations in parallel, we can group the Hamiltonian into $\hat{T}$ and $\hat{V}$, in which case it is difficult to find a Pauli operation K such that it commutes with the grouped term. If the Hamiltonian is not grouped, then the circuit depth will be increased to $d = \mathcal{O}(n^3)$ when implementing each individual term in a naive way.

S7. GROUND STATE PROPERTY ESTIMATION WITH PHASE ESTIMATION

A. Complexity of phase estimation

For the canonical QPE algorithm, we apply a series of controlled U, U^2, ..., U^{2^k-1} and an inverse quantum Fourier transform on the ancillary k qubits, such that the state becomes

$$\sum_i c_i |0^{\otimes k}\rangle |E_i\rangle \to \sum_i p_i |\text{bin}(E_i)\rangle |E_i\rangle . \tag{S167}$$

To obtain a binary estimate of the energy precise to $n = \lceil \log_2 \varepsilon^{-1} \rceil$ bits, we require $k = \mathcal{O}(\log_2 \varepsilon^{-1} + \log_2 \eta^{-1/2})$ ancillary qubits [55]. The coherent runtime for each phase estimation is $2^{k+1}\pi = \mathcal{O}(\varepsilon^{-1}\eta^{-1/2})$, and the number of calls to phase estimation is $\mathcal{O}(\eta^{-1/2})$. The total gate complexity is

$$\mathcal{O}(C_{gate}\eta^{-1}\varepsilon^{-1}) \tag{S168}$$

where C_{gate} is the gate cost within each segment.

To obtain a binary estimate of the energy precise to $n = \lceil \log_2 \varepsilon^{-1} \rceil$ bits, we require $k = \mathcal{O}(\log_2 \varepsilon^{-1} + \log_2 \eta^{-1/2})$ ancillary qubits. The total error is composed of following parts: the error of phase estimation, Hamiltonian simulation, and circuit synthesis.

$$\varepsilon_{\text{tot}} = \varepsilon_{PE} + \varepsilon_{HS} + \varepsilon_{CS}$$

The coherent runtime for each phase estimation is lower bounded by

$$t_{PE}^{En} = \frac{\pi}{2\eta\varepsilon_{PE}} \tag{S169}$$

In the following, we will discuss the Hamiltonian simulation

Lemma 5 (Ground state preparation with phase estimation for known ground energy)**.** *Using the canonical phase estimation, the state can be prepared* ε *close to ground state using* $k = \mathcal{O}(\log_2 \varepsilon^{-1} + \log_2 \Delta^{-1} + \log_2 \eta^{-1/2})$*. The runtime for each phase estimation is* $2^{k+1}\pi = \mathcal{O}(\varepsilon^{-1}\Delta^{-1}\eta^{-1/2})$*, and the number of calls to phase estimation is* $\mathcal{O}(\eta^{-1/2})$ *using fixed point search. The total gate complexity is* $\mathcal{O}(\frac{C_{gate}}{\eta^2\Delta\varepsilon})$*.*

The coherent runtime for each phase estimation is lower bounded by

$$t_{PE}^{prep} = \frac{\pi}{2\Delta\varepsilon_{PE}} \tag{S170}$$

The error of estimation of an observable O consists of the following components:

$$\varepsilon = \varepsilon_{PE} + \varepsilon_{HS} + \varepsilon_{CS} + \varepsilon_{observ}.$$

The error of estimating observables using N_s samples is given by

$$\varepsilon_{observ} = \frac{C_{observ}}{\sqrt{N_s}} \tag{S171}$$

Suppose we use the importance sampling to estimate the observable $O = \sum_l o_l P_l$, the measurement overhead is $C_{observ} = \|O\|_1$, and we may use other methods to reduce C_{observ}, such as Pauli grouping or classical shadow methods to reduce the cost. In our numerical simulation, we use an optimistic estimation by only considering the dominant cost from the last operations $C - U^{2^{k-1}}$ only, and neglect the cost by controlled operations. The gate cost for QFT is neglected as well, which scales as $\mathcal{O}(k)$.

B. Hamiltonian simulation by Trotterisation

The overall circuit complexity for $2k$ order achives minima when $\varepsilon_{PE} = \varepsilon_{HS} = \varepsilon/2$

$$\frac{\left(\pi \cdot 5^{k-1} L\Lambda\eta^{-1}\right)^{1+\frac{1}{2k}}}{((2k+1)!)^{\frac{1}{2k}}\varepsilon_{PE}^{1+\frac{1}{2k}}\varepsilon_{HS}^{\frac{1}{2k}}} \tag{S172}$$

Its minimum is obtained at

$$\varepsilon_{PE} = \frac{2k+1}{2(k+1)}\varepsilon,\ \varepsilon_{HS} = \frac{1}{2(k+1)}\varepsilon$$

Ground state energy estimation with phase estimation + higher-order Trotter:
Gate count for eigenenergy estimation.

1. Get the runtime $t_{PE}/2$ with $\varepsilon_{PE} = \varepsilon/2$ in Eq. (S169).
2. Determine the number of segment ν using Lemma 3.
3. CNOT gates: $2 \cdot 5^{k-1}\nu\eta^{-1/2}(2\,\mathrm{wt}(H) - L + 2)$.
4. Single-qubit Z-axis Pauli rotation gate: $4 \cdot 5^{k-1}\nu\eta^{-1/2}L$.

Gate count for eigenstate property estimation.

1. Get the measurement overhead C_{observ}, runtime $t_{PE}/2$ as a function of ε_{observ} and ε_{PE}, respectively.
2. Determine the number of segment ν using Lemma 3 as a function of ε_{HS}. An approximation of ν is given by Eq. (S161).
3. CNOT gates: $2 \cdot 5^{k-1}\nu\eta^{-1/2}C_{observ}\varepsilon_{observ}^{-2}(2\,\mathrm{wt}(H) - L + 2)$.
4. Single-qubit Z-axis Pauli rotation gate: $4 \cdot 5^{k-1}\nu C_{observ}\varepsilon_{observ}^{-2}\eta^{-1/2}L$.
5. Get the gate count by optimising over the distribution of ε.

C. Hamiltonian simulation by qubitised quantum walk

The phase estimation combining the qubitisation methods has been discussed in [52], which is compared to other algorithms. In the following, we will review the method before analysing the resource costs. The key idea of qubitised quantum walk is that the spectrum of H can be obtained by performing phase estimation on the Szegedy quantum walk operator, defined as

$$\mathcal{W} := (2\left|G\right\rangle\left\langle G\right| \otimes I - I) \cdot \mathrm{select}(H) \tag{S173}$$

with $|G\rangle = \textsc{prepare}\,|\bar{0}\rangle$. The spectrum has a relation spectrum$(H) = \lambda\cos(\arg[\mathrm{spectrum}(\mathcal{W})])$ with $\arg(e^{i\phi}) = \phi$. Their results suggest that we can estimate the phase to a number of bits given by

$$k = \left\lceil \log \left(\frac{\sqrt{2}\pi\lambda}{2\varepsilon_{PE}} \right) \right\rceil \tag{S174}$$

with k extra ancillary qubits. Here, we further assume a small error of gate synthesis in PREPARE and QFT. Using phase estimation, the query number is

$$d := 2^k \leq \frac{\sqrt{2}\pi\lambda}{2\varepsilon_{PE}} + 1. \tag{S175}$$

The state preparation error $\varepsilon_{\textsc{prepare}}$ for a single application of $\mathcal{W}$ is

$$\varepsilon_{tot,\textsc{prepare}} \leq \| e^{i\arccos(H/\lambda)} - e^{i\arccos(\tilde{H}/\lambda)} \| \tag{S176}$$

It is related to the amplitude encoding error ε_{AE} by

$$\varepsilon_{\textsc{prepare}} \leq \frac{L\varepsilon_{AE}}{\lambda} \left(1 - \left(\frac{\|H\| + L\varepsilon_{AE}}{\lambda} \right)^2 \right)^{-1/2} \tag{S177}$$

Suppose that we require the preparation error to be $\varepsilon_{\textsc{prepare}}$. One can choose to set

$$\varepsilon_{AE} = \frac{\varepsilon_{\textsc{prepare}}}{(1 + \varepsilon^2_{\textsc{prepare}})L} \tag{S178}$$

assuming that $\Omega(\frac{\|H\|}{\lambda}) = 0$. The preparation error is set to be

$$\varepsilon_{\textsc{prepare}} \leq \frac{\sqrt{2}}{2\lambda} \frac{\varepsilon_{PE}}{2^k} = \frac{\sqrt{2}\varepsilon_{PE}}{\lambda 2^{k+1}} \tag{S179}$$

and hence for a single block

$$\varepsilon_{AE} = \frac{\sqrt{2}\varepsilon}{4L\lambda d}. \tag{S180}$$

Note that 2 PREPARE is used in one block. By Eq. (S178), we can determine the amplitude encoding error ε_{AE} as $\varepsilon_{AE} = \frac{\varepsilon^2_{PE}}{\pi L\lambda}$. Again, the cost from the QFT is ignored, which scales as $\mathcal{O}(k\log k)$.

The overall gate complexity of the eigenenergy estimation is $\mathcal{O}(\frac{\lambda L}{\varepsilon})$. The total gate count can be estimated by using Observation 2, Observation 3, and the Hamiltonian dependent select(H), given by Corollary 2.

Each block requires: 1 controlled select(H), 2 PREPARE and 1 Reflection on n_L qubits, which has the gate count

$$(n_L + \max(k, n_L + 2n_{AE} + 1), S_{\mathrm{CNOT}} + 2P_{\mathrm{CNOT}} + 6(n_L - 2), S_{\mathrm{T}} + 2P_{\mathrm{T}} + (8n_L - 17), 0). \tag{S181}$$

and an additional k repetition of controlled reflection, each block (2 preparation) has the cost:

$$(0, 2P_{\mathrm{CNOT}} + 6(n_L - 1), 2P_{\mathrm{T}} + (8n_L - 9), 0) \tag{S182}$$

In total d queries and $\eta^{-1}\Delta^{-1}$ repetitions are required, which results in

$$\begin{aligned} (n_L + \max(k, (n_L + 2n_{AE} + 1), &\eta^{-1}\Delta^{-1}\left(d(S_{\mathrm{CNOT}} + 2P_{\mathrm{CNOT}} + 6(n_L - 2)) + k(2P_{\mathrm{CNOT}} + 6(n_L - 1))\right), \\ &\eta^{-1}\Delta^{-1}\left(d(S_{\mathrm{T}} + 2P_{\mathrm{T}} + (8n_L - 17)) + k(2P_{\mathrm{T}} + (8n_L - 9))\right), 0). \end{aligned} \tag{S183}$$

S8. INVESTIGATION ON THE RESOURCE COST

A. Numerical setting

In this section, we first present the details of the numerical simulation. We will also present additional resource estimation results.

To estimate the gate costs of the RLCU algorithm, in this work we set the normalisation factor $\mu = 2$ to ensure that the sample complexity of the RLCU algorithm is similar to other quantum algorithms. The operations involved in the algorithms are CNOT gates and single-qubit Pauli rotation gates $R_z(\theta)$, which is further decomposed into T gates. The circuit compilation overhead is detailed in Supplementary Sec. S5.

Energy gap fitting For the Heisenberg type of Hamiltonian with the additional field on the boundary in Eq. (8) in the main text, when $h_x = 0$, it has a constant gap $\Delta(c) = 4(c-1)$ when the system size is infinite. The energy degeneracy is $n+1$. For example, for $c = 2$, it has a constant energy gap $\Delta = 4$.

When the external field h_x increases, more excited states will emerge. However, we find by numerical fitting that for $n \leq 100$, the energy gap is not very small. We find that the energy gap can be well fitted by a polynomial function. In Fig. S1, we show the fitting by $\Delta = b \cdot n^a$ with $a = -0.50$, which agrees quite well with the actual gap at small system sizes. In contrast, the energy gap fitting by an exponential function $\Delta = b \exp(-an)$ does not agree well. The gap dependence has been considered in our resource estimation.

Remark on the initial state. As the central objective is to reduce the depth, in resource estimation, we mainly focus on the maximum gate count in a single run, whose scaling is logarithmic in initial state overlap η. In other words, this maximum gate count that needs to be implemented coherently is nearly independent of η. In contrast, the standard phase-estimation procedure will have a worse gate complexity dependent on η, $\mathcal{O}(\eta^{-1}\epsilon^{-1})$. To make a fair comparison with other methods, we set the initial state overlap to be a constant value as similarly used in [12, 17]. For instance, η is set to be 1 for quantum chemistry example FeMoco in [17], and $\eta = 0.8$ for Ising models in [12]. In [49] they used DMRG to find the ground state with η around 0.9. In [21] they have used a Hartree-Fock state for a 8-site Fermi-Hubbard model with η around 0.4. On the other hand, we have highlighted in the paper that this will mostly affect the sampling numbers, the sampling complexity has a similar dependence (in Theorems 5 and 7) to other selected randomised works whose practical performance is good (i.e. with few actual gate counts). We also note that the sampling overhead due to randomisation is included in all the plots in the resource estimation to ensure that this method is compared with other deterministic algorithms like QSP at the same level.

In a way, initial state preparation can be improved by using methods like adiabatic evolution and dissipative method as well as various physics-inspired or MPS-based methods. The paper suggested by the referee is very helpful in this context. The key is to employ MPS techniques for state preparation. This work focuses on how to obtain suitable initial states and is fully compatible with our approach, i.e., one can use the MPS-based strategy to prepare an appropriate initial state, and then apply the method developed in our work to estimate eigenenergies and eigenstate properties with high precision.

The requirements for the gate number with the selected advanced algorithms are estimated. Since the central objective is for the application in the early FTQC or NISQ era, we mainly focus on the circuit depth in a single-run experiment. Therefore, the amplitude amplification is not considered in the algorithms, which can deterministically prepare the state closer to the true ground state yet at the cost of a deeper circuit. When we consider a real physical model, such as quantum chemistry problems, the coefficient of the Hamiltonian is constructed by calculating the integral and represents the feature of the quantum system. Due to finite precision, there will be an amplitude encoding error when we perform the PREPARE operation. To ensure that the amplitude encoding error in the block encoding procedure is less than a threshold, we require more qubits to encode the coefficient. However, for the toy models, the absolute value of the coefficient may not be essentially relevant for the actual physics. For instance, we can manually set the interaction strength when we study the phase transition. In this work, we include the amplitude encoding error in our analysis when aiming for a realistic application. That is why we require more qubits for the algorithm involving amplitude amplification.

Note that $R_z(\theta)$ can be virtually implemented with real physical devices. For superconducting devices, $R_z(\theta)$ indeed does not have to be implemented physically, but rather it can be implemented by changing the phase of the reference frame defined by the multi-level rotating frame. That is, $R_z(\theta)$ is a virtual gate, and therefore, there is no physical error in implementing $R_z(\theta)$.

B. Additional resource estimates

The results for T gates are shown in Fig. 6(a), (b), and (c), corresponding to the tasks with CNOT results in Fig. 4. Fig. 6(d) presents the results when energy gap dependence is taken into account. In the resource estimates, we choose

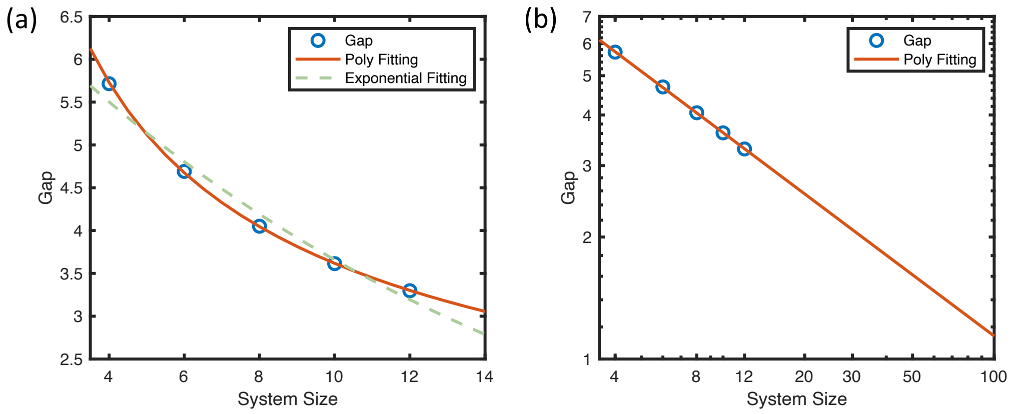

Fig S1. **Gap dependence for the antiferromagnetic Heisenberg model.** (a) Exact gap dependence with increasing system sizes. The polynomial fitting for the gap works better than the exponential fitting. (b) Gap dependence up to 100 qubits by fitting.

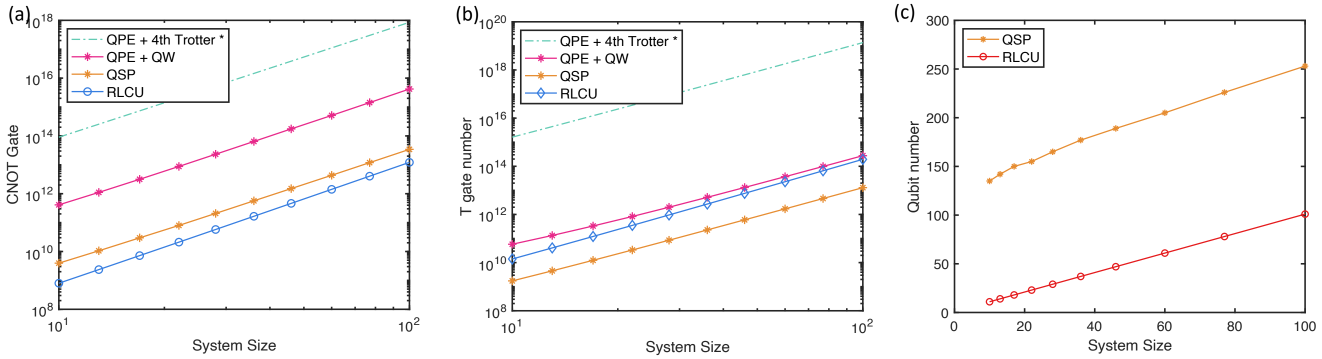

Fig S2. **Resource estimation (the number of cno tgates and T gates) for the eigenstate property estimation for the 2-local Hamiltonian.** The Hamiltonian is $H = \sum_{i,j} X_i X_j + \sum_{i} Z_i$, in which case the commutator relation between different Hamiltonian summands is ignored. Here the 4th-order random Trotter formula is used as it performs the best over other orders, which is marked by an asterisk alongside Trotter in the legend. We compare the gate count involved in different methods. Note that the commutator relation of the Hamiltonian is not taken into account which results in a higher gate count for our method.

to use the compensate the Trotter error up to $2k$th-order. As a result this has a polynomial scaling with $\mathcal{O}(\varepsilon^{-\frac{1}{4k+1}})$. This is easy for the sampling process. Even with this conservative estimation, our method outperforms others as shown in Fig. 6(a). The actual performance may be better in practice.

For general Hamiltonians without considering commutation relations, our method may require more T gates than QSP because our method requires R_z gates, which have a large overhead when they are synthesised into T gates. To verify this point, we consider the two-local Hamiltonian. The results are shown in Fig. S2.

C. Implementation on IBM quantum cloud

In the main text, we consider normalised anisotropic Hamiltonians with parameters $J_x = 1.05$, $J_y = 1$, $J_z = 0.7$ and $h_z = 0.2$ in Fig. 7. The settings are $\tau = 2.5$ and $x_c = 2$. Another experiment instance with fewer samples ($N_s = 1024$ for each circuit run) is shown in Fig. S3.

The experimental results are aligned well with the ideal results. In the following, we give a few comments on the observed noise resilience in practice. First of all, the observed noise resilience can be understood from the sampling structure of our algorithm. Circuits with longer evolution times are deeper and thus more affected by noise, but they are sampled much less frequently because the designed probability distribution. To be concrete, as in Fig. 1(a), both the time t_i and the circuit instance $\vec{i}$ according to their probability distribution in Eq. (1) and Eq. (13). That is, the time is sampled according to non-uniform distribution $\Pr(t_i)$ which quickly decay with time (which ensure the time

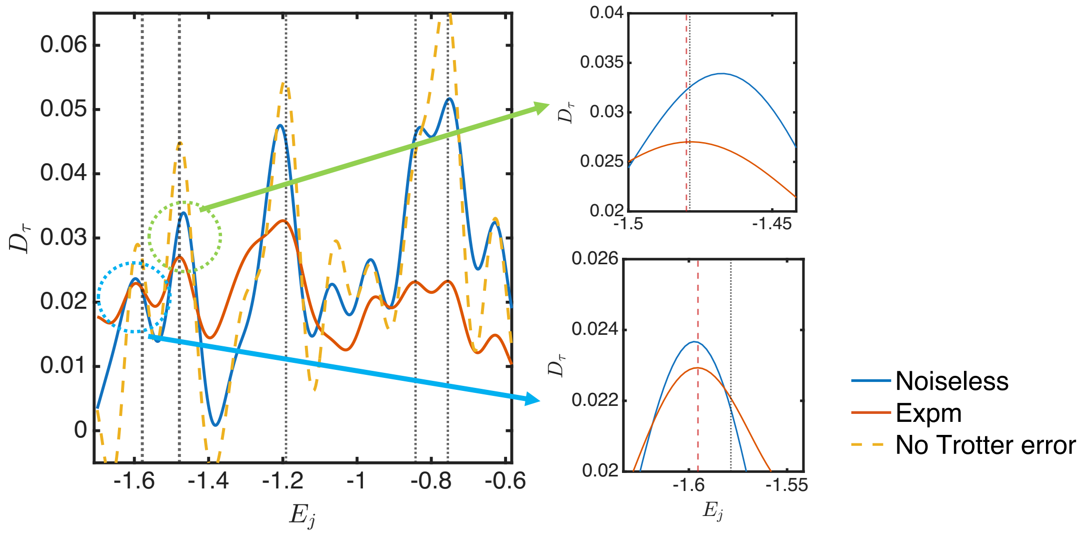

Fig S3. **Searching ground state and first excited state energies of Heisenberg Hamiltonians on IBM quantum devices.** (a) We consider a 12-qubit normalised anisotropic Heisenberg Hamiltonian (see Eq. (8) in the main text), without any external field. All parameter settings are consistent with those in the main text. This represents another circuit instance, using 1024 measurement shots. The figure on the right provides a zoomed-in view of a narrower energy range, highlighting the estimated ground-state and first excited-state energies shown in the left panel.

complexity of our algorithm is small). Therefore, circuit instances with long time t_i (hence more noisy) contribute smaller on the final eigenstate property estimation than the short time ones, i.e, the impact of noise which is more serious in deep circuits is suppressed.

In addition, Hamiltonian simulation may exhibit a certain level of intrinsic noise resilience. As discussed in recent studies, random errors in quantum circuits tend to show concentration behaviour, which brings smaller errors than symmetric errors, meaning that their cumulative effect averages out rather than accumulates coherently. See, for example, the numerical tests in Fig. 1 in [86]. This finding implies that noise may be suppressed with our circuit design. In the intermediate-scale simulation regime, it is interesting to explore whether we can have some error concentration effect so that in this type of quantum algorithm the performance is good.

REFERENCES

1. K. Bharti, A. Cervera-Lierta, T. H. Kyaw, T. Haug, S. Alperin-Lea, A. Anand, M. Degroote, H. Heimonen, J. S. Kottmann, T. Menke, W.-K. Mok, S. Sim, L.-C. Kwek, A. Aspuru-Guzik, Noisy intermediate-scale quantum algorithms. *Rev. Mod. Phys.* **94**, 015004 (2022).

2. B. Bauer, S. Bravyi, M. Motta, G. K.-L. Chan, Quantum algorithms for quantum chemistry and quantum materials science. *Chem. Rev.* **120**, 12685–12717 (2020).

3. A. M. Dalzell, S. M. Ardle, M. Berta, P. Bienias, C.-F. Chen, A. Gilyén, C. T. Hann, M. J. Kastoryano, E. T. Khabiboulline, A. Kubica, G. Salton, S. Wang, F. G. S. L. Brandão, Quantum algorithms: A survey of applications and end-to-end complexities. arXiv:2310.03011 (2023).

4. J. Kempe, A. Kitaev, O. Regev, The complexity of the local Hamiltonian problem. *SIAM J. Comput.* **35**, 1070–1097 (2006).

5. S. Lee, J. Lee, H. Zhai, Y. Tong, A. M. Dalzell, A. Kumar, P. Helms, J. Gray, Z.-H. Cui, W. Liu, M. Kastoryano, R. Babbush, J. Preskill, D. R. Reichman, E. T. Campbell, E. F. Valeev, L. Lin, G. K.-L. Chan, Evaluating the evidence for exponential quantum advantage in ground-state quantum chemistry. *Nat. Commun.* **14**, 1952 (2023).

6. A. Y. Kitaev. Quantum measurements and the abelian stabilizer problem. arXiv:quant-ph/9511026 (1995).

7. B. L. Higgins, D. W. Berry, S. D. Bartlett, H. M. Wiseman, G. J. Pryde, Entanglement-free heisenberg-limited phase estimation. *Nature* **450**, 393–396 (2007).

8. E. Knill, G. Ortiz, R. D. Somma, Optimal quantum measurements of expectation values of observables. *Phys. Rev. A* **75**, 012328 (2007).

9. P. Rall, Faster coherent quantum algorithms for phase, energy, and amplitude estimation. *Quantum* **5**, 566 (2021).

10. R. Meister, and S. C. Benjamin. Resource-frugal Hamiltonian eigenstate preparation via repeated quantum phase estimation measurements. arXiv:2212.00846 (2022).

11. J. M. Martyn, Z. M. Rossi, A. K. Tan, I. L. Chuang, Grand unification of quantum algorithms. *PRX Quantum* **2**, 040203 (2021).

12. Z. Ding, L. Lin, Even shorter quantum circuit for phase estimation on early fault-tolerant quantum computers with applications to ground-state energy estimation. *PRX Quantum* **4**, 020331 (2023).

13. K. Hejazi, J. Soni, M. S. Zini, J. M. Arrazola, Better product formulas for quantum phase estimation. arXiv:2412.16811 (2024).

14. T. Keen, E. Dumitrescu, Y. Wang, Quantum algorithms for ground-state preparation and green's function calculation. arXiv:2112.05731 (2021).

15. S. Chakraborty, Implementing any linear combination of unitaries on intermediate-term quantum computers. *Quantum* **8**, 1496 (2024).

16. Y. Yang, L. Bing-Nan, Y. Li, Accelerated quantum Monte Carlo with mitigated error on noisy quantum computer. *PRX Quantum* **2**, 040361 (2021).

17. K. Wan, M. Berta, E. T. Campbell, A randomized quantum algorithm for statistical phase estimation. arXiv:2110.12071 (2021).

18. G. Wang, D. S. França, G. Rendon, P. D. Johnson, Faster ground state energy estimation on early fault-tolerant quantum computers via rejection sampling. arXiv:2304.09827 (2023).

19. Z. Ding, H. Li, L. Lin, H. K. Ni, L. Ying, R. Zhang, Quantum multiple eigenvalue gaussian filtered search: An efficient and versatile quantum phase estimation method. arXiv:2402.01013 (2024).

20. G. Wang, D. S. França, R. Zhang, S. Zhu, P. D. Johnson, Quantum algorithm for ground state energy estimation using circuit depth with exponentially improved dependence on precision. *Quantum* **7**, 1167 (2023).

21. L. Lin, Y. Tong, Heisenberg-limited ground-state energy estimation for early fault-tolerant quantum computers. *PRX Quantum* **3**, 010318 (2022).

22. P. Zeng, J. Sun, X. Yuan. Universal quantum algorithmic cooling on a quantum computer. arXiv:2109.15304 (2021).

23. S. Lu, M. C. Bañuls, J. Ignacio Cirac, Algorithms for quantum simulation at finite energies. *PRX Quantum* **2**, 020321 (2021).

24. R. Zhang, G. Wang, P. Johnson, Computing ground state properties with early fault-tolerant quantum computers. *Quantum* **6**, 761 (2022).

25. M. Huo, Y. Li, Error-resilient Monte Carlo quantum simulation of imaginary time. *Quantum* **7**, 916 (2023).

26. M.-Q. He, D.-B. Zhang, Z. D. Wang, Quantum gaussian filter for exploring ground-state properties. *Phys. Rev. A* **106**, 032420 (2022).

27. S. Wang, S. McArdle, M. Berta, Qubit-efficient randomized quantum algorithms for linear algebra. *PRX Quantum* **5**, 020324 (2024).

28. L. Lin, Y. Tong, Near-optimal ground state preparation. *Quantum* **4**, 372 (2020).

29. L. Lin, Y. Tong, Optimal polynomial based quantum eigenstate filtering with application to solving quantum linear systems. *Quantum* **4**, 361 (2020).

30. D. An, J.-P. Liu, L. Lin, Linear combination of Hamiltonian simulation for nonunitary dynamics with optimal state preparation cost. *Phys. Rev. Lett.* **131**, 150603 (2023).

31. O. Kiss, U. Azad, B. Requena, A. Roggero, D. Wakeham, J. M. Arrazola, Early fault-tolerant quantum algorithms in practice: Application to ground-state energy estimation. *Quantum* **9**, 1682 (2025).

32. T. S. Cubitt, Dissipative ground state preparation and the dissipative quantum eigensolver. arXiv:2303.11962 (2023).

33. Z. Ding, C.-F. Chen, L. Lin, Single-ancilla ground state preparation via Lindbladians. *Phys. Rev. Res.* **6**, 033147 (2024).

34. C.-F. Chen, H.-Y. Huang, J. Preskill, L. Zhou, "Local minima in quantum systems," in *Proceedings of the 56th Annual ACM Symposium on Theory Of Computing* (ACM, 2024), pp. 1323–1330.

35. W. J. Huggins, B. A. O'Gorman, N. C. Rubin, D. R. Reichman, R. Babbush, J. Lee, Unbiasing fermionic quantum Monte Carlo with a quantum computer. *Nature* **603**, 416–420 (2022).

36. J.-J. Feng, W. Biao, Escaping local minima with quantum circuit coherent cooling. *Phys. Rev. A* **109**, 032405 (2024).

37. L. K. Kovalsky, F. A. Calderon-Vargas, M. D. Grace, A. B. Magann, J. B. Larsen, A. D. Baczewski, M. Sarovar, Self-healing of trotter error in digital adiabatic state preparation. *Phys. Rev. Lett.* **131**, 060602 (2023).

38. M. Motta, C. Sun, A. T. Tan, M. J. O'Rourke, E. Ye, A. J. Minnich, F. G. Brandão, G. K.-L. Chan, Determining eigenstates and thermal states on a quantum computer using quantum imaginary time evolution. *Nat. Phys.* **16**, 205–210 (2020).

39. K. Hejazi, M. Motta, G. K. Chan. Adiabatic quantum imaginary time evolution. arXiv:2308.03292 (2023).

40. A. Katabarwa, K. Gratsea, A. Caesura, and Peter D Johnson. Early fault-tolerant quantum computing. arXiv:2311.14814 (2023).

41. D. Devulapalli, E. Schoute, A. Bapat, A. M. Childs, A. V. Gorshkov, Quantum routing with teleportation. *Phys. Rev. Res.* **6**, 033313 (2022).

42. A. Gilyén, S. Yuan, G. H. Low, N. Wiebe. Quantum singular value transformation and beyond: Exponential improvements for quantum matrix arithmetics. arXiv:1806.01838 (2019).

43. Y. Dong, L. Lin, Y. Tong, Ground-state preparation and energy estimation on early fault-tolerant quantum computers via quantum eigenvalue transformation of unitary matrices. *PRX Quantum* **3**, 040305 (2022).

44. I. H. Kim, Y.-H. Liu, S. Pallister, W. Pol, S. Roberts, E. Lee, Fault-tolerant resource estimate for quantum chemical simulations: Case study on Li-ion battery electrolyte molecules. *Phys. Rev. Res.* **4**, 023019 (2022).

45. M. Reiher, N. Wiebe, K. M. Svore, D. Wecker, M. Troyer, Elucidating reaction mechanisms on quantum computers. *Proc. Natl. Acad. Sci. U.S.A.* **114**, 7555–7560 (2017).

46. J. J. Goings, A. White, J. Lee, C. S. Tautermann, M. Degroote, C. Gidney, T. Shiozaki, R. Babbush, N. C. Rubin, Reliably assessing the electronic structure of cytochrome P450 on today's classical computers and tomorrow's quantum computers. *Proc. Natl. Acad. Sci. U.S.A.* **119**, e2203533119 (2022).

47. D. Wecker, B. Bauer, B. K. Clark, M. B. Hastings, M. Troyer, Gate-count estimates for performing quantum chemistry on small quantum computers. *Phys. Rev. A* **90**, 022305 (2014).

48. E. Campbell, Random compiler for fast Hamiltonian simulation. *Phys. Rev. Lett.* **123**, 070503 (2019).

49. V. von Burg, G. H. Low, T. Häner, D. S. Steiger, M. Reiher, M. Roetteler, M. Troyer, Quantum computing enhanced computational catalysis. *Phys. Rev. Res.* **3**, 033055 (2021).

50. I. D. Kivlichan, C. Gidney, D. W. Berry, N. Wiebe, J. M. Clean, W. Sun, Z. Jiang, N. Rubin, A. Fowler, A. Aspuru-Guzik, H. Neven, R. Babbush, Improved fault-tolerant quantum simulation of condensed-phase correlated electrons via trotterization. *Quantum* **4**, 296 (2020).

51. E. T. Campbell, Early fault-tolerant simulations of the Hubbard model. *Quantum Sci. Technol.* **7**, 015007 (2021).

52. R. Babbush, C. Gidney, D. W. Berry, N. Wiebe, J. McClean, A. Paler, A. Fowler, H. Neven, Encoding electronic spectra in quantum circuits with linear T complexity. *Phys. Rev. X* **8**, 041015 (2018).

53. J. Lee, D. W. Berry, C. Gidney, W. J. Huggins, J. R. McClean, N. Wiebe, R. Babbush, Even more efficient quantum computations of chemistry through tensor hypercontraction. *PRX Quantum* **2**, 030305 (2021).

54. D. W. Berry, Y. Tong, T. Khattar, A. White, T. I. Kim, S. Boixo, L. Lin, S. Lee, G. K.-L. Chan, R. Babbush, N. C. Rubin, Rapid initial-state preparation for the quantum simulation of strongly correlated molecules. *PRX Quantum* **6**, 020327 (2025).

55. Y. Ge, J. Tura, J. Ignacio Cirac, Faster ground state preparation and high-precision ground energy estimation with fewer qubits. *J. Math. Phys.* **60**, 022202 (2019).

56. A. M. Childs, N. Wiebe, Hamiltonian simulation using linear combinations of unitary operations. *Quantum Inf. Comput.* **12**, 901–924 (2012).

57. P. Zeng, J. Sun, L. Jiang, Q. Zhao, Simple and high-precision Hamiltonian simulation by compensating trotter error with linear combination of unitary operations. *PRX Quantum* **6**, 010359 (2025).

58. A. M. Childs, D. Maslov, Y. Nam, N. J. Ross, Y. Su, Toward the first quantum simulation with quantum speedup. *Proc. Natl. Acad. Sci. U.S.A.* **115**, 9456–9461 (2018).

59. A. M. Childs, S. Yuan, M. C. Tran, N. Wiebe, S. Zhu, Theory of trotter error with commutator scaling. *Phys. Rev. X* **11**, 011020 (2021).

60. G. Rendon, J. Watkins, N. Wiebe, Improved accuracy for trotter simulations using Chebyshev interpolation. *Quantum* **8**, 1266 (2024).

61. J. D. Watson, J. Watkins, Exponentially reduced circuit depths using trotter error mitigation. *PRX Quantum* **6**, 030325 (2025).

62. I. D. Kivlichan, J. McClean, N. Wiebe, C. Gidney, A. Aspuru-Guzik, G. K.-L. Chan, R. Babbush, Quantum simulation of electronic structure with linear depth and connectivity. *Phys. Rev. Lett.* **120**, 110501 (2018).

63. R. Babbush, N. Wiebe, J. McClean, J. McClain, H. Neven, G. K.-L. Chan, Low-depth quantum simulation of materials. *Phys. Rev. X* **8**, 011044 (2018).

64. G. H. Low, I. L. Chuang, Hamiltonian simulation by qubitization. *Quantum* **3**, 163 (2019).

65. T. Koma, B. Nachtergaele, The spectral gap of the ferromagnetic XXZ-chain. *Lett. Math. Phys.* **40**, 1–16 (1997).

66. E. T. Campbell, J. O'Gorman, An efficient magic state approach to small angle rotations. *Quantum Sci. Technol.* **1**, 015007 (2016).

67. G. Duclos-Cianci, D. Poulin, Reducing the quantum-computing overhead with complex gate distillation. *Phys. Rev. A* **91**, 042315 (2015).

68. S. Yuan, H.-Y. Huang, E. T. Campbell, Nearly tight trotterization of interacting electrons. *Quantum* **5**, 495 (2021).

69. N. Yoshioka, M. Amico, W. Kirby, P. Jurcevic, A. Dutt, B. Fuller, S. Garion, H. Haas, I. Hamamura, A. Ivrii, R. Majumdar, Z. Minev, M. Motta, B. Pokharel, P. Rivero, K. Sharma, C. J. Wood, A. Javadi-Abhari, A. Mezzacapo, Krylov diagonalization of large many-body Hamiltonians on a quantum processor. *Nat. Commun.* **16**, 5014 (2025).

70. S. Stanisic, J. L. Bosse, F. M. Gambetta, R. A. Santos, W. Mruczkiewicz, T. E. O'Brien, E. Ostby, A. Montanaro, Observing ground-state properties of the Fermi-Hubbard model using a scalable algorithm on a quantum computer. *Nat. Commun.* **13**, 5743 (2022).

71. J. Tang, X. Ruoqian, Y. Ding, X. Xusheng, Y. Ban, M.-H. Yung, A. Pérez-Obiol, G. Platero, X. Chen, Exploring ground states of Fermi-Hubbard model on honeycomb lattices with counterdiabaticity. *npj Quantum Mater.* **9**, 87 (2024).

72. S. Gharibian, F. Le Gall, "Dequantizing the quantum singular value transformation: Hardness and applications to quantum chemistry and the quantum pcp conjecture," in *Proceedings of the 54th Annual ACM SIGACT Symposium on Theory of Computing* (ACM, 2022), pp. 19–32.

73. S. Gharibian, R. Hayakawa, F. Le Gall, T. Morimae, Improved hardness results for the guided local Hamiltonian problem. arXiv:2207.10250 (2022).

74. Y. Zhan, Z. Ding, J. Huhn, J. Gray, J. Preskill, G. K. Chan, L. Lin, Rapid quantum ground state preparation via dissipative dynamics. arXiv:2503.15827 (2025).

75. G. H. Low, N. Wiebe, Hamiltonian simulation in the interaction picture. arXiv:1805.00675 (2018).

76. T. E. O'Brien, S. Polla, N. C. Rubin, W. J. Huggins, S. McArdle, S. Boixo, J. R. McClean, R. Babbush, Error mitigation via verified phase estimation. *PRX Quantum* **2**, 020317 (2021).

77. C. L. Cortes, S. K. Gray, Quantum Krylov subspace algorithms for ground-and excited-state energy estimation. *Phys. Rev. A* **105**, 022417 (2022).

78. D. W. Berry, C. Gidney, M. Motta, J. R. McClean, R. Babbush, Qubitization of arbitrary basis quantum chemistry leveraging sparsity and low rank factorization. *Quantum* **3**, 208 (2019).

79. M. Motta, E. Ye, J. R. McClean, Z. Li, A. J. Minnich, R. Babbush, G. K.-L. Chan, Low rank representations for quantum simulation of electronic structure. *npj Quantum Inf.* **7**, 83 (2021).

80. J. Haah, M. B. Hastings, R. Kothari, G. H. Low, Quantum algorithm for simulating real time evolution of lattice Hamiltonians. *SIAM J. Comput.* **52**, FOCS18-250–FOCS18-284 (2021).

81. N. J. Ross, P. Selinger, Optimal ancilla-free Clifford+T approximation of z-rotations. *Quantum Inf. Comput.* **16**, 901–953 (2016).

82. A. Bocharov, M. Roetteler, K. M. Svore, Efficient synthesis of universal repeat-until-success quantum circuits. *Phys. Rev. Lett.* **114**, 080502 (2015).

83. M. Mosca, P. Mukhopadhyay, A polynomial time and space heuristic algorithm for T-count. *Quantum Sci. Technol.* **7**, 015003 (2021).

84. D. Maslov, Advantages of using relative-phase toffoli gates with an application to multiple control toffoli optimization. *Phys. Rev. A* **93**, 022311 (2016).

85. A. M. Childs, A. Ostrander, S. Yuan, Faster quantum simulation by randomization. *Quantum* **3**, 182 (2019).

86. Yi. Cai, Y. Tong, J. Preskill, Stochastic error cancellation in analog quantum simulation. arXiv:2311.14818 (2023).
